# Uncovering the burden of hidradenitis suppurativa misdiagnosis and underdiagnosis: a machine learning approach

**DOI:** 10.3389/fmedt.2024.1200400

**Published:** 2024-03-25

**Authors:** Joslyn Kirby, Katherine Kim, Marko Zivkovic, Siwei Wang, Vishvas Garg, Akash Danavar, Chao Li, Naijun Chen, Amit Garg

**Affiliations:** ^1^ Department of Dermatology, Penn State Health, Hershey, PA, United States; ^2^ Value and Evidence, AbbVie, Inc., North Chicago, IL, United States; ^3^ Technology and Innovation, Genesis Research, Hoboken, NJ, United States; ^4^ Department of Dermatology, Northwell Health, New Hyde Park, NY, United States

**Keywords:** machine learning, hidradenitis suppurativa, prediction, diagnosis, dermatology, model

## Abstract

Hidradenitis suppurativa (HS) is a chronic inflammatory follicular skin condition that is associated with significant psychosocial and economic burden and a diminished quality of life and work productivity. Accurate diagnosis of HS is challenging due to its unknown etiology, which can lead to underdiagnosis or misdiagnosis that results in increased patient and healthcare system burden. We applied machine learning (ML) to a medical and pharmacy claims database using data from 2000 through 2018 to develop a novel model to better understand HS underdiagnosis on a healthcare system level. The primary results demonstrated that high-performing models for predicting HS diagnosis can be constructed using claims data, with an area under the curve (AUC) of 81%–82% observed among the top-performing models. The results of the models developed in this study could be input into the development of an impact of inaction model that determines the cost implications of HS diagnosis and treatment delay to the healthcare system.

## Introduction

Hidradenitis suppurativa (HS) is a chronic inflammatory follicular skin condition presenting with painful lesions in the intertriginous skin areas, odor, drainage, and disfigurement that contribute to significant psychosocial and pain-related burdens (1, 2). It is also associated with a high comorbidity burden, for which early recognition and management may reduce mortality (3–5).

The prevalence of HS also remains largely unknown and varies across studies due to data collection methods (3). In the United States, studies show its prevalence ranging from 0.05% to 0.90% of the population (3). Globally, prevalence studies report higher results of up to 4.1% (3). Its prevalence may be higher among certain groups; studies suggest female patients, cigarette smokers, and patients with metabolic syndrome (including obesity, elevated triglycerides, low HDL, elevated blood glucose, and hypertension) may have a higher chance of developing HS (6–8).

The pathophysiology of HS is still not fully agreed upon, with current opinion leaning toward follicular hyperkeratosis and dilation followed by follicular rupture and inflammatory response as the primary events leading to the disease (9). With no specific diagnostic tests and unclear histology, the diagnosis of HS is based on three compulsory clinical criteria: skin changes, locations of lesions, and duration. The Hurley clinical staging system, which is used in the diagnostic process, divides HS into three stages. Stage 1 usually presents with painful nodules or boils that progress to recurrent abscesses, sinus tracts, and scarring (Stage 2) (10). Stage 3 is characterized by diffuse or broad involvement, with multiple interconnected sinus tracts and abscesses. The treatment choice depends on the stage of HS at diagnosis, and effective treatment options are often limited. A majority of patients benefit from a combination of medical and surgical management.

A general lack of awareness about HS in the medical community and a notable heterogeneity in the clinical presentation, which is most often confused with cutaneous abscess, may form the basis of poor disease recognition and misdiagnosis (6, 11). Early incorrect hypotheses of an infectious process as the origin of HS influence providers to recommend improved hygiene practices as a mitigation option causing diagnostic delays. A scarcity of dermatology providers, coupled with long wait times and insurance limitations, further amplifies long waiting periods for an accurate diagnosis (12, 13). HS patients suffer from symptoms for 10 years on average prior to accurate diagnosis, during which time they may experience fragmented care and inappropriate management, such as hospital admissions and readmissions for prolonged antibiotic courses directed at acute infections (11, 12, 14). HS underdiagnosis and misdiagnosis also result in increased healthcare system burden, wherein significantly higher costs of managing and treating HS have been observed compared to other inflammatory skin conditions (8, 15). Therefore, there is a need to reduce diagnostic delays by supporting accurate and early recognition of HS to limit disease progression and manage the comorbidity burden (14, 16). Some studies have reported the use of ultrasound (US) imaging as a characterizing diagnostic tool in conjunction with clinical examination to reduce the uncertainty of HS diagnosis and inform on optimal therapeutic strategies, primarily by detecting inflammatory activity and the early subclinical and dermal features of HS and accurately characterizing lesion morphology (17–19). The use of US for diagnosis is a promising approach to both diagnosis and staging; however, its application in the practice setting is limited at present because it has not yet been standardized or validated. Recent reports on other techniques, such as laser speckle contrast analysis (LASCA) or optical coherence tomography (OCT), for HS diagnosis and treatment monitoring indicate the importance of the development of new tools for better HS detection and management (20, 21).

The application of machine learning (ML) to assist in disease recognition has been implemented in different therapeutic areas and may potentially identify undiagnosed or misdiagnosed HS patients. A study by Garg et al. (22) demonstrated a growing need for the development of clinical decision support tools for HS diagnosis. The application of ML to electronic health record (EHR) and claims databases has recently gained traction, with several studies utilizing ML in claims to identify depression, ankylosing spondylitis, cardiomyopathy, dementia, and hepatitis C (23–27).

The study aimed to develop an ML algorithm to identify undiagnosed HS patients among patients with abscess or cellulitis, the diagnoses most commonly rendered incorrectly by clinicians who are less familiar with HS.

## Materials and methods

### Data source

Datasets derived from the IBM MarketScan Research Databases from 2000 to 2018 were used to train and test, and 2018–2019 data were used to validate ML models developed in this study. An exploratory application assessment of ML models was done on 2018 patient data. The database is comprised of fully adjudicated medical and pharmaceutical reimbursement claims from commercial, Medicare, and Medicaid health plans across the United States, covering over 225 million unique patients. It provides a comprehensive longitudinal view of the insured population, including demographics, plan and provider information, inpatient and outpatient diagnoses, procedures, retail and mail-order prescription records, and plan enrollment and participation eligibility dates.

### Patient populations

ML classification models were developed to discern between cases (HS patients) and controls (non-HS patients). Two separate control cohorts included (1) patients with abscesses and (2) cellulitides. These controls were selected because HS patients are most often treated for either of these two conditions before being diagnosed with HS (11, 14). Table 1 presents the patient attrition for case and control cohorts.

**Table 1:** Patient attrition.

Attrition step	HS patients, *N* (%)	Abscess patients, *N* (%)	Cellulitis patients, *N* (%)
Total number of patients between January 2000 and March 2018	411,061 (100%)	1,926,024 (100%)	11,505,177 (100%)
Exclude patients aged <12 years at first diagnosis and missing birth or gender	406,879 (99%)	1,458,597 (76%)	8,226,741 (72%)
Exclude patients who had HS diagnosis in the study period^a^		1,407,307 (73%)	8,050,490 (70%)
Exclude patients without 36 months of pharmacy and medical enrollment prior to and 6 months after the index date	75,540 (18%)	384,843 (20%)	1,949,256 (17%)
Exclude patients with cancer or immunocompromised-related diagnosis/medication codes	55,989 (14%)	278,483 (14%)	1,431,524 (12%)

HS, hidradenitis suppurativa.

^a^ Only applied to abscess and cellulitis patient cohorts.

This case–cohort study included patients with ≥1 HS diagnosis claim [International Classification of Diseases (ICD), Ninth Revision code 705.83 or ICD-10 code L73.2] between January 2000 and March 2018, aged ≥12 years on their first date of HS diagnosis, and with medical and pharmacy enrollment of ≥36 months prior to and ≥6 months after their first date of HS diagnosis (28, 29). The first HS diagnosis claim within the period of interest defined the index date for the cases.

The control cohorts included patients with ≥1 ICD-9/10 diagnosis claim indicating abscess (Supplementary Table S1) or cellulitis (Supplementary Table S2) between January 2000 and March 2018, aged ≥12 years on the abscess or cellulitis diagnosis date, and with pharmacy and medical enrollment of ≥36 months prior to and ≥6 months after the index date. The date of the first abscess or cellulitis diagnosis claim within the period of interest was used as the index date for controls. The patients with HS diagnoses in the database were excluded from the control cohorts. Diagnostic claims for abscess or cellulitis on the extremities, face/neck, and digits were not considered in the control cohorts due to the lower likelihood of these anatomical regions being impacted by HS.

For all cohorts, patients with ≥1 pre-index cancer- or immunocompromised-related diagnosis/medication (determined by the ICD-9/10 codes; Supplementary Tables S3, S4, respectively) were excluded because they were not considered high-risk and to minimize the bias that they would introduce in the HS patient classifier training.

### Modeling approach

**Figure 1:**
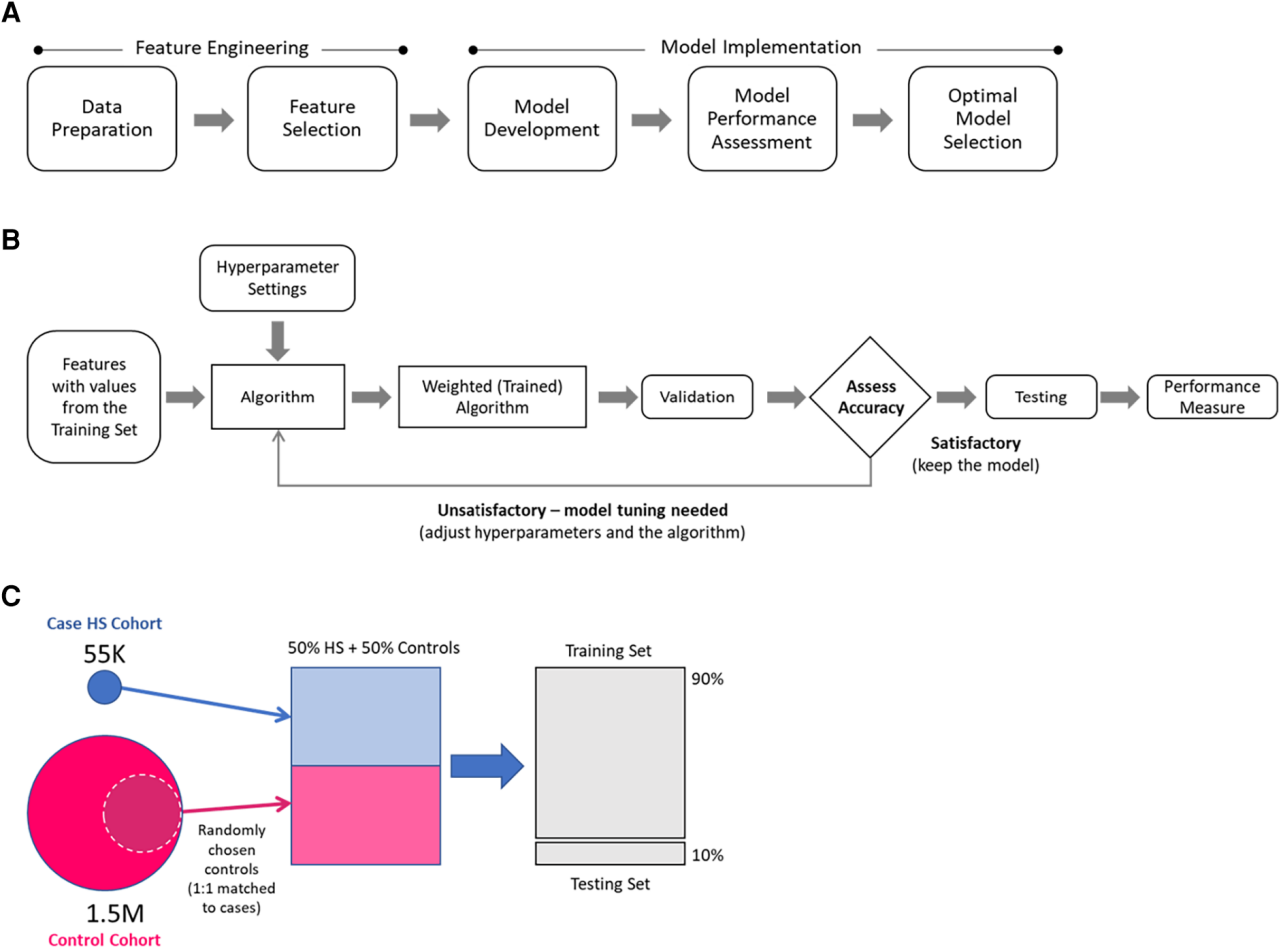
Modeling approach: (**A**) main modeling steps, (**B**) model development overview, (**C**) selection of hidradenitis suppurativa (HS) and control patients for model development.

The modeling approach (Figure 1A) included feature engineering and model implementation. During the feature engineering phase, data preparation and feature selection were performed, including cohort generation, data cleaning and standardization, and feature reduction. In the implementation phase, the models were developed, performance was assessed, and the optimal model was selected. Data preparation utilized SAS software (Version 9.4, SAS Institute, Inc., Cary, NC, USA), whereas feature selection and model implementation were conducted using Python (Version 2.7).

The model development overview is presented in Figure 1B. During the training step, a dataset containing values for the selected features for each patient in case and control training sets was processed to generate weighted mathematical functions (models) that determine the probabilities of a patient being an HS patient. The weighted mathematical function outputs were then assessed (according to the performance measures of interest) for alignment against the known categorization of patients in the test dataset. The function weights were further optimized by adjusting the hyperparameters until a satisfactory performance was attained.

This study considered six single ML algorithm methods—namely, penalized logistic regression (LR) using LASSO, random forest (RF), multilayer perceptron (MLP) neural network, AdaBoost, XGBoost, and LightGBM, and two ensemble methods that combine multiple individual contributing algorithms, i.e., MaxVoting and weighted average. In MaxVoting, each of the four algorithms considered (LR, RF, AdaBoost, and XGBoost) makes a prediction, and the prediction with the highest number of votes is included in the final output. In the weighted average, an ensemble prediction was calculated as the average of the proportionally weighted predictions from single algorithms (LR, XGBoost, and LightGBM). The eight ML algorithm methods were chosen based on their widespread use in ML-based predictive studies, the different biases that they introduce, and their complexity, to select one with the best balance between low complexity, high performance, and fast computational execution (the “optimal model”) (30–36). Satisfactory performance was determined in consultation with dermatologists with a precision/accuracy threshold of 0.7.

The models were developed using selected subsets of case and control cohorts (Figure 1C). For each of the control cohorts, HS cases were matched 1:1 to a random sample of controls, and then 90% of the data were used for the training and 10% for the testing. A separate model that differentiates cases from controls was developed for each case–control training set.

The features used in model development were informed by literature review, the opinion of the clinician, and availability in the underlying data and included demographic and clinical characteristics identified by diagnostic, procedural, and medication codes (at drug class level) in the claims records for case–control cohorts. No derived variables were considered. Each diagnostic, procedural, or medication code identified in the dataset was considered as a separate feature, and no encoding was used. The ICD-9/10 diagnostic codes grouped to the first three digits were considered as binary variables (1/0 = patient had/did not have a claim with a specific diagnosis). To reduce the code burden and noise and provide clinically meaningful categories, we converted the Healthcare Common Procedure Coding System (HCPCS), Current Procedural Terminology (CPT), and ICD Procedure Coding System (ICD PCS) codes to Clinical Classification Software (CCS) procedural categories, which were represented as a frequency of the total number of claims with a specific procedure per patient. Drug classes were derived from Red Book and were included as frequency variables that represent the total number of prescriptions for a specific class per patient. Furthermore, the feature set was limited by filtering out those that occur in <1% of patients in the cohort, removing those with high degrees of mutual association or correlation (based on *T*-test, chi-squared, and phi-coefficient selection), and eliminating them with respect to importance using recursive feature elimination (RFE). Reduction of the initial feature set using the described methods resulted in an approximately three times smaller feature set.

### Performance metrics

Four performance metrics were used to assess and select the optimal model: namely, the area under the curve (AUC), sensitivity, precision, and accuracy. The AUC, ranging from 0 to 1, describes the model accuracy under different thresholds of true and false positives. Sensitivity (recall), a ratio of true positives (HS patients correctly predicted as with HS) and the sum of true positives and false negatives (HS patients incorrectly predicted as without HS), specified the probability of detecting HS among those with the disease. Precision (positive predictive value), a ratio of true positives and the sum of true positives and false positives (patients in control cohorts incorrectly predicted as with HS), indicated the chance that patients with a positive HS prediction truly have HS. Accuracy, a ratio of the sum of true positives and true negatives (patients in control cohorts correctly predicted as without HS) over the total sample size, reflected the overall HS case classification correctness.

### Sensitivity and validation analyses

Two sensitivity analyses, based on two different study periods, were performed for the three top-performing ML models. A “short- vs. long-term” analysis, designed to evaluate the difference in the impact of short-term and long-term features, assigned different weights to the short-term data (data from the records within 1 year of the index date) and the long-term data (from records within 1–3 years of the index date). The second sensitivity analysis, a “short-term” analysis, was conducted by considering only patient data within 1 year of the index date. This second sensitivity analysis was used to verify the predictive power and utility of short-term data, as larger numbers of patients will have 1 year worth of data available in most circumstances.

Validation of models was performed using data from 2018 to 2019; data for patients with known HS diagnoses were fed into the trained models, and an assessment to identify patients with HS was conducted.

### Exploratory application

An exploratory real-world application of three top-performing models was performed to estimate the level of HS underdiagnosis in different US Metropolitan Statistical Areas (MSA). Within a specific MSA, patients in IBM MarketScan Research Databases with an abscess diagnosis in 2018 (index) and 12 months of continuous pre-index enrollment were run through an appropriately trained model. The numbers of ML-predicted HS patients and abscess patients within an MSA were compared to assess the proportion of patients with potential HS misdiagnosis. A high percentage of potentially misdiagnosed HS patients may indicate an extra burden on healthcare systems within an MSA that could be alleviated with a reassessment of patient populations by providers. The same evaluation was repeated for patients with cellulitis diagnosis.

## Results

### Attrition

Among 411,061 patients with HS diagnosis from January 2000 through March 2018, after all selection criteria were applied, 55,989 remained in the HS case cohort. For the control cohorts, following all patient selection criteria, there were 278,483 patients with documented abscesses and 1,431,524 patients with documented cellulitides.

### Base analysis results

The primary results demonstrated that high-performing models for predicting HS diagnosis can be constructed using claims data. The performance comparison for the initial eight ML algorithms is presented in Table 2. Diagnostic accuracy of up to 65% and 73% was achieved among those trained on abscess and cellulitis controls, respectively. Precision was at 60% or above, reaching 80% among cellulitis-trained models. Sensitivity ranged from 55% to 76%, with an AUC of 81%–82% observed among the top-performing models indicating a discriminating ability on par with EHR-trained disease prediction ML models in the literature (32, 34, 37–39). For all algorithms considered, the ML models differentiating HS and cellulitis patients performed better on all metrics as compared to the models trained to differentiate HS and abscess patients. Clinically, HS lesions may be more difficult to differentiate with respect to abscesses given that they are a type of HS lesion, which may explain the underperformance of the abscess-trained models. The three ML models with top-performing algorithms across all cohort analyses and all performance metrics were identified, namely, Model 1 (AdaBoost), Model 2 (LightGBM), and Model 3 (MaxVoting), and these were used in further analyses.

**Table 2:** Performance metrics for all machine learning (ML) methods.

	AUC	Precision	Sensitivity	Accuracy
Base Cohort 1^a^				
LASSO	0.70	0.65	0.66	0.65
Random forest (RF)	0.68	0.64	0.66	0.63
Neural network	0.68	0.59	0.76	0.60
AdaBoost	0.71	0.66	0.66	0.65
XGBoost	0.70	0.66	0.64	0.64
LightGBM	0.70	0.65	0.65	0.64
MaxVoting	0.71	0.67	0.64	0.65
Weighted average	0.71	0.66	0.67	0.65
Base Cohort 2^a^				
LASSO	0.81	0.76	0.69	0.73
Random forest (RF)	0.80	0.74	0.71	0.72
Neural network	0.77	0.80	0.55	0.70
AdaBoost	0.81	0.76	0.69	0.73
XGBoost	0.78	0.75	0.69	0.72
LightGBM	0.81	0.76	0.69	0.73
MaxVoting	0.82	0.78	0.66	0.73
Weighted average	0.82	0.78	0.64	0.73

^a^ Base Cohort 1 includes hidradenitis suppurativa (HS) cases and abscess controls; base Cohort 2 includes HS cases and cellulitis controls.

Age, gender, and risk factors (e.g., overweight and obesity, skin infections/disorders, and skin infection treatment feature types) were the strongest HS-predictive features among the top three models (Table 3). These findings align with the previous knowledge about the disease, from both consultation with HS-treating dermatologists and the existing literature (5, 6). Model 2 also considered the diagnostic feature types (presence of diagnostic claims) as important HS predictors among cellulitis patients. Among all individual algorithms contributing to Model 3, LR was more likely to use features of skin infections/disorders type and characteristics, such as tissue conditions, partial denture, and autogenous arteriovenous fistula, as the most important predictive features. XGBoost algorithm within Model 3 also considered specific comorbidity diagnoses such as osteomyelitis, periostitis, bone infections, nutritional and metabolic disorders, and open wound diagnosis as strong HS predictors. The models trained in Cohort 1 (the subset of the HS and “abscess” cohorts) were more likely to select top features from vaccination, diagnostics, and other comorbidities compared to the models trained to differentiate HS and cellulitis.

**Table 3:** Top 10 predictive features for top-performing models.

Model	Cohort 1 (HS and abscess)	Cohort 2 (HS and cellulitis)
AdaBoost	Age	Age
	Gender	(ICD-9) 682—other cellulitis and abscess
	(ICD-9) 680—carbuncle and furuncle	Gender
	(ICD-9) 278—overweight, obesity, and other hyperalimentation	(CCS) 168—incision and drainage, skin subcutaneous tissue, and fascia
	(ICD-9) 682—other cellulitis and abscess	(ICD-9) 305—non-dependent abuse of drugs
	Antibiotic: tetracyclines (oral)	(ICD-9) 680—carbuncle and furuncle
	(HCPCS) D1203—topical application of fluoride (child)	Sulfonamides: comb NEC (oral)
	(ICD-9) 704—diseases of hair and hair follicles	Antibiotic: tetracyclines (oral)
	(ICD-9) V05—need for other prophylactic vaccination and inoculation against single diseases	(ICD-9) 681—cellulitis and abscess of the finger and toe
	(ICD-9) 758—chromosomal anomalies	(ICD-9) 278—overweight, obesity, and other hyperalimentation
LighGBM	Age	Age
	(ICD-9) V05—need for other prophylactic vaccination and inoculation against single diseases	(CCS) 227—other diagnostic procedures (interview, evaluation, consultation)
	(ICD-9) 278—overweight, obesity, and other hyperalimentation	Gender
	(ICD-9) 682—other cellulitis and abscess	(CCS) 233—laboratory: chemistry and hematology
	Antibiotic: tetracyclines (oral)	Analg antipyr opiate agonists (oral)
	Gender	(CCS) 226—other diagnostic radiology and related techniques
	(ICD-9) V85—body mass index (BMI)	(CCS) 206—microscopic examination (bacterial smear, culture, toxicology)
	(ICD-9) 706—diseases of sebaceous glands	Antibiotic: tetracyclines (oral)
	(ICD-9) 704—diseases of hair and hair follicles	(ICD-9) 682—other cellulitis and abscess
	(ICD-9) 680—carbuncle and furuncle	(ICD-9) 706—diseases of sebaceous glands
MaxVoting (LASSO component)	(ICD-9) 680—carbuncle and furuncle	(ICD-9) 682—other cellulitis and abscess
	(HCPCS) D5850—tissue conditioning, maxillary	(ICD-9) 680—carbuncle and furuncle
	(HCPCS) G8530—autogenous AV fistula received	(ICD-9) 758—chromosomal anomalies
	(ICD-9) V33—twin birth unspecified whether mate liveborn or stillborn	(ICD-9) 704—diseases of hair and hair follicles
	(ICD-9) 758—chromosomal anomalies	(ICD-9) 566—anal and rectal abscess
	(CPT) 2040F—under physical examination	(HCPCS) D5820—interim partial denture (maxillary)
	(HCPCS) D9440—office visit after regularly scheduled hours	(HCPCS) D0999—unspecified diagnostic procedure, by report
	(CPT) 3027F—spirometry test results demonstrate FEV1/FVC ≥70% or patient does not have COPD symptoms	(ICD-9) 685—pilonidal cyst
	(HCPCS) D2381—resin: two surfaces, posterior and primary	(ICD-9) 705—disorders of sweat glands
	(ICD-9) 722.0—Displacement of cervical intervertebral disc without myelopathy	(ICD-9) 624—non-inflammatory disorders of the vulva and perineum
MaxVoting [random forest (RF) component]	Age	(ICD-9) 682—other cellulitis and abscess
	Gender	Age
	(ICD-9) 680—carbuncle and furuncle	Gender
	(CCS) 227—other diagnostic procedures (interview, evaluation, consultation)	(CCS) 168—incision and drainage; skin subcutaneous tissue and fascia
	(CCS) 233—laboratory: chemistry and hematology	Sulfonamides: comb NEC (oral)
	(CCS) 206—microscopic examination (bacterial smear, culture, toxicology)	(CCS) 206—microscopic examination (bacterial smear, culture, toxicology)
	(CCS) 231—other therapeutic procedures	(CCS) 227—other diagnostic procedures
	(CCS) 235—other laboratory	(CCS) 233—laboratory: chemistry and hematology
	(CCS) 226—other diagnostic radiology and related techniques	(ICD-9) 704—diseases of hair and hair follicles
	(CCS) 228—prophylactic vaccinations and inoculations	(ICD-9) 706—diseases of sebaceous glands
MaxVoting (AdaBoost component)	Age	Age
	Gender	(ICD-9) 682—other cellulitis and abscess
	(ICD-9) 680—carbuncle and furuncle	Gender
	(ICD-9) 278—overweight, obesity, and other hyperalimentation	(CCS) 168—incision and drainage; skin subcutaneous tissue and fascia
	(ICD-9) 682—other cellulitis and abscess	(ICD-9) 305—non-dependent abuse of drugs
	Antibiot: tetracyclines (oral)	(ICD-9) 680—carbuncle and furuncle
	(HCPCS) D1203—topical application of fluoride (child)	Sulfonamides: comb NEC (oral)
	(ICD-9) 704—diseases of hair and hair follicles	Antibiot tetracyclines; Oral
	(ICD-9) V05—need for other prophylactic vaccination and inoculation against single diseases	(ICD-9) 681—cellulitis and abscess of finger and toe
	(ICD-9) 758—chromosomal anomalies	(ICD-9) 278—overweight, obesity, and other hyperalimentation
MaxVoting (XGBoost component)	(ICD-9) 680—carbuncle and furuncle	(ICD-9) 682—other cellulitis and abscess
	Gender	(ICD-9) 680—carbuncle and furuncle
	(ICD-9) V05—need for other prophylactic vaccination and inoculation against single diseases	(CPT) 4047F—doc ordered antibio given w/n 1h prior to surg/inc
	(ICD-10) Z3A—weeks of gestation	(ICD-9) 704—diseases of hair and hair follicles
	(ICD-9) 704—diseases of hair and hair follicles	(ICD-9) 707—chronic ulcer of skin
	(ICD-9) 758—chromosomal anomalies	Gender
	(ICD-9) 263—other and unspecified protein–calorie malnutrition	(CCS) 88—abdominal paracentesis
	(ICD-9) 730—osteomyelitis periostitis and other infections involving bone	(ICD-9) 891—open wound of the knee, leg (except thigh), and ankle
	(ICD-10) E90—nutritional and metabolic disorders in diseases classified elsewhere	(ICD-9) 660—obstructed labor
	(ICD-9) 608—other disorders of male genital organs	(ICD-9) 624—non-inflammatory disorders of the vulva and perineum

HS, hidradenitis suppurativa; CCS, clinical classification software; ICD, international classification of diseases; CPT, current procedural terminology; HCPCS, healthcare common procedure coding system; NEC, necrotizing enterocolitis.

### Sensitivity analysis and validation results

Table 4 displays the “short vs. long-term” and “short-term” sensitivity analysis results. The performance metrics are comparable for both sensitivity analyses and similar to the results in Table 2, indicating that data within shorter timeframes of the index date, which may contain more patient samples, can be reliably used for developing models for these types of claims analyses.

**Table 4:** Sensitivity analysis results for the top 3 models.

	Short- vs. long-term		Short-term	
	Sensitivity analysis^a^		Sensitivity analysis^b^	
	Cohort 1 (HS and abscess)	Cohort 2 (HS and cellulitis)	Cohort 1 (HS and abscess)	Cohort 2 (HS and cellulitis)
MaxVoting				
Precision	0.68	0.77	0.64	0.74
Sensitivity	0.65	0.67	0.65	0.70
Accuracy	0.66	0.73	0.64	0.72
AUC	0.72	0.81	0.70	0.80
AdaBoost				
Precision	0.65	0.75	0.62	0.72
Sensitivity	0.68	0.69	0.68	0.72
Accuracy	0.65	0.72	0.63	0.71
AUC	0.71	0.80	0.68	0.80
LightGBM				
Precision	0.67	0.75	0.64	0.73
Sensitivity	0.67	0.70	0.66	0.72
Accuracy	0.66	0.73	0.64	0.72
AUC	0.72	0.81	0.69	0.80

HS, hidradenitis suppurativa; AUC, area under the cruve.

^a^ Assessment of model performance when near-term features (those within 1 year of the index date) are included in the model with a stronger impact than long-term features (those occurring 1 + years prior to the index date).

^b^ Assessment of model performance when only near-term features (those occurring within 1 year of the index date) are considered.

Table 5 presents the validation results. Out of 5,629 patients with their first HS diagnosis in 2018–2019 who satisfied the selection criteria, 5,418 were eligible for input into the models trained on “abscess” controls and 5,477 for input into the models trained on “cellulitis” controls. All top three models performed consistently, predicting 64%–69% of true HS patients. Overall, Models 1 and 2 showed a more robust performance.

**Table 5:** Validation results for the top 3 models—predicting HS diagnosis among patients with known HS diagnosis in 2018–2019.

Models	HS patients predicted as HS *n* (%)
Trained on Cohort 1 (HS and abscess patients)	
AdaBoost	3,665 (66.9%)
LightGBM	3,611 (65.9%)
MaxVoting	3,505 (64.0%)
Trained on Cohort 2 (HS and cellulitis patients)	
AdaBoost	3,727 (68.8%)
LightGBM	3,757 (69.3%)
MaxVoting	3,593 (66.3%)

HS, hidradenitis suppurativa.

### Exploratory application results

**Figure 2:**
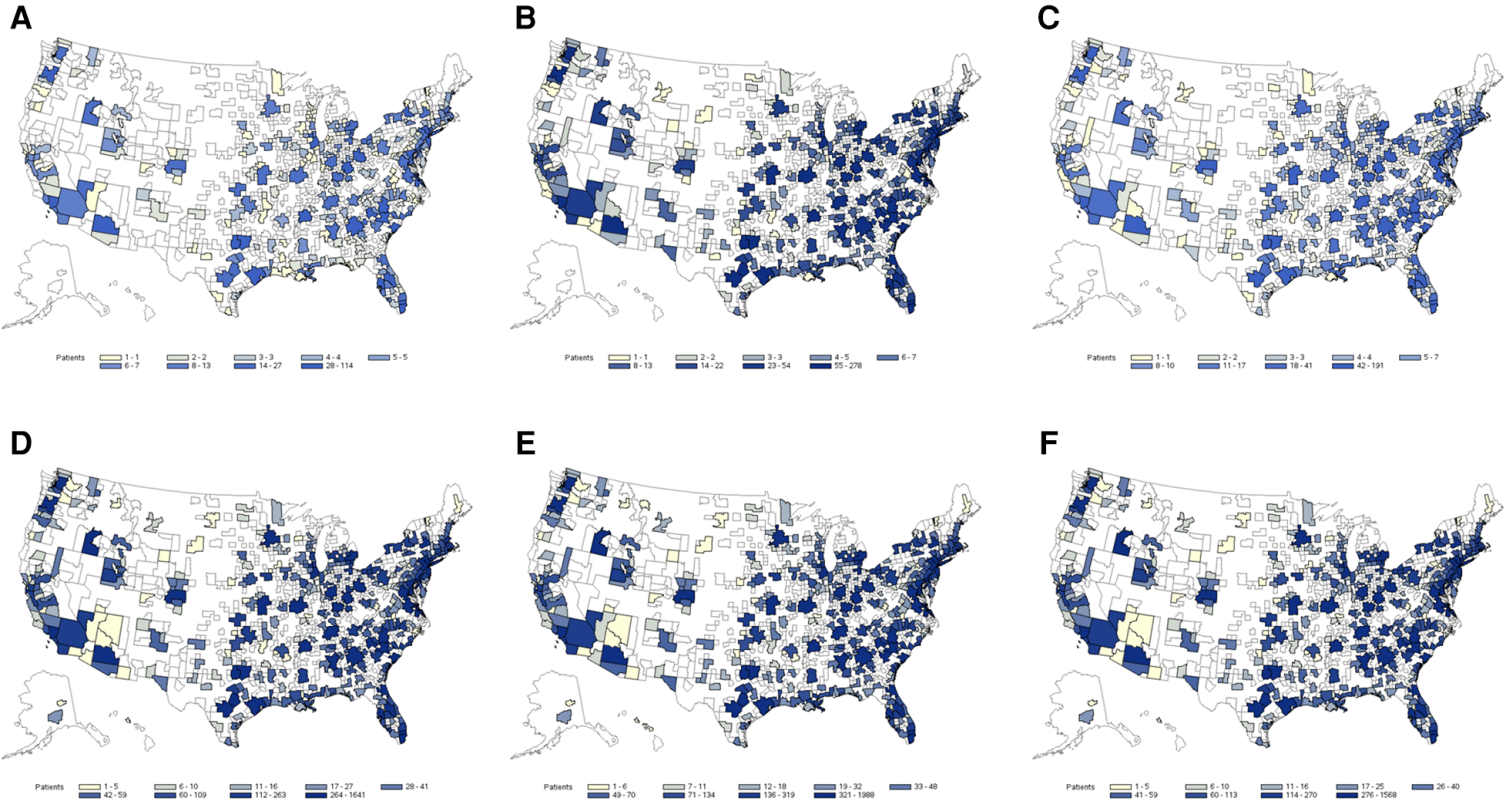
Heat map of estimated hidradenitis suppurativa (HS) underdiagnosis among abscess and cellulitis patients by Metropolitan Statistical Areas (MSA) regions based on the prediction of the three top-performing machine learning (ML) models: (**A**) potential HS cases among abscess patients (AdaBoost), (**B**) potential HS cases among abscess patients (LightGBM), (**C**) potential HS cases among abscess patients (MaxVoting), (**D**) potential HS cases among cellulitis patients (AdaBoost), (**E**) potential HS cases among cellulitis patients (LightGBM), and (**F**) potential HS cases among cellulitis patients (MaxVoting).

The exploratory application indicated a noticeable HS underdiagnosis among abscess or cellulitis patients. The percentage for underdiagnosis varied by MSA and model used. It was smaller among the abscess population than the cellulitis population, reaching 13% of abscess patients in MSAs with the highest level of underdiagnosis. Among cellulitis patients, underdiagnosis was as high as 50% in some MSAs. Metro areas with larger populations had the highest number of predicted HS patients, but there was no obvious relationship observed between the size of the abscess or cellulitis patient populations and the proportion of underdiagnosed HS. Heatmaps of HS prediction among identified abscess and cellulitis patients are presented in Figure 2. This application indicates that the utilization of developed ML models by health systems may be able to identify large pools of underdiagnosed HS patients for further evaluation and diagnosis and clinical and translational research.

## Discussion

HS has poor recognition as a disease entity among both clinicians and patients. A clinical decision support that prompts consideration of HS and distinguishes it from cutaneous abscess and cellulitis may improve gaps in quality, timeliness, and specificity of care. In this study, we used 18 years of claims data to develop an ML model to predict HS among patients with abscesses and cellulitides. The performance of the models demonstrated the utility of the application of ML to claims data, opening another avenue for improving HS patient care.

All three models with the highest performance contain state-of-the-art boosting algorithms that often outperform the traditional algorithms (e.g., LR and RF) frequently used in healthcare analyses. Although these three models gave relatively consistent results among cohorts, the models exhibited differences in prediction performance based on their prediction functions. No single model performed the best across all measures and in all populations; however, the models based on the cellulitis control cohort outperformed those based on the abscess cohort, regardless of model type, reflecting the increased difficulty of discerning HS from abscess compared with cellulitis. Model 2 gave a more conservative result than the other two models, while Model 1 showed the most consistent performance across different testing populations. Given the sparseness of data and the large number of variables often encountered for patients with rare conditions in claims data, the ability of Model 1 to better operate in such conditions gives it an advantage over the other two models. Additionally, Model 1 has higher robustness, as it tends to weigh difficult-to-discern cases more heavily during the training, promoting their impact on model behavior. Of note, Models 1 and 2 had similar, if not better, performance compared with that of Model 3, suggesting the importance of not overlooking simpler models in favor of complicated, ensemble models in these kinds of applications. Furthermore, the two single models had faster execution times, with Model 1 requiring the least optimization and parameter tuning during the training, giving Model 1 an overall recommendation over the other two models.

As demonstrated, ML model performance varies based on the specific cohort used for training, including case and control definitions. Using clinical expert engagement in the early model development stages to appropriately identify populations of interest (e.g., HS vs. abscess patients as compared to HS vs. general dermatology patients) and setting desired optimization targets for model performance helps the resulting models to perform better and be more translatable for clinical use. Performance optimization (trade-off between accuracy, sensitivity, and precision) is dependent on the goal of the model use (screening or confirmation). The trade-off should be investigated when setting model probability threshold levels for classifying a patient as an HS patient.

This study focused on HS detection, but the utilized claims-based ML approach could be extended to the additional indications with appropriate retraining, particularly those hampered by small sample sizes in regular studies. The exploratory application using 2018 IBM MarketScan data showed high potential for identification of HS underdiagnosis or misdiagnosis, with uneven underdiagnosis across regions. The heatmap approach used in the exploratory analysis could spur further identification of regions/centers where the potential for underdiagnoses of HS patients may be high, and further HS medical education or intervention may be warranted. In such cases, it should be kept in mind that the regional distribution assessment results of HS prediction are somewhat reflective of the underlying regional data distribution, which for IBM MarketScan skews toward the southern/southeastern regions and major urban centers.

ML prediction studies for other diseases generally utilize EHR data or specialized datasets as opposed to claims data. Most of the ML-based prediction in dermatology has focused on image-trained models. Claims databases may be more useful than EHRs since they capture a larger population, allowing for larger training, testing, and validation datasets. Although EHRs often contain clinically meaningful data that allow easier model development, they are often limited in population size and characteristics and tend to capture the perspective of a single institution or network, which may produce results with limited relevance to other health provider systems or regions. Additionally, claims sources generally have the benefit of more complete data compared to many EHRs and ensure confidence in the relevance and generalizability of outcomes to a broader population. EHR-linked claims combination might further improve prediction performance due to the availability of clinical variables such as disease severity, procedure or test results, and laboratory values.

This study has several strengths and limitations. A large claims database over 18 years was used, resulting in large samples of HS cases and controls. Several ML models were initially considered, with careful training, testing, and validation and thoughtfully considered model inputs. Sensitivity analyses further gauged prediction, and an exploratory analysis showed the potential for real-world application. The challenges unique to ML may limit generalizability and real-world applicability. A potential limitation to expanded use may be that the application of trained models requires data with the same structure as used in model development, adding a data management burden. Furthermore, the algorithms selected as “best-performing” for the population from claims data considered in this study may not extend to other settings. Other algorithms may work more effectively in different populations if differences from the study population are large. As with any claims data, potential medical coding errors may affect the model performance. Contextual embedding and considerations of temporal relations between patient claims (e.g., time since the first presentation with dermatological symptoms), not considered in this study, could further improve the performance.

In summary, we have described the development of a clinical decision support model that predicts the probability of HS diagnosis and distinguishes it from cutaneous abscess and cellulitis, the most common mimics of HS; it has the potential to improve recognition of HS and reduce diagnostic delay. The results of the models developed can also be applied to the impact of the inaction model that determines cost implications for diagnosis and treatment delay, e.g., cost burden to healthcare systems. Testing on additional external datasets, followed by testing in a clinical setting and against a dermatologist diagnosis of HS, is recommended to confirm and optimize the model performance and its use in this fashion.

## Data Availability

The original contributions presented in the study are included in the article/**Supplementary Material**, and further inquiries can be directed to the corresponding author.
