# Supplementary material for: Uncovering the burden of hidradenitis suppurativa misdiagnosis and underdiagnosis: a machine learning approach
Source: Front Med Technol. 2024 Mar 25;6:1200400. doi: 10.3389/fmedt.2024.1200400 (PMC10999681; doi:10.3389/fmedt.2024.1200400)
Supplement: Supplementary file 1 [file Datasheet1.docx]

**Supplemental Tables/Figures**

**Supplemental Table 1 – Abscess Diagnostic Codes**

| Abscess ICD-9 Diagnostic Codes | |
| --- | --- |
| 680.2 | Carbuncle and furuncle of trunk |
| 680.3 | Carbuncle and furuncle of upper arm and forearm |
| 680.5 | Carbuncle and furuncle of buttock |
| 680.6 | Carbuncle and furuncle of leg – except foot |
| 680.8 | Carbuncle and furuncle of other specified sites |
| 680.9 | Carbuncle and furuncle of unspecified site |
| Abscess ICD-10 Diagnostic Codes | |
| L02211 | Cutaneous abscess of abdominal wall |
| L02212 | Cutaneous abscess of back [any part, except buttock] |
| L02213 | Cutaneous abscess of chest wall |
| L02214 | Cutaneous abscess of groin |
| L02215 | Cutaneous abscess of perineum |
| L02216 | Cutaneous abscess of umbilicus |
| L02219 | Cutaneous abscess of trunk, unspecified |
| L02221 | Furuncle of abdominal wall |
| L02222 | Furuncle of back [any part, except buttock] |
| L02223 | Furuncle of chest wall |
| L02224 | Furuncle of groin |
| L02225 | Furuncle of perineum |
| L02226 | Furuncle of umbilicus |
| L02229 | Furuncle of trunk, unspecified |
| L02231 | Carbuncle of abdominal wall |
| L02232 | Carbuncle of back [any part, except buttock] |
| L02233 | Carbuncle of chest wall |
| L02234 | Carbuncle of groin |
| L02235 | Carbuncle of perineum |
| L02236 | Carbuncle of umbilicus |
| L02239 | Carbuncle of trunk, unspecified |
| L0231 | Cutaneous abscess of buttock |
| L0232 | Furuncle of buttock |
| L0233 | Carbuncle of buttock |
| L02411 | Cutaneous abscess of right axilla |
| L02412 | Cutaneous abscess of left axilla |
| L02413 | Cutaneous abscess of right upper limb |
| L02414 | Cutaneous abscess of left upper limb |
| L02415 | Cutaneous abscess of right lower limb |
| L02416 | Cutaneous abscess of left lower limb |
| L02419 | Cutaneous abscess of limb, unspecified |
| L02421 | Furuncle of right axilla |
| L02422 | Furuncle of left axilla |
| L02423 | Furuncle of right upper limb |
| L02424 | Furuncle of left upper limb |
| L02425 | Furuncle of right lower limb |
| L02426 | Furuncle of left lower limb |
| L02429 | Furuncle of limb, unspecified |
| L02431 | Carbuncle of right axilla |
| L02432 | Carbuncle of left axilla |
| L02433 | Carbuncle of right upper limb |
| L02434 | Carbuncle of left upper limb |
| L02435 | Carbuncle of right lower limb |
| L02436 | Carbuncle of left lower limb |
| L02439 | Carbuncle of limb, unspecified |
| L02818 | Cutaneous abscess of other sites |
| L02828 | Furuncle of other sites |
| L02838 | Carbuncle of other sites |
| L0291 | Cutaneous abscess, unspecified |
| L0292 | Furuncle, unspecified |
| L0293 | Carbuncle, unspecified |

**Supplemental Table 2 – Cellulitis Diagnostic Codes**

| Cellulitis ICD-9 Diagnostic Codes | |
| --- | --- |
| 682.2 | Cellulitis and abscess of trunk |
| 682.3 | Cellulitis and abscess of upper arm and forearm |
| 682.5 | Cellulitis and abscess of buttock |
| 682.6 | Cellulitis and abscess of leg – except foot |
| 682.8 | Cellulitis and abscess of other specified sites |
| 682.9 | Cellulitis and abscess of unspecified sites |
| Cellulitis ICD-10 Diagnostic Codes | |
| L03111 | Cellulitis of right axilla |
| L03112 | Cellulitis of left axilla |
| L03113 | Cellulitis of right upper limb |
| L03114 | Cellulitis of left upper limb |
| L03115 | Cellulitis of right lower limb |
| L03116 | Cellulitis of left lower limb |
| L03119 | Cellulitis of unspecified part of limb |
| L03311 | Cellulitis of abdominal wall |
| L03312 | Cellulitis of back [any part except buttock] |
| L03313 | Cellulitis of chest wall |
| L03314 | Cellulitis of groin |
| L03315 | Cellulitis of perineum |
| L03316 | Cellulitis of umbilicus |
| L03317 | Cellulitis of buttock |
| L03319 | Cellulitis of trunk, unspecified |
| L03818 | Cellulitis of other sites |
| L0390 | Cellulitis, unspecified |

**Supplemental Table 3 – Cancer-Related Diagnostic Codes**

| **Cancer-Related ICD-9 Codes** | |
| --- | --- |
| 179 | Malignant neoplasm of uterus - part unspecified |
| 181 | Malignant neoplasm of placenta |
| 185 | Malignant neoplasm of prostate |
| 193 | Malignant neoplasm of thyroid gland |
| 217 | Benign neoplasm of breast |
| 220 | Benign neoplasm of ovary |
| 226 | Benign neoplasm of thyroid glands |
| 1400 | Malignant neoplasm of upper lip - vermilion border |
| 1401 | Malignant neoplasm of lower lip - vermilion border |
| 1403 | Malignant neoplasm of upper lip - inner aspect |
| 1404 | Malignant neoplasm of lower lip - inner aspect |
| 1405 | Malignant neoplasm of lip - unspecified - inner aspect |
| 1406 | Malignant neoplasm of commissure of lip |
| 1408 | Malignant neoplasm of other sites of lip |
| 1409 | Malignant neoplasm of lip - unspecified - vermilion border |
| 1410 | Malignant neoplasm of base of tongue |
| 1411 | Malignant neoplasm of dorsal surface of tongue |
| 1412 | Malignant neoplasm of tip and lateral border of tongue |
| 1413 | Malignant neoplasm of ventral surface of tongue |
| 1414 | Malignant neoplasm of anterior two-thirds of tongue - part unspecified |
| 1415 | Malignant neoplasm of junctional zone of tongue |
| 1416 | Malignant neoplasm of lingual tonsil |
| 1418 | Malignant neoplasm of other sites of tongue |
| 1419 | Malignant neoplasm of tongue - unspecified |
| 1420 | Malignant neoplasm of parotid gland |
| 1421 | Malignant neoplasm of submandibular gland |
| 1422 | Malignant neoplasm of sublingual gland |
| 1428 | Malignant neoplasm of other major salivary glands |
| 1429 | Malignant neoplasm of salivary gland - unspecified |
| 1430 | Malignant neoplasm of upper gum |
| 1431 | Malignant neoplasm of lower gum |
| 1438 | Malignant neoplasm of other sites of gum |
| 1439 | Malignant neoplasm of gum - unspecified |
| 1440 | Malignant neoplasm of anterior portion of floor of mouth |
| 1441 | Malignant neoplasm of lateral portion of floor of mouth |
| 1448 | Malignant neoplasm of other sites of floor of mouth |
| 1449 | Malignant neoplasm of floor of mouth - part unspecified |
| 1450 | Malignant neoplasm of cheek mucosa |
| 1451 | Malignant neoplasm of vestibule of mouth |
| 1452 | Malignant neoplasm of hard palate |
| 1453 | Malignant neoplasm of soft palate |
| 1454 | Malignant neoplasm of uvula |
| 1455 | Malignant neoplasm of palate - unspecified |
| 1456 | Malignant neoplasm of retromolar area |
| 1458 | Malignant neoplasm of other specified parts of mouth |
| 1459 | Malignant neoplasm of mouth - unspecified |
| 1460 | Malignant neoplasm of tonsil |
| 1461 | Malignant neoplasm of tonsillar fossa |
| 1462 | Malignant neoplasm of tonsillar pillars (anterior) (posterior) |
| 1463 | Malignant neoplasm of vallecula epiglottica |
| 1464 | Malignant neoplasm of anterior aspect of epiglottis |
| 1465 | Malignant neoplasm of junctional region of oropharynx |
| 1466 | Malignant neoplasm of lateral wall of oropharynx |
| 1467 | Malignant neoplasm of posterior wall of oropharynx |
| 1468 | Malignant neoplasm of other specified sites of oropharynx |
| 1469 | Malignant neoplasm of oropharynx - unspecified site |
| 1470 | Malignant neoplasm of superior wall of nasopharynx |
| 1471 | Malignant neoplasm of posterior wall of nasopharynx |
| 1472 | Malignant neoplasm of lateral wall of nasopharynx |
| 1473 | Malignant neoplasm of anterior wall of nasopharynx |
| 1478 | Malignant neoplasm of other specified sites of nasopharynx |
| 1479 | Malignant neoplasm of nasopharynx - unspecified site |
| 1480 | Malignant neoplasm of postcricoid region of hypopharynx |
| 1481 | Malignant neoplasm of pyriform sinus |
| 1482 | Malignant neoplasm of aryepiglottic fold - hypopharyngeal aspect |
| 1483 | Malignant neoplasm of posterior hypopharyngeal wall |
| 1488 | Malignant neoplasm of other specified sites of hypopharynx |
| 1489 | Malignant neoplasm of hypopharynx - unspecified site |
| 1490 | Malignant neoplasm of pharynx - unspecified |
| 1491 | Malignant neoplasm of waldeyer's ring |
| 1498 | Malignant neoplasm of other sites within the lip and oral cavity |
| 1499 | Malignant neoplasm of ill-defined sites within the lip and oral cavity |
| 1500 | Malignant neoplasm of cervical esophagus |
| 1501 | Malignant neoplasm of thoracic esophagus |
| 1502 | Malignant neoplasm of abdominal esophagus |
| 1503 | Malignant neoplasm of upper third of esophagus |
| 1504 | Malignant neoplasm of middle third of esophagus |
| 1505 | Malignant neoplasm of lower third of esophagus |
| 1508 | Malignant neoplasm of other specified part of esophagus |
| 1509 | Malignant neoplasm of esophagus - unspecified site |
| 1510 | Malignant neoplasm of cardia |
| 1511 | Malignant neoplasm of pylorus |
| 1512 | Malignant neoplasm of pyloric antrum |
| 1513 | Malignant neoplasm of fundus of stomach |
| 1514 | Malignant neoplasm of body of stomach |
| 1515 | Malignant neoplasm of lesser curvature of stomach - unspecified |
| 1516 | Malignant neoplasm of greater curvature of stomach - unspecified |
| 1518 | Malignant neoplasm of other specified sites of stomach |
| 1519 | Malignant neoplasm of stomach - unspecified site |
| 1520 | Malignant neoplasm of duodenum |
| 1521 | Malignant neoplasm of jejunum |
| 1522 | Malignant neoplasm of ileum |
| 1523 | Malignant neoplasm of Meckel's diverticulum |
| 1528 | Malignant neoplasm of other specified sites of small intestine |
| 1529 | Malignant neoplasm of small intestine - unspecified site |
| 1530 | Malignant neoplasm of hepatic flexure |
| 1531 | Malignant neoplasm of transverse colon |
| 1532 | Malignant neoplasm of descending colon |
| 1533 | Malignant neoplasm of sigmoid colon |
| 1534 | Malignant neoplasm of cecum |
| 1535 | Malignant neoplasm of appendix vermiformis |
| 1536 | Malignant neoplasm of ascending colon |
| 1537 | Malignant neoplasm of splenic flexure |
| 1538 | Malignant neoplasm of other specified sites of large intestine |
| 1539 | Malignant neoplasm of colon - unspecified site |
| 1540 | Malignant neoplasm of rectosigmoid junction |
| 1541 | Malignant neoplasm of rectum |
| 1542 | Malignant neoplasm of anal canal |
| 1543 | Malignant neoplasm of anus - unspecified site |
| 1548 | Malignant neoplasm of other sites of rectum - rectosigmoid junction - and anus |
| 1550 | Malignant neoplasm of liver - primary |
| 1551 | Malignant neoplasm of intrahepatic bile ducts |
| 1552 | Malignant neoplasm of liver - not specified as primary or secondary |
| 1560 | Malignant neoplasm of gallbladder |
| 1561 | Malignant neoplasm of extrahepatic bile ducts |
| 1562 | Malignant neoplasm of ampulla of vater |
| 1568 | Malignant neoplasm of other specified sites of gallbladder and extrahepatic bile ducts |
| 1569 | Malignant neoplasm of biliary tract - part unspecified site |
| 1570 | Malignant neoplasm of head of pancreas |
| 1571 | Malignant neoplasm of body of pancreas |
| 1572 | Malignant neoplasm of tail of pancreas |
| 1573 | Malignant neoplasm of pancreatic duct |
| 1574 | Malignant neoplasm of islets of langerhans |
| 1578 | Malignant neoplasm of other specified sites of pancreas |
| 1579 | Malignant neoplasm of pancreas - part unspecified |
| 1580 | Malignant neoplasm of retroperitoneum |
| 1588 | Malignant neoplasm of specified parts of peritoneum |
| 1589 | Malignant neoplasm of peritoneum - unspecified |
| 1590 | Malignant neoplasm of intestinal tract - part unspecified |
| 1591 | Malignant neoplasm of spleen - not elsewhere classified |
| 1598 | Malignant neoplasm of other sites of digestive system and intra-abdominal organs |
| 1599 | Malignant neoplasm of ill-defined sites within the digestive organs and peritoneum |
| 1600 | Malignant neoplasm of nasal cavities |
| 1601 | Malignant neoplasm of auditory tube - middle ear - and mastoid air cells |
| 1602 | Malignant neoplasm of maxillary sinus |
| 1603 | Malignant neoplasm of ethmoidal sinus |
| 1604 | Malignant neoplasm of frontal sinus |
| 1605 | Malignant neoplasm of sphenoidal sinus |
| 1608 | Malignant neoplasm of other accessory sinuses |
| 1609 | Malignant neoplasm of accessory sinus - unspecified |
| 1610 | Malignant neoplasm of glottis |
| 1611 | Malignant neoplasm of supraglottis |
| 1612 | Malignant neoplasm of subglottis |
| 1613 | Malignant neoplasm of laryngeal cartilages |
| 1618 | Malignant neoplasm of other specified sites of larynx |
| 1619 | Malignant neoplasm of larynx - unspecified |
| 1620 | Malignant neoplasm of trachea |
| 1622 | Malignant neoplasm of main bronchus |
| 1623 | Malignant neoplasm of upper lobe - bronchus or lung |
| 1624 | Malignant neoplasm of middle lobe - bronchus or lung |
| 1625 | Malignant neoplasm of lower lobe - bronchus or lung |
| 1628 | Malignant neoplasm of other parts of bronchus or lung |
| 1629 | Malignant neoplasm of bronchus and lung - unspecified |
| 1630 | Malignant neoplasm of parietal pleura |
| 1631 | Malignant neoplasm of visceral pleura |
| 1638 | Malignant neoplasm of other specified sites of pleura |
| 1639 | Malignant neoplasm of pleura - unspecified |
| 1640 | Malignant neoplasm of thymus |
| 1641 | Malignant neoplasm of heart |
| 1642 | Malignant neoplasm of anterior mediastinum |
| 1643 | Malignant neoplasm of posterior mediastinum |
| 1648 | Malignant neoplasm of other parts of mediastinum |
| 1649 | Malignant neoplasm of mediastinum - part unspecified |
| 1650 | Malignant neoplasm of upper respiratory tract - part unspecified |
| 1658 | Malignant neoplasm of other sites within the respiratory system and intrathoracic organs |
| 1659 | Malignant neoplasm of ill-defined sites within the respiratory system |
| 1700 | Malignant neoplasm of bones of skull and face - except mandible |
| 1701 | Malignant neoplasm of mandible |
| 1702 | Malignant neoplasm of vertebral column - excluding sacrum and coccyx |
| 1703 | Malignant neoplasm of ribs - sternum - and clavicle |
| 1704 | Malignant neoplasm of scapula and long bones of upper limb |
| 1705 | Malignant neoplasm of short bones of upper limb |
| 1706 | Malignant neoplasm of pelvic bones - sacrum - and coccyx |
| 1707 | Malignant neoplasm of long bones of lower limb |
| 1708 | Malignant neoplasm of short bones of lower limb |
| 1709 | Malignant neoplasm of bone and articular cartilage - site unspecified |
| 1710 | Malignant neoplasm of connective and other soft tissue of head - face - and neck |
| 1712 | Malignant neoplasm of connective and other soft tissue of upper limb - including shoulder |
| 1713 | Malignant neoplasm of connective and other soft tissue of lower limb - including hip |
| 1714 | Malignant neoplasm of connective and other soft tissue of thorax |
| 1715 | Malignant neoplasm of connective and other soft tissue of abdomen |
| 1716 | Malignant neoplasm of connective and other soft tissue of pelvis |
| 1717 | Malignant neoplasm of connective and other soft tissue of trunk - unspecified |
| 1718 | Malignant neoplasm of other specified sites of connective and other soft tissue |
| 1719 | Malignant neoplasm of connective and other soft tissue - site unspecified |
| 1720 | Malignant melanoma of skin of lip |
| 1721 | Malignant melanoma of skin of eyelid - including canthus |
| 1722 | Malignant melanoma of skin of ear and external auditory canal |
| 1723 | Malignant melanoma of skin of other and unspecified parts of face |
| 1724 | Malignant melanoma of skin of scalp and neck |
| 1725 | Malignant melanoma of skin of trunk - except scrotum |
| 1726 | Malignant melanoma of skin of upper limb - including shoulder |
| 1727 | Malignant melanoma of skin of lower limb - including hip |
| 1728 | Malignant melanoma of other specified sites of skin |
| 1729 | Melanoma of skin - site unspecified |
| 1740 | Malignant neoplasm of nipple and areola of female breast |
| 1741 | Malignant neoplasm of central portion of female breast |
| 1742 | Malignant neoplasm of upper-inner quadrant of female breast |
| 1743 | Malignant neoplasm of lower-inner quadrant of female breast |
| 1744 | Malignant neoplasm of upper-outer quadrant of female breast |
| 1745 | Malignant neoplasm of lower-outer quadrant of female breast |
| 1746 | Malignant neoplasm of axillary tail of female breast |
| 1748 | Malignant neoplasm of other specified sites of female breast |
| 1749 | Malignant neoplasm of breast (female) - unspecified |
| 1750 | Malignant neoplasm of nipple and areola of male breast |
| 1759 | Malignant neoplasm of other and unspecified sites of male breast |
| 1760 | Kaposi's sarcoma - skin |
| 1761 | Kaposi's sarcoma - soft tissue |
| 1762 | Kaposi's sarcoma - palate |
| 1763 | Kaposi's sarcoma - gastrointestinal sites |
| 1764 | Kaposi's sarcoma - lung |
| 1765 | Kaposi's sarcoma - lymph nodes |
| 1768 | Kaposi's sarcoma - other specified sites |
| 1769 | Kaposi's sarcoma - unspecified site |
| 1800 | Malignant neoplasm of endocervix |
| 1801 | Malignant neoplasm of exocervix |
| 1808 | Malignant neoplasm of other specified sites of cervix |
| 1809 | Malignant neoplasm of cervix uteri - unspecified site |
| 1820 | Malignant neoplasm of corpus uteri - except isthmus |
| 1821 | Malignant neoplasm of isthmus |
| 1828 | Malignant neoplasm of other specified sites of body of uterus |
| 1830 | Malignant neoplasm of ovary |
| 1832 | Malignant neoplasm of fallopian tube |
| 1833 | Malignant neoplasm of broad ligament of uterus |
| 1834 | Malignant neoplasm of parametrium |
| 1835 | Malignant neoplasm of round ligament of uterus |
| 1838 | Malignant neoplasm of other specified sites of uterine adnexa |
| 1839 | Malignant neoplasm of uterine adnexa - unspecified site |
| 1840 | Malignant neoplasm of vagina |
| 1841 | Malignant neoplasm of labia majora |
| 1842 | Malignant neoplasm of labia minora |
| 1843 | Malignant neoplasm of clitoris |
| 1844 | Malignant neoplasm of vulva - unspecified site |
| 1848 | Malignant neoplasm of other specified sites of female genital organs |
| 1849 | Malignant neoplasm of female genital organ - site unspecified |
| 1860 | Malignant neoplasm of undescended testis |
| 1869 | Malignant neoplasm of other and unspecified testis |
| 1871 | Malignant neoplasm of prepuce |
| 1872 | Malignant neoplasm of glans penis |
| 1873 | Malignant neoplasm of body of penis |
| 1874 | Malignant neoplasm of penis - part unspecified |
| 1875 | Malignant neoplasm of epididymis |
| 1876 | Malignant neoplasm of spermatic cord |
| 1877 | Malignant neoplasm of scrotum |
| 1878 | Malignant neoplasm of other specified sites of male genital organs |
| 1879 | Malignant neoplasm of male genital organ - site unspecified |
| 1880 | Malignant neoplasm of trigone of urinary bladder |
| 1881 | Malignant neoplasm of dome of urinary bladder |
| 1882 | Malignant neoplasm of lateral wall of urinary bladder |
| 1883 | Malignant neoplasm of anterior wall of urinary bladder |
| 1884 | Malignant neoplasm of posterior wall of urinary bladder |
| 1885 | Malignant neoplasm of bladder neck |
| 1886 | Malignant neoplasm of ureteric orifice |
| 1887 | Malignant neoplasm of urachus |
| 1888 | Malignant neoplasm of other specified sites of bladder |
| 1889 | Malignant neoplasm of bladder - part unspecified |
| 1890 | Malignant neoplasm of kidney - except pelvis |
| 1891 | Malignant neoplasm of renal pelvis |
| 1892 | Malignant neoplasm of ureter |
| 1893 | Malignant neoplasm of urethra |
| 1894 | Malignant neoplasm of paraurethral glands |
| 1898 | Malignant neoplasm of other specified sites of urinary organs |
| 1899 | Malignant neoplasm of urinary organ - site unspecified |
| 1900 | Malignant neoplasm of eyeball - except conjunctiva - cornea - retina - and choroid |
| 1901 | Malignant neoplasm of orbit |
| 1902 | Malignant neoplasm of lacrimal gland |
| 1903 | Malignant neoplasm of conjunctiva |
| 1904 | Malignant neoplasm of cornea |
| 1905 | Malignant neoplasm of retina |
| 1906 | Malignant neoplasm of choroid |
| 1907 | Malignant neoplasm of lacrimal duct |
| 1908 | Malignant neoplasm of other specified sites of eye |
| 1909 | Malignant neoplasm of eye - part unspecified |
| 1910 | Malignant neoplasm of cerebrum - except lobes and ventricles |
| 1911 | Malignant neoplasm of frontal lobe |
| 1912 | Malignant neoplasm of temporal lobe |
| 1913 | Malignant neoplasm of parietal lobe |
| 1914 | Malignant neoplasm of occipital lobe |
| 1915 | Malignant neoplasm of ventricles |
| 1916 | Malignant neoplasm of cerebellum nos |
| 1917 | Malignant neoplasm of brain stem |
| 1918 | Malignant neoplasm of other parts of brain |
| 1919 | Malignant neoplasm of brain - unspecified |
| 1920 | Malignant neoplasm of cranial nerves |
| 1921 | Malignant neoplasm of cerebral meninges |
| 1922 | Malignant neoplasm of spinal cord |
| 1923 | Malignant neoplasm of spinal meninges |
| 1928 | Malignant neoplasm of other specified sites of nervous system |
| 1929 | Malignant neoplasm of nervous system - part unspecified |
| 1940 | Malignant neoplasm of adrenal gland |
| 1941 | Malignant neoplasm of parathyroid gland |
| 1943 | Malignant neoplasm of pituitary gland and craniopharyngeal duct |
| 1944 | Malignant neoplasm of pineal gland |
| 1945 | Malignant neoplasm of carotid body |
| 1946 | Malignant neoplasm of aortic body and other paraganglia |
| 1948 | Malignant neoplasm of other endocrine glands and related structures |
| 1949 | Malignant neoplasm of endocrine gland - site unspecified |
| 1950 | Malignant neoplasm of head - face - and neck |
| 1951 | Malignant neoplasm of thorax |
| 1952 | Malignant neoplasm of abdomen |
| 1953 | Malignant neoplasm of pelvis |
| 1954 | Malignant neoplasm of upper limb |
| 1955 | Malignant neoplasm of lower limb |
| 1958 | Malignant neoplasm of other specified sites |
| 1960 | Secondary and unspecified malignant neoplasm of lymph nodes of head - face - and neck |
| 1961 | Secondary and unspecified malignant neoplasm of intrathoracic lymph nodes |
| 1962 | Secondary and unspecified malignant neoplasm of intra-abdominal lymph nodes |
| 1963 | Secondary and unspecified malignant neoplasm of lymph nodes of axilla and upper limb |
| 1965 | Secondary and unspecified malignant neoplasm of lymph nodes of inguinal region and lower limb |
| 1966 | Secondary and unspecified malignant neoplasm of intrapelvic lymph nodes |
| 1968 | Secondary and unspecified malignant neoplasm of lymph nodes of multiple sites |
| 1969 | Secondary and unspecified malignant neoplasm of lymph nodes - site unspecified |
| 1970 | Secondary malignant neoplasm of lung |
| 1971 | Secondary malignant neoplasm of mediastinum |
| 1972 | Secondary malignant neoplasm of pleura |
| 1973 | Secondary malignant neoplasm of other respiratory organs |
| 1974 | Secondary malignant neoplasm of small intestine including duodenum |
| 1975 | Secondary malignant neoplasm of large intestine and rectum |
| 1976 | Secondary malignant neoplasm of retroperitoneum and peritoneum |
| 1977 | Malignant neoplasm of liver - secondary |
| 1978 | Secondary malignant neoplasm of other digestive organs and spleen |
| 1980 | Secondary malignant neoplasm of kidney |
| 1981 | Secondary malignant neoplasm of other urinary organs |
| 1982 | Secondary malignant neoplasm of skin |
| 1983 | Secondary malignant neoplasm of brain and spinal cord |
| 1984 | Secondary malignant neoplasm of other parts of nervous system |
| 1985 | Secondary malignant neoplasm of bone and bone marrow |
| 1986 | Secondary malignant neoplasm of ovary |
| 1987 | Secondary malignant neoplasm of adrenal gland |
| 1990 | Disseminated malignant neoplasm without specification of site |
| 1991 | Other malignant neoplasm without specification of site |
| 1992 | Malignant neoplasm associated with transplant organ |
| 2100 | Benign neoplasm of lip |
| 2101 | Benign neoplasm of tongue |
| 2102 | Benign neoplasm of major salivary glands |
| 2103 | Benign neoplasm of floor of mouth |
| 2104 | Benign neoplasm of other and unspecified parts of mouth |
| 2105 | Benign neoplasm of tonsil |
| 2106 | Benign neoplasm of other parts of oropharynx |
| 2107 | Benign neoplasm of nasopharynx |
| 2108 | Benign neoplasm of hypopharynx |
| 2109 | Benign neoplasm of pharynx - unspecified |
| 2110 | Benign neoplasm of esophagus |
| 2111 | Benign neoplasm of stomach |
| 2112 | Benign neoplasm of duodenum - jejunum - and ileum |
| 2113 | Benign neoplasm of colon |
| 2114 | Benign neoplasm of rectum and anal canal |
| 2115 | Benign neoplasm of liver and biliary passages |
| 2116 | Benign neoplasm of pancreas - except islets of Langerhans |
| 2117 | Benign neoplasm of islets of Langerhans |
| 2118 | Benign neoplasm of retroperitoneum and peritoneum |
| 2119 | Benign neoplasm of other and unspecified site in the digestive system |
| 2120 | Benign neoplasm of nasal cavities - middle ear - and accessory sinuses |
| 2121 | Benign neoplasm of larynx |
| 2122 | Benign neoplasm of trachea |
| 2123 | Benign neoplasm of bronchus and lung |
| 2124 | Benign neoplasm of pleura |
| 2125 | Benign neoplasm of mediastinum |
| 2126 | Benign neoplasm of thymus |
| 2127 | Benign neoplasm of heart |
| 2128 | Benign neoplasm of other specified sites of respiratory and intrathoracic organs |
| 2129 | Benign neoplasm of respiratory and intrathoracic organs - site unspecified |
| 2130 | Benign neoplasm of bones of skull and face |
| 2131 | Benign neoplasm of lower jaw bone |
| 2132 | Benign neoplasm of vertebral column - excluding sacrum and coccyx |
| 2133 | Benign neoplasm of ribs - sternum - and clavicle |
| 2134 | Benign neoplasm of scapula and long bones of upper limb |
| 2135 | Benign neoplasm of short bones of upper limb |
| 2136 | Benign neoplasm of pelvic bones - sacrum - and coccyx |
| 2137 | Benign neoplasm of long bones of lower limb |
| 2138 | Benign neoplasm of short bones of lower limb |
| 2139 | Benign neoplasm of bone and articular cartilage - site unspecified |
| 2140 | Lipoma of skin and subcutaneous tissue of face |
| 2141 | Lipoma of other skin and subcutaneous tissue |
| 2142 | Lipoma of intrathoracic organs |
| 2143 | Lipoma of intra-abdominal organs |
| 2144 | Lipoma of spermatic cord |
| 2148 | Lipoma of other specified sites |
| 2149 | Lipoma - unspecified site |
| 2150 | Other benign neoplasm of connective and other soft tissue of head - face - and neck |
| 2152 | Other benign neoplasm of connective and other soft tissue of upper limb - including shoulder |
| 2153 | Other benign neoplasm of connective and other soft tissue of lower limb - including hip |
| 2154 | Other benign neoplasm of connective and other soft tissue of thorax |
| 2155 | Other benign neoplasm of connective and other soft tissue of abdomen |
| 2156 | Other benign neoplasm of connective and other soft tissue of pelvis |
| 2157 | Other benign neoplasm of connective and other soft tissue of trunk - unspecified |
| 2158 | Other benign neoplasm of connective and other soft tissue of other specified sites |
| 2159 | Other benign neoplasm of connective and other soft tissue - site unspecified |
| 2160 | Benign neoplasm of skin of lip |
| 2161 | Benign neoplasm of eyelid - including canthus |
| 2162 | Benign neoplasm of ear and external auditory canal |
| 2163 | Benign neoplasm of skin of other and unspecified parts of face |
| 2164 | Benign neoplasm of scalp and skin of neck |
| 2165 | Benign neoplasm of skin of trunk - except scrotum |
| 2166 | Benign neoplasm of skin of upper limb - including shoulder |
| 2167 | Benign neoplasm of skin of lower limb - including hip |
| 2168 | Benign neoplasm of other specified sites of skin |
| 2169 | Benign neoplasm of skin - site unspecified |
| 2180 | Submucous leiomyoma of uterus |
| 2181 | Intramural leiomyoma of uterus |
| 2182 | Subserous leiomyoma of uterus |
| 2189 | Leiomyoma of uterus - unspecified |
| 2190 | Benign neoplasm of cervix uteri |
| 2191 | Benign neoplasm of corpus uteri |
| 2198 | Benign neoplasm of other specified parts of uterus |
| 2199 | Benign neoplasm of uterus - part unspecified |
| 2210 | Benign neoplasm of fallopian tube and uterine ligaments |
| 2211 | Benign neoplasm of vagina |
| 2212 | Benign neoplasm of vulva |
| 2218 | Benign neoplasm of other specified sites of female genital organs |
| 2219 | Benign neoplasm of female genital organ - site unspecified |
| 2220 | Benign neoplasm of testis |
| 2221 | Benign neoplasm of penis |
| 2222 | Benign neoplasm of prostate |
| 2223 | Benign neoplasm of epididymis |
| 2224 | Benign neoplasm of scrotum |
| 2228 | Benign neoplasm of other specified sites of male genital organs |
| 2229 | Benign neoplasm of male genital organ - site unspecified |
| 2230 | Benign neoplasm of kidney - except pelvis |
| 2231 | Benign neoplasm of renal pelvis |
| 2232 | Benign neoplasm of ureter |
| 2233 | Benign neoplasm of bladder |
| 2239 | Benign neoplasm of urinary organ - site unspecified |
| 2240 | Benign neoplasm of eyeball - except conjunctiva - cornea - retina - and choroid |
| 2241 | Benign neoplasm of orbit |
| 2242 | Benign neoplasm of lacrimal gland |
| 2243 | Benign neoplasm of conjunctiva |
| 2244 | Benign neoplasm of cornea |
| 2245 | Benign neoplasm of retina |
| 2246 | Benign neoplasm of choroid |
| 2247 | Benign neoplasm of lacrimal duct |
| 2248 | Benign neoplasm of other specified parts of eye |
| 2249 | Benign neoplasm of eye - part unspecified |
| 2250 | Benign neoplasm of brain |
| 2251 | Benign neoplasm of cranial nerves |
| 2252 | Benign neoplasm of cerebral meninges |
| 2253 | Benign neoplasm of spinal cord |
| 2254 | Benign neoplasm of spinal meninges |
| 2258 | Benign neoplasm of other specified sites of nervous system |
| 2259 | Benign neoplasm of nervous system - part unspecified |
| 2270 | Benign neoplasm of adrenal gland |
| 2271 | Benign neoplasm of parathyroid gland |
| 2273 | Benign neoplasm of pituitary gland and craniopharyngeal duct |
| 2274 | Benign neoplasm of pineal gland |
| 2275 | Benign neoplasm of carotid body |
| 2276 | Benign neoplasm of aortic body and other paraganglia |
| 2278 | Benign neoplasm of other endocrine glands and related structures |
| 2279 | Benign neoplasm of endocrine gland - site unspecified |
| 2281 | Lymphangioma - any site |
| 2290 | Benign neoplasm of lymph nodes |
| 2298 | Benign neoplasm of other specified sites |
| 2299 | Benign neoplasm of unspecified site |
| 2300 | Carcinoma in situ of lip - oral cavity - and pharynx |
| 2301 | Carcinoma in situ of esophagus |
| 2302 | Carcinoma in situ of stomach |
| 2303 | Carcinoma in situ of colon |
| 2304 | Carcinoma in situ of rectum |
| 2305 | Carcinoma in situ of anal canal |
| 2306 | Carcinoma in situ of anus - unspecified |
| 2307 | Carcinoma in situ of other and unspecified parts of intestine |
| 2308 | Carcinoma in situ of liver and biliary system |
| 2309 | Carcinoma in situ of other and unspecified digestive organs |
| 2310 | Carcinoma in situ of larynx |
| 2311 | Carcinoma in situ of trachea |
| 2312 | Carcinoma in situ of bronchus and lung |
| 2318 | Carcinoma in situ of other specified parts of respiratory system |
| 2319 | Carcinoma in situ of respiratory system - part unspecified |
| 2320 | Carcinoma in situ of skin of lip |
| 2321 | Carcinoma in situ of eyelid - including canthus |
| 2322 | Carcinoma in situ of skin of ear and external auditory canal |
| 2323 | Carcinoma in situ of skin of other and unspecified parts of face |
| 2324 | Carcinoma in situ of scalp and skin of neck |
| 2325 | Carcinoma in situ of skin of trunk - except scrotum |
| 2326 | Carcinoma in situ of skin of upper limb - including shoulder |
| 2327 | Carcinoma in situ of skin of lower limb - including hip |
| 2328 | Carcinoma in situ of other specified sites of skin |
| 2329 | Carcinoma in situ of skin - site unspecified |
| 2330 | Carcinoma in situ of breast |
| 2331 | Carcinoma in situ of cervix uteri |
| 2332 | Carcinoma in situ of other and unspecified parts of uterus |
| 2334 | Carcinoma in situ of prostate |
| 2335 | Carcinoma in situ of penis |
| 2336 | Carcinoma in situ of other and unspecified male genital organs |
| 2337 | Carcinoma in situ of bladder |
| 2339 | Carcinoma in situ of other and unspecified urinary organs |
| 2340 | Carcinoma in situ of eye |
| 2348 | Carcinoma in situ of other specified sites |
| 2349 | Carcinoma in situ - site unspecified |
| 2350 | Neoplasm of uncertain behavior of major salivary glands |
| 2351 | Neoplasm of uncertain behavior of lip - oral cavity - and pharynx |
| 2352 | Neoplasm of uncertain behavior of stomach - intestines - and rectum |
| 2353 | Neoplasm of uncertain behavior of liver and biliary passages |
| 2354 | Neoplasm of uncertain behavior of retroperitoneum and peritoneum |
| 2355 | Neoplasm of uncertain behavior of other and unspecified digestive organs |
| 2356 | Neoplasm of uncertain behavior of larynx |
| 2357 | Neoplasm of uncertain behavior of trachea - bronchus - and lung |
| 2358 | Neoplasm of uncertain behavior of pleura - thymus - and mediastinum |
| 2359 | Neoplasm of uncertain behavior of other and unspecified respiratory organs |
| 2360 | Neoplasm of uncertain behavior of uterus |
| 2361 | Neoplasm of uncertain behavior of placenta |
| 2362 | Neoplasm of uncertain behavior of ovary |
| 2363 | Neoplasm of uncertain behavior of other and unspecified female genital organs |
| 2364 | Neoplasm of uncertain behavior of testis |
| 2365 | Neoplasm of uncertain behavior of prostate |
| 2366 | Neoplasm of uncertain behavior of other and unspecified male genital organs |
| 2367 | Neoplasm of uncertain behavior of bladder |
| 2370 | Neoplasm of uncertain behavior of pituitary gland and craniopharyngeal duct |
| 2371 | Neoplasm of uncertain behavior of pineal gland |
| 2372 | Neoplasm of uncertain behavior of adrenal gland |
| 2373 | Neoplasm of uncertain behavior of paraganglia |
| 2374 | Neoplasm of uncertain behavior of other and unspecified endocrine glands |
| 2375 | Neoplasm of uncertain behavior of brain and spinal cord |
| 2376 | Neoplasm of uncertain behavior of meninges |
| 2379 | Neoplasm of uncertain behavior of other and unspecified parts of nervous system |
| 2380 | Neoplasm of uncertain behavior of bone and articular cartilage |
| 2381 | Neoplasm of uncertain behavior of connective and other soft tissue |
| 2382 | Neoplasm of uncertain behavior of skin |
| 2383 | Neoplasm of uncertain behavior of breast |
| 2384 | Polycythemia vera |
| 2385 | Neoplasm of uncertain behavior of histiocytic and mast cells |
| 2386 | Neoplasm of uncertain behavior of plasma cells |
| 2388 | Neoplasm of uncertain behavior of other specified sites |
| 2389 | Neoplasm of uncertain behavior - site unspecified |
| 2390 | Neoplasm of unspecified nature of digestive system |
| 2391 | Neoplasm of unspecified nature of respiratory system |
| 2392 | Neoplasm of unspecified nature of bone - soft tissue - and skin |
| 2393 | Neoplasm of unspecified nature of breast |
| 2394 | Neoplasm of unspecified nature of bladder |
| 2395 | Neoplasm of unspecified nature of other genitourinary organs |
| 2396 | Neoplasm of unspecified nature of brain |
| 2397 | Neoplasm of unspecified nature of endocrine glands and other parts of nervous system |
| 2399 | Neoplasm of unspecified nature - site unspecified |
| 17300 | Unspecified malignant neoplasm of skin of lip |
| 17301 | Basal cell carcinoma of skin of lip |
| 17302 | Squamous cell carcinoma of skin of lip |
| 17309 | Other specified malignant neoplasm of skin of lip |
| 17310 | Unspecified malignant neoplasm of eyelid - including canthus |
| 17311 | Basal cell carcinoma of eyelid - including canthus |
| 17312 | Squamous cell carcinoma of eyelid - including canthus |
| 17319 | Other specified malignant neoplasm of eyelid - including canthus |
| 17320 | Unspecified malignant neoplasm of skin of ear and external auditory canal |
| 17321 | Basal cell carcinoma of skin of ear and external auditory canal |
| 17322 | Squamous cell carcinoma of skin of ear and external auditory canal |
| 17329 | Other specified malignant neoplasm of skin of ear and external auditory canal |
| 17330 | Unspecified malignant neoplasm of skin of other and unspecified parts of face |
| 17331 | Basal cell carcinoma of skin of other and unspecified parts of face |
| 17332 | Squamous cell carcinoma of skin of other and unspecified parts of face |
| 17339 | Other specified malignant neoplasm of skin of other and unspecified parts of face |
| 17340 | Unspecified malignant neoplasm of scalp and skin of neck |
| 17341 | Basal cell carcinoma of scalp and skin of neck |
| 17342 | Squamous cell carcinoma of scalp and skin of neck |
| 17349 | Other specified malignant neoplasm of scalp and skin of neck |
| 17350 | Unspecified malignant neoplasm of skin of trunk - except scrotum |
| 17351 | Basal cell carcinoma of skin of trunk - except scrotum |
| 17352 | Squamous cell carcinoma of skin of trunk - except scrotum |
| 17359 | Other specified malignant neoplasm of skin of trunk - except scrotum |
| 17360 | Unspecified malignant neoplasm of skin of upper limb - including shoulder |
| 17361 | Basal cell carcinoma of skin of upper limb - including shoulder |
| 17362 | Squamous cell carcinoma of skin of upper limb - including shoulder |
| 17369 | Other specified malignant neoplasm of skin of upper limb - including shoulder |
| 17370 | Unspecified malignant neoplasm of skin of lower limb - including hip |
| 17371 | Basal cell carcinoma of skin of lower limb - including hip |
| 17372 | Squamous cell carcinoma of skin of lower limb - including hip |
| 17379 | Other specified malignant neoplasm of skin of lower limb - including hip |
| 17380 | Unspecified malignant neoplasm of other specified sites of skin |
| 17381 | Basal cell carcinoma of other specified sites of skin |
| 17382 | Squamous cell carcinoma of other specified sites of skin |
| 17389 | Other specified malignant neoplasm of other specified sites of skin |
| 17390 | Unspecified malignant neoplasm of skin - site unspecified |
| 17391 | Basal cell carcinoma of skin - site unspecified |
| 17392 | Squamous cell carcinoma of skin - site unspecified |
| 17399 | Other specified malignant neoplasm of skin - site unspecified |
| 19881 | Secondary malignant neoplasm of breast |
| 19882 | Secondary malignant neoplasm of genital organs |
| 19889 | Secondary malignant neoplasm of other specified sites |
| 20000 | Reticulosarcoma - unspecified site - extranodal and solid organ sites |
| 20001 | Reticulosarcoma - lymph nodes of head - face - and neck |
| 20002 | Reticulosarcoma - intrathoracic lymph nodes |
| 20003 | Reticulosarcoma - intra-abdominal lymph nodes |
| 20004 | Reticulosarcoma - lymph nodes of axilla and upper limb |
| 20005 | Reticulosarcoma - lymph nodes of inguinal region and lower limb |
| 20006 | Reticulosarcoma - intrapelvic lymph nodes |
| 20007 | Reticulosarcoma - spleen |
| 20008 | Reticulosarcoma - lymph nodes of multiple sites |
| 20010 | Lymphosarcoma - unspecified site - extranodal and solid organ sites |
| 20011 | Lymphosarcoma - lymph nodes of head - face - and neck |
| 20012 | Lymphosarcoma - intrathoracic lymph nodes |
| 20013 | Lymphosarcoma - intra-abdominal lymph nodes |
| 20014 | Lymphosarcoma - lymph nodes of axilla and upper limb |
| 20015 | Lymphosarcoma - lymph nodes of inguinal region and lower limb |
| 20016 | Lymphosarcoma - intrapelvic lymph nodes |
| 20017 | Lymphosarcoma - spleen |
| 20018 | Lymphosarcoma - lymph nodes of multiple sites |
| 20020 | Burkitt's tumor or lymphoma - unspecified site - extranodal and solid organ sites |
| 20021 | Burkitt's tumor or lymphoma - lymph nodes of head - face - and neck |
| 20022 | Burkitt's tumor or lymphoma - intrathoracic lymph nodes |
| 20023 | Burkitt's tumor or lymphoma - intra-abdominal lymph nodes |
| 20024 | Burkitt's tumor or lymphoma - lymph nodes of axilla and upper limb |
| 20025 | Burkitt's tumor or lymphoma - lymph nodes of inguinal region and lower limb |
| 20026 | Burkitt's tumor or lymphoma - intrapelvic lymph nodes |
| 20027 | Burkitt's tumor or lymphoma - spleen |
| 20028 | Burkitt's tumor or lymphoma - lymph nodes of multiple sites |
| 20030 | Marginal zone lymphoma - unspecified site - extranodal and solid organ sites |
| 20031 | Marginal zone lymphoma - lymph nodes of head - face - and neck |
| 20032 | Marginal zone lymphoma - intrathoracic lymph nodes |
| 20033 | Marginal zone lymphoma - intraabdominal lymph nodes |
| 20034 | Marginal zone lymphoma - lymph nodes of axilla and upper limb |
| 20035 | Marginal zone lymphoma - lymph nodes of inguinal region and lower limb |
| 20036 | Marginal zone lymphoma - intrapelvic lymph nodes |
| 20037 | Marginal zone lymphoma - spleen |
| 20038 | Marginal zone lymphoma - lymph nodes of multiple sites |
| 20040 | Mantle cell lymphoma - unspecified site - extranodal and solid organ sites |
| 20041 | Mantle cell lymphoma - lymph nodes of head - face - and neck |
| 20042 | Mantle cell lymphoma - intrathoracic lymph nodes |
| 20043 | Mantle cell lymphoma - intra-abdominal lymph nodes |
| 20044 | Mantle cell lymphoma - lymph nodes of axilla and upper limb |
| 20045 | Mantle cell lymphoma - lymph nodes of inguinal region and lower limb |
| 20046 | Mantle cell lymphoma - intrapelvic lymph nodes |
| 20047 | Mantle cell lymphoma - spleen |
| 20048 | Mantle cell lymphoma - lymph nodes of multiple sites |
| 20050 | Primary central nervous system lymphoma - unspecified site - extranodal and solid organ sites |
| 20051 | Primary central nervous system lymphoma - lymph nodes of head - face - and neck |
| 20052 | Primary central nervous system lymphoma - intrathoracic lymph nodes |
| 20053 | Primary central nervous system lymphoma - intra-abdominal lymph nodes |
| 20054 | Primary central nervous system lymphoma - lymph nodes of axilla and upper limb |
| 20055 | Primary central nervous system lymphoma - lymph nodes of inguinal region and lower limb |
| 20056 | Primary central nervous system lymphoma - intrapelvic lymph nodes |
| 20057 | Primary central nervous system lymphoma - spleen |
| 20058 | Primary central nervous system lymphoma - lymph nodes of multiple sites |
| 20060 | Anaplastic large cell lymphoma - unspecified site - extranodal and solid organ sites |
| 20061 | Anaplastic large cell lymphoma - lymph nodes of head - face - and neck |
| 20062 | Anaplastic large cell lymphoma - intrathoracic lymph nodes |
| 20063 | Anaplastic large cell lymphoma - intra-abdominal lymph nodes |
| 20064 | Anaplastic large cell lymphoma - lymph nodes of axilla and upper limb |
| 20065 | Anaplastic large cell lymphoma - lymph nodes of inguinal region and lower limb |
| 20066 | Anaplastic large cell lymphoma - intrapelvic lymph nodes |
| 20067 | Anaplastic large cell lymphoma - spleen |
| 20068 | Anaplastic large cell lymphoma - lymph nodes of multiple sites |
| 20070 | Large cell lymphoma - unspecified site - extranodal and solid organ sites |
| 20071 | Large cell lymphoma - lymph nodes of head - face - and neck |
| 20072 | Large cell lymphoma - intrathoracic lymph nodes |
| 20073 | Large cell lymphoma - intra-abdominal lymph nodes |
| 20074 | Large cell lymphoma - lymph nodes of axilla and upper limb |
| 20075 | Large cell lymphoma - lymph nodes of inguinal region and lower limb |
| 20076 | Large cell lymphoma - intrapelvic lymph nodes |
| 20077 | Large cell lymphoma - spleen |
| 20078 | Large cell lymphoma - lymph nodes of multiple sites |
| 20080 | Other named variants of lymphosarcoma and reticulosarcoma - unspecified site - extranodal and solid organ sites |
| 20081 | Other named variants of lymphosarcoma and reticulosarcoma - lymph nodes of head - face - and neck |
| 20082 | Other named variants of lymphosarcoma and reticulosarcoma-intrathoracic lymph nodes |
| 20083 | Other named variants of lymphosarcoma and reticulosarcoma - intra-abdominal lymph nodes |
| 20084 | Other named variants of lymphosarcoma and reticulosarcoma - lymph nodes of axilla and upper limb |
| 20085 | Other named variants of lymphosarcoma and reticulosarcoma - lymph nodes of inguinal region and lower limb |
| 20086 | Other named variants of lymphosarcoma and reticulosarcoma - intrapelvic lymph nodes |
| 20087 | Other named variants of lymphosarcoma and reticulosarcoma - spleen |
| 20088 | Other named variants of lymphosarcoma and reticulosarcoma - lymph nodes of multiple sites |
| 20100 | Hodgkin's paragranuloma - unspecified site - extranodal and solid organ sites |
| 20101 | Hodgkin's paragranuloma - lymph nodes of head - face - and neck |
| 20102 | Hodgkin's paragranuloma - intrathoracic lymph nodes |
| 20103 | Hodgkin's paragranuloma - intra-abdominal lymph nodes |
| 20104 | Hodgkin's paragranuloma - lymph nodes of axilla and upper limb |
| 20105 | Hodgkin's paragranuloma - lymph nodes of inguinal region and lower limb |
| 20106 | Hodgkin's paragranuloma - intrapelvic lymph nodes |
| 20107 | Hodgkin's paragranuloma - spleen |
| 20108 | Hodgkin's paragranuloma - lymph nodes of multiple sites |
| 20110 | Hodgkin's granuloma - unspecified site - extranodal and solid organ sites |
| 20111 | Hodgkin's granuloma - lymph nodes of head - face - and neck |
| 20112 | Hodgkin's granuloma - intrathoracic lymph nodes |
| 20113 | Hodgkin's granuloma - intra-abdominal lymph nodes |
| 20114 | Hodgkin's granuloma - lymph nodes of axilla and upper limb |
| 20115 | Hodgkin's granuloma - lymph nodes of inguinal region and lower limb |
| 20116 | Hodgkin's granuloma - intrapelvic lymph nodes |
| 20117 | Hodgkin's granuloma - spleen |
| 20118 | Hodgkin's granuloma - lymph nodes of multiple sites |
| 20120 | Hodgkin's sarcoma - unspecified site - extranodal and solid organ sites |
| 20121 | Hodgkin's sarcoma - lymph nodes of head - face - and neck |
| 20122 | Hodgkin's sarcoma - intrathoracic lymph nodes |
| 20123 | Hodgkin's sarcoma - intra-abdominal lymph nodes |
| 20124 | Hodgkin's sarcoma - lymph nodes of axilla and upper limb |
| 20125 | Hodgkin's sarcoma - lymph nodes of inguinal region and lower limb |
| 20126 | Hodgkin's sarcoma - intrapelvic lymph nodes |
| 20127 | Hodgkin's sarcoma - spleen |
| 20128 | Hodgkin's sarcoma - lymph nodes of multiple sites |
| 20140 | Hodgkin's disease - lymphocytic-histiocytic predominance - unspecified site - extranodal and solid organ sites |
| 20141 | Hodgkin's disease - lymphocytic-histiocytic predominance - lymph nodes of head - face - and neck |
| 20142 | Hodgkin's disease - lymphocytic-histiocytic predominance - intrathoracic lymph nodes |
| 20143 | Hodgkin's disease - lymphocytic-histiocytic predominance - intra-abdominal lymph nodes |
| 20144 | Hodgkin's disease - lymphocytic-histiocytic predominance - lymph nodes of axilla and upper limb |
| 20145 | Hodgkin's disease - lymphocytic-histiocytic predominance - lymph nodes of inguinal region and lower limb |
| 20146 | Hodgkin's disease - lymphocytic-histiocytic predominance - intrapelvic lymph nodes |
| 20147 | Hodgkin's disease - lymphocytic-histiocytic predominance - spleen |
| 20148 | Hodgkin's disease - lymphocytic-histiocytic predominance - lymph nodes of multiple sites |
| 20150 | Hodgkin's disease - nodular sclerosis - unspecified site - extranodal and solid organ sites |
| 20151 | Hodgkin's disease - nodular sclerosis - lymph nodes of head - face - and neck |
| 20152 | Hodgkin's disease - nodular sclerosis - intrathoracic lymph nodes |
| 20153 | Hodgkin's disease - nodular sclerosis - intra-abdominal lymph nodes |
| 20154 | Hodgkin's disease - nodular sclerosis - lymph nodes of axilla and upper limb |
| 20155 | Hodgkin's disease - nodular sclerosis - lymph nodes of inguinal region and lower limb |
| 20156 | Hodgkin's disease - nodular sclerosis - intrapelvic lymph nodes |
| 20157 | Hodgkin's disease - nodular sclerosis - spleen |
| 20158 | Hodgkin's disease - nodular sclerosis - lymph nodes of multiple sites |
| 20160 | Hodgkin's disease - mixed cellularity - unspecified site - extranodal and solid organ sites |
| 20161 | Hodgkin's disease - mixed cellularity - lymph nodes of head - face - and neck |
| 20162 | Hodgkin's disease - mixed cellularity - intrathoracic lymph nodes |
| 20163 | Hodgkin's disease - mixed cellularity - intra-abdominal lymph nodes |
| 20164 | Hodgkin's disease - mixed cellularity - lymph nodes of axilla and upper limb |
| 20165 | Hodgkin's disease - mixed cellularity - lymph nodes of inguinal region and lower limb |
| 20166 | Hodgkin's disease - mixed cellularity - intrapelvic lymph nodes |
| 20167 | Hodgkin's disease - mixed cellularity - spleen |
| 20168 | Hodgkin's disease - mixed cellularity - lymph nodes of multiple sites |
| 20170 | Hodgkin's disease - lymphocytic depletion - unspecified site - extranodal and solid organ sites |
| 20171 | Hodgkin's disease - lymphocytic depletion - lymph nodes of head - face - and neck |
| 20172 | Hodgkin's disease - lymphocytic depletion - intrathoracic lymph nodes |
| 20173 | Hodgkin's disease - lymphocytic depletion - intra-abdominal lymph nodes |
| 20174 | Hodgkin's disease - lymphocytic depletion - lymph nodes of axilla and upper limb |
| 20175 | Hodgkin's disease - lymphocytic depletion - lymph nodes of inguinal region and lower limb |
| 20176 | Hodgkin's disease - lymphocytic depletion - intrapelvic lymph nodes |
| 20177 | Hodgkin's disease - lymphocytic depletion - spleen |
| 20178 | Hodgkin's disease - lymphocytic depletion - lymph nodes of multiple sites |
| 20190 | Hodgkin's disease - unspecified type - unspecified site - extranodal and solid organ sites |
| 20191 | Hodgkin's disease - unspecified type - lymph nodes of head - face - and neck |
| 20192 | Hodgkin's disease - unspecified type - intrathoracic lymph nodes |
| 20193 | Hodgkin's disease - unspecified type - intra-abdominal lymph nodes |
| 20194 | Hodgkin's disease - unspecified type - lymph nodes of axilla and upper limb |
| 20195 | Hodgkin's disease - unspecified type - lymph nodes of inguinal region and lower limb |
| 20196 | Hodgkin's disease - unspecified type - intrapelvic lymph nodes |
| 20197 | Hodgkin's disease - unspecified type - spleen |
| 20198 | Hodgkin's disease - unspecified type - lymph nodes of multiple sites |
| 20200 | Nodular lymphoma - unspecified site - extranodal and solid organ sites |
| 20201 | Nodular lymphoma - lymph nodes of head - face - and neck |
| 20202 | Nodular lymphoma - intrathoracic lymph nodes |
| 20203 | Nodular lymphoma - intra-abdominal lymph nodes |
| 20204 | Nodular lymphoma - lymph nodes of axilla and upper limb |
| 20205 | Nodular lymphoma - lymph nodes of inguinal region and lower limb |
| 20206 | Nodular lymphoma - intrapelvic lymph nodes |
| 20207 | Nodular lymphoma - spleen |
| 20208 | Nodular lymphoma - lymph nodes of multiple sites |
| 20210 | Mycosis fungoides - unspecified site - extranodal and solid organ sites |
| 20211 | Mycosis fungoides - lymph nodes of head - face - and neck |
| 20212 | Mycosis fungoides - intrathoracic lymph nodes |
| 20213 | Mycosis fungoides - intra-abdominal lymph nodes |
| 20214 | Mycosis fungoides - lymph nodes of axilla and upper limb |
| 20215 | Mycosis fungoides - lymph nodes of inguinal region and lower limb |
| 20216 | Mycosis fungoides - intrapelvic lymph nodes |
| 20217 | Mycosis fungoides - spleen |
| 20218 | Mycosis fungoides - lymph nodes of multiple sites |
| 20220 | Sezary's disease - unspecified site - extranodal and solid organ sites |
| 20221 | Sezary's disease - lymph nodes of head - face - and neck |
| 20222 | Sezary's disease - intrathoracic lymph nodes |
| 20223 | Sezary's disease - intra-abdominal lymph nodes |
| 20224 | Sezary's disease - lymph nodes of axilla and upper limb |
| 20225 | Sezary's disease - lymph nodes of inguinal region and lower limb |
| 20226 | Sezary's disease - intrapelvic lymph nodes |
| 20227 | Sezary's disease - spleen |
| 20228 | Sezary's disease - lymph nodes of multiple sites |
| 20230 | Malignant histiocytosis - unspecified site - extranodal and solid organ sites |
| 20231 | Malignant histiocytosis - lymph nodes of head - face - and neck |
| 20232 | Malignant histiocytosis - intrathoracic lymph nodes |
| 20233 | Malignant histiocytosis - intra-abdominal lymph nodes |
| 20234 | Malignant histiocytosis - lymph nodes of axilla and upper limb |
| 20235 | Malignant histiocytosis - lymph nodes of inguinal region and lower limb |
| 20236 | Malignant histiocytosis - intrapelvic lymph nodes |
| 20237 | Malignant histiocytosis - spleen |
| 20238 | Malignant histiocytosis - lymph nodes of multiple sites |
| 20240 | Leukemic reticuloendotheliosis - unspecified site - extranodal and solid organ sites |
| 20241 | Leukemic reticuloendotheliosis - lymph nodes of head - face - and neck |
| 20242 | Leukemic reticuloendotheliosis - intrathoracic lymph nodes |
| 20243 | Leukemic reticuloendotheliosis - intra-abdominal lymph nodes |
| 20244 | Leukemic reticuloendotheliosis - lymph nodes of axilla and upper arm |
| 20245 | Leukemic reticuloendotheliosis - lymph nodes of inguinal region and lower limb |
| 20246 | Leukemic reticuloendotheliosis - intrapelvic lymph nodes |
| 20247 | Leukemic reticuloendotheliosis - spleen |
| 20248 | Leukemic reticuloendotheliosis - lymph nodes of multipes sites |
| 20250 | Letterer-siwe disease - unspecified site - extranodal and solid organ sites |
| 20251 | Letterer-siwe disease - lymph nodes of head - face - and neck |
| 20252 | Letterer-siwe disease - intrathoracic lymph nodes |
| 20253 | Letterer-siwe disease - intra-abdominal lymph nodes |
| 20254 | Letterer-siwe disease - lymph nodes of axilla and upper limb |
| 20255 | Letterer-siwe disease - lymph nodes of inguinal region and lower limb |
| 20256 | Letterer-siwe disease - intrapelvic lymph nodes |
| 20257 | Letterer-siwe disease - spleen |
| 20258 | Letterer-siwe disease - lymph nodes of multiple sites |
| 20260 | Malignant mast cell tumors - unspecified site - extranodal and solid organ sites |
| 20261 | Malignant mast cell tumors - lymph nodes of head - face - and neck |
| 20262 | Malignant mast cell tumors - intrathoracic lymph nodes |
| 20263 | Malignant mast cell tumors - intra-abdominal lymph nodes |
| 20264 | Malignant mast cell tumors - lymph nodes of axilla and upper limb |
| 20265 | Malignant mast cell tumors - lymph nodes of inguinal region and lower limb |
| 20266 | Malignant mast cell tumors - intrapelvic lymph nodes |
| 20267 | Malignant mast cell tumors - spleen |
| 20268 | Malignant mast cell tumors - lymph nodes of multiple sites |
| 20270 | Peripheral T cell lymphoma - unspecified site - extranodal and solid organ sites |
| 20271 | Peripheral T cell lymphoma - lymph nodes of head - face - and neck |
| 20272 | Peripheral T cell lymphoma - intrathoracic lymph nodes |
| 20273 | Peripheral T cell lymphoma - intra-abdominal lymph nodes |
| 20274 | Peripheral T cell lymphoma - lymph nodes of axilla and upper limb |
| 20275 | Peripheral T cell lymphoma - lymph nodes of inguinal region and lower limb |
| 20276 | Peripheral T cell lymphoma - intrapelvic lymph nodes |
| 20277 | Peripheral T cell lymphoma - spleen |
| 20278 | Peripheral T cell lymphoma - lymph nodes of multiple sites |
| 20280 | Other malignant lymphomas - unspecified site - extranodal and solid organ sites |
| 20281 | Other malignant lymphomas - lymph nodes of head - face - and neck |
| 20282 | Other malignant lymphomas - intrathoracic lymph nodes |
| 20283 | Other malignant lymphomas - intra-abdominal lymph nodes |
| 20284 | Other malignant lymphomas - lymph nodes of axilla and upper limb |
| 20285 | Other malignant lymphomas - lymph nodes of inguinal region and lower limb |
| 20286 | Other malignant lymphomas - intrapelvic lymph nodes |
| 20287 | Other malignant lymphomas - spleen |
| 20288 | Other malignant lymphomas - lymph nodes of multiple sites |
| 20290 | Other and unspecified malignant neoplasms of lymphoid and histiocytic tissue - unspecified site - extranodal and solid organ sites |
| 20291 | Other and unspecified malignant neoplasms of lymphoid and histiocytic tissue - lymph nodes of head - face - and neck |
| 20292 | Other and unspecified malignant neoplasms of lymphoid and histiocytic tissue - intrathoracic lymph nodes |
| 20293 | Other and unspecified malignant neoplasms of lymphoid and histiocytic tissue - intra-abdominal lymph nodes |
| 20294 | Other and unspecified malignant neoplasms of lymphoid and histiocytic tissue - lymph nodes of axilla and upper limb |
| 20295 | Other and unspecified malignant neoplasms of lymphoid and histiocytic tissue - lymph nodes of inguinal region and lower limb |
| 20296 | Other and unspecified malignant neoplasms of lymphoid and histiocytic tissue - intrapelvic lymph nodes |
| 20297 | Other and unspecified malignant neoplasms of lymphoid and histiocytic tissue - spleen |
| 20298 | Other and unspecified malignant neoplasms of lymphoid and histiocytic tissue - lymph nodes of multiple sites |
| 20300 | Multiple myeloma - without mention of having achieved remission |
| 20301 | Multiple myeloma - in remission |
| 20302 | Multiple myeloma - in relapse |
| 20310 | Plasma cell leukemia - without mention of having achieved remission |
| 20311 | Plasma cell leukemia - in remission |
| 20312 | Plasma cell leukemia - in relapse |
| 20380 | Other immunoproliferative neoplasms - without mention of having achieved remission |
| 20381 | Other immunoproliferative neoplasms - in remission |
| 20382 | Other immunoproliferative neoplasms - in relapse |
| 20400 | Acute lymphoid leukemia - without mention of having achieved remission |
| 20401 | Acute lymphoid leukemia - in remission |
| 20402 | Acute lymphoid leukemia - in relapse |
| 20410 | Chronic lymphoid leukemia - without mention of having achieved remission |
| 20411 | Chronic lymphoid leukemia - in remission |
| 20412 | Chronic lymphoid leukemia - in relapse |
| 20420 | Subacute lymphoid leukemia - without mention of having achieved remission |
| 20421 | Subacute lymphoid leukemia - in remission |
| 20422 | Subacute lymphoid leukemia - in relapse |
| 20480 | Other lymphoid leukemia - without mention of having achieved remission |
| 20481 | Other lymphoid leukemia - in remission |
| 20482 | Other lymphoid leukemia - in relapse |
| 20490 | Unspecified lymphoid leukemia - without mention of having achieved remission |
| 20491 | Unspecified lymphoid leukemia - in remission |
| 20492 | Unspecified lymphoid leukemia - in relapse |
| 20500 | Acute myeloid leukemia - without mention of having achieved remission |
| 20501 | Acute myeloid leukemia - in remission |
| 20502 | Acute myeloid leukemia - in relapse |
| 20510 | Chronic myeloid leukemia - without mention of having achieved remission |
| 20511 | Chronic myeloid leukemia - in remission |
| 20512 | Chronic myeloid leukemia - in relapse |
| 20520 | Subacute myeloid leukemia - without mention of having achieved remission |
| 20521 | Subacute myeloid leukemia-in remission |
| 20522 | Subacute myeloid leukemia - in relapse |
| 20530 | Myeloid sarcoma - without mention of having achieved remission |
| 20531 | Myeloid sarcoma - in remission |
| 20532 | Myeloid sarcoma - in relapse |
| 20580 | Other myeloid leukemia - without mention of having achieved remission |
| 20581 | Other myeloid leukemia - in remission |
| 20582 | Other myeloid leukemia - in relapse |
| 20590 | Unspecified myeloid leukemia - without mention of having achieved remission |
| 20591 | Unspecified myeloid leukemia - in remission |
| 20592 | Unspecified myeloid leukemia - in relapse |
| 20600 | Acute monocytic leukemia - without mention of having achieved remission |
| 20601 | Acute monocytic leukemia-in remission |
| 20602 | Acute monocytic leukemia - in relapse |
| 20610 | Chronic monocytic leukemia - without mention of having achieved remission |
| 20611 | Chronic monocytic leukemia - in remission |
| 20612 | Chronic monocytic leukemia - in relapse |
| 20620 | Subacute monocytic leukemia - without mention of having achieved remission |
| 20621 | Subacute monocytic leukemia - in remission |
| 20622 | Subacute monocytic leukemia - in relapse |
| 20680 | Other monocytic leukemia - without mention of having achieved remission |
| 20681 | Other monocytic leukemia - in remission |
| 20682 | Other monocytic leukemia - in relapse |
| 20690 | Unspecified monocytic leukemia - without mention of having achieved remission |
| 20691 | Unspecified monocytic leukemia - in remission |
| 20692 | Unspecified monocytic leukemia - in relapse |
| 20700 | Acute erythremia and erythroleukemia - without mention of having achieved remission |
| 20701 | Acute erythremia and erythroleukemia - in remission |
| 20702 | Acute erythremia and erythroleukemia - in relapse |
| 20710 | Chronic erythremia - without mention of having achieved remission |
| 20711 | Chronic erythremia - in remission |
| 20712 | Chronic erythremia - in relapse |
| 20720 | Megakaryocytic leukemia - without mention of having achieved remission |
| 20721 | Megakaryocytic leukemia - in remission |
| 20722 | Megakaryocytic leukemia - in relapse |
| 20780 | Other specified leukemia - without mention of having achieved remission |
| 20781 | Other specified leukemia - in remission |
| 20782 | Other specified leukemia - in relapse |
| 20800 | Acute leukemia of unspecified cell type - without mention of having achieved remission |
| 20801 | Acute leukemia of unspecified cell type - in remission |
| 20802 | Acute leukemia of unspecified cell type - in relapse |
| 20810 | Chronic leukemia of unspecified cell type - without mention of having achieved remission |
| 20811 | Chronic leukemia of unspecified cell type - in remission |
| 20812 | Chronic leukemia of unspecified cell type - in relapse |
| 20820 | Subacute leukemia of unspecified cell type - without mention of having achieved remission |
| 20821 | Subacute leukemia of unspecified cell type - in remission |
| 20822 | Subacute leukemia of unspecified cell type - in relapse |
| 20880 | Other leukemia of unspecified cell type - without mention of having achieved remission |
| 20881 | Other leukemia of unspecified cell type - in remission |
| 20882 | Other leukemia of unspecified cell type - in relapse |
| 20890 | Unspecified leukemia - without mention of having achieved remission |
| 20891 | Unspecified leukemia - in remission |
| 20892 | Unspecified leukemia - in relapse |
| 20900 | Malignant carcinoid tumor of the small intestine - unspecified portion |
| 20901 | Malignant carcinoid tumor of the duodenum |
| 20902 | Malignant carcinoid tumor of the jejunum |
| 20903 | Malignant carcinoid tumor of the ileum |
| 20910 | Malignant carcinoid tumor of the large intestine - unspecified portion |
| 20911 | Malignant carcinoid tumor of the appendix |
| 20912 | Malignant carcinoid tumor of the cecum |
| 20913 | Malignant carcinoid tumor of the ascending colon |
| 20914 | Malignant carcinoid tumor of the transverse colon |
| 20915 | Malignant carcinoid tumor of the descending colon |
| 20916 | Malignant carcinoid tumor of the sigmoid colon |
| 20917 | Malignant carcinoid tumor of the rectum |
| 20920 | Malignant carcinoid tumor of unknown primary site |
| 20921 | Malignant carcinoid tumor of the bronchus and lung |
| 20922 | Malignant carcinoid tumor of the thymus |
| 20923 | Malignant carcinoid tumor of the stomach |
| 20924 | Malignant carcinoid tumor of the kidney |
| 20925 | Malignant carcinoid tumor of foregut - not otherwise specified |
| 20926 | Malignant carcinoid tumor of midgut - not otherwise specified |
| 20927 | Malignant carcinoid tumor of hindgut - not otherwise specified |
| 20929 | Malignant carcinoid tumor of other sites |
| 20930 | Malignant poorly differentiated neuroendocrine carcinoma - any site |
| 20931 | Merkel cell carcinoma of the face |
| 20932 | Merkel cell carcinoma of the scalp and neck |
| 20933 | Merkel cell carcinoma of the upper limb |
| 20934 | Merkel cell carcinoma of the lower limb |
| 20935 | Merkel cell carcinoma of the trunk |
| 20936 | Merkel cell carcinoma of other sites |
| 20940 | Benign carcinoid tumor of the small intestine - unspecified portion |
| 20941 | Benign carcinoid tumor of the duodenum |
| 20942 | Benign carcinoid tumor of the jejunum |
| 20943 | Benign carcinoid tumor of the ileum |
| 20950 | Benign carcinoid tumor of the large intestine - unspecified portion |
| 20951 | Benign carcinoid tumor of the appendix |
| 20952 | Benign carcinoid tumor of the cecum |
| 20953 | Benign carcinoid tumor of the ascending colon |
| 20954 | Benign carcinoid tumor of the transverse colon |
| 20955 | Benign carcinoid tumor of the descending colon |
| 20956 | Benign carcinoid tumor of the sigmoid colon |
| 20957 | Benign carcinoid tumor of the rectum |
| 20960 | Benign carcinoid tumor of unknown primary site |
| 20961 | Benign carcinoid tumor of the bronchus and lung |
| 20962 | Benign carcinoid tumor of the thymus |
| 20963 | Benign carcinoid tumor of the stomach |
| 20964 | Benign carcinoid tumor of the kidney |
| 20965 | Benign carcinoid tumor of foregut - not otherwise specified |
| 20966 | Benign carcinoid tumor of midgut - not otherwise specified |
| 20967 | Benign carcinoid tumor of hindgut - not otherwise specified |
| 20969 | Benign carcinoid tumor of other sites |
| 20970 | Secondary neuroendocrine tumor - unspecified site |
| 20971 | Secondary neuroendocrine tumor of distant lymph nodes |
| 20972 | Secondary neuroendocrine tumor of liver |
| 20973 | Secondary neuroendocrine tumor of bone |
| 20974 | Secondary neuroendocrine tumor of peritoneum |
| 20975 | Secondary Merkel cell carcinoma |
| 20979 | Secondary neuroendocrine tumor of other sites |
| 22381 | Benign neoplasm of urethra |
| 22389 | Benign neoplasm of other specified sites of urinary organs |
| 22800 | Hemangioma of unspecified site |
| 22801 | Hemangioma of skin and subcutaneous tissue |
| 22802 | Hemangioma of intracranial structures |
| 22803 | Hemangioma of retina |
| 22804 | Hemangioma of intra-abdominal structures |
| 22809 | Hemangioma of other sites |
| 23330 | Carcinoma in situ - unspecified female genital organ |
| 23331 | Carcinoma in situ - vagina |
| 23332 | Carcinoma in situ - vulva |
| 23339 | Carcinoma in situ - other female genital organ |
| 23690 | Neoplasm of uncertain behavior of urinary organ - unspecified |
| 23691 | Neoplasm of uncertain behavior of kidney and ureter |
| 23699 | Neoplasm of uncertain behavior of other and unspecified urinary organs |
| 23770 | Neurofibromatosis - unspecified |
| 23771 | Neurofibromatosis - type 1 [von recklinghausen's disease] |
| 23772 | Neurofibromatosis - type 2 [acoustic neurofibromatosis] |
| 23773 | Schwannomatosis |
| 23779 | Other neurofibromatosis |
| 23871 | Essential thrombocythemia |
| 23872 | Low grade myelodysplastic syndrome lesions |
| 23873 | High grade myelodysplastic syndrome lesions |
| 23874 | Myelodysplastic syndrome with 5q deletion |
| 23875 | Myelodysplastic syndrome - unspecified |
| 23876 | Myelofibrosis with myeloid metaplasia |
| 23877 | Post-transplant lymphoproliferative disorder (PTLD) |
| 23879 | Other lymphatic and hematopoietic tissues |
| 23981 | Neoplasms of unspecified nature - retina and choroid |
| 23989 | Neoplasms of unspecified nature - other specified sites |
| **Cancer-related ICD-10 Codes** | |
| C000 | Malignant neoplasm of external upper lip |
| C001 | Malignant neoplasm of external lower lip |
| C002 | Malignant neoplasm of external lip, unspecified |
| C003 | Malignant neoplasm of upper lip, inner aspect |
| C004 | Malignant neoplasm of lower lip, inner aspect |
| C005 | Malignant neoplasm of lip, unspecified, inner aspect |
| C006 | Malignant neoplasm of commissure of lip, unspecified |
| C008 | Malignant neoplasm of overlapping sites of lip |
| C009 | Malignant neoplasm of lip, unspecified |
| C01 | Malignant neoplasm of base of tongue |
| C020 | Malignant neoplasm of dorsal surface of tongue |
| C021 | Malignant neoplasm of border of tongue |
| C022 | Malignant neoplasm of ventral surface of tongue |
| C023 | Malignant neoplasm of anterior two-thirds of tongue, part unspecified |
| C024 | Malignant neoplasm of lingual tonsil |
| C028 | Malignant neoplasm of overlapping sites of tongue |
| C029 | Malignant neoplasm of tongue, unspecified |
| C030 | Malignant neoplasm of upper gum |
| C031 | Malignant neoplasm of lower gum |
| C039 | Malignant neoplasm of gum, unspecified |
| C040 | Malignant neoplasm of anterior floor of mouth |
| C041 | Malignant neoplasm of lateral floor of mouth |
| C048 | Malignant neoplasm of overlapping sites of floor of mouth |
| C049 | Malignant neoplasm of floor of mouth, unspecified |
| C050 | Malignant neoplasm of hard palate |
| C051 | Malignant neoplasm of soft palate |
| C052 | Malignant neoplasm of uvula |
| C058 | Malignant neoplasm of overlapping sites of palate |
| C059 | Malignant neoplasm of palate, unspecified |
| C060 | Malignant neoplasm of cheek mucosa |
| C061 | Malignant neoplasm of vestibule of mouth |
| C062 | Malignant neoplasm of retromolar area |
| C0680 | Malignant neoplasm of overlapping sites of unspecified parts of mouth |
| C0689 | Malignant neoplasm of overlapping sites of other parts of mouth |
| C069 | Malignant neoplasm of mouth, unspecified |
| C07 | Malignant neoplasm of parotid gland |
| C080 | Malignant neoplasm of submandibular gland |
| C081 | Malignant neoplasm of sublingual gland |
| C089 | Malignant neoplasm of major salivary gland, unspecified |
| C090 | Malignant neoplasm of tonsillar fossa |
| C091 | Malignant neoplasm of tonsillar pillar (anterior) (posterior) |
| C098 | Malignant neoplasm of overlapping sites of tonsil |
| C099 | Malignant neoplasm of tonsil, unspecified |
| C100 | Malignant neoplasm of vallecula |
| C101 | Malignant neoplasm of anterior surface of epiglottis |
| C102 | Malignant neoplasm of lateral wall of oropharynx |
| C103 | Malignant neoplasm of posterior wall of oropharynx |
| C104 | Malignant neoplasm of branchial cleft |
| C108 | Malignant neoplasm of overlapping sites of oropharynx |
| C109 | Malignant neoplasm of oropharynx, unspecified |
| C110 | Malignant neoplasm of superior wall of nasopharynx |
| C111 | Malignant neoplasm of posterior wall of nasopharynx |
| C112 | Malignant neoplasm of lateral wall of nasopharynx |
| C113 | Malignant neoplasm of anterior wall of nasopharynx |
| C118 | Malignant neoplasm of overlapping sites of nasopharynx |
| C119 | Malignant neoplasm of nasopharynx, unspecified |
| C12 | Malignant neoplasm of pyriform sinus |
| C130 | Malignant neoplasm of postcricoid region |
| C131 | Malignant neoplasm of aryepiglottic fold, hypopharyngeal aspect |
| C132 | Malignant neoplasm of posterior wall of hypopharynx |
| C138 | Malignant neoplasm of overlapping sites of hypopharynx |
| C139 | Malignant neoplasm of hypopharynx, unspecified |
| C140 | Malignant neoplasm of pharynx, unspecified |
| C142 | Malignant neoplasm of Waldeyer's ring |
| C148 | Malignant neoplasm of overlapping sites of lip, oral cavity and pharynx |
| C153 | Malignant neoplasm of upper third of esophagus |
| C154 | Malignant neoplasm of middle third of esophagus |
| C155 | Malignant neoplasm of lower third of esophagus |
| C158 | Malignant neoplasm of overlapping sites of esophagus |
| C159 | Malignant neoplasm of esophagus, unspecified |
| C160 | Malignant neoplasm of cardia |
| C161 | Malignant neoplasm of fundus of stomach |
| C162 | Malignant neoplasm of body of stomach |
| C163 | Malignant neoplasm of pyloric antrum |
| C164 | Malignant neoplasm of pylorus |
| C165 | Malignant neoplasm of lesser curvature of stomach, unspecified |
| C166 | Malignant neoplasm of greater curvature of stomach, unspecified |
| C168 | Malignant neoplasm of overlapping sites of stomach |
| C169 | Malignant neoplasm of stomach, unspecified |
| C170 | Malignant neoplasm of duodenum |
| C171 | Malignant neoplasm of jejunum |
| C172 | Malignant neoplasm of ileum |
| C173 | Meckel's diverticulum, malignant |
| C178 | Malignant neoplasm of overlapping sites of small intestine |
| C179 | Malignant neoplasm of small intestine, unspecified |
| C180 | Malignant neoplasm of cecum |
| C181 | Malignant neoplasm of appendix |
| C182 | Malignant neoplasm of ascending colon |
| C183 | Malignant neoplasm of hepatic flexure |
| C184 | Malignant neoplasm of transverse colon |
| C185 | Malignant neoplasm of splenic flexure |
| C186 | Malignant neoplasm of descending colon |
| C187 | Malignant neoplasm of sigmoid colon |
| C188 | Malignant neoplasm of overlapping sites of colon |
| C189 | Malignant neoplasm of colon, unspecified |
| C19 | Malignant neoplasm of rectosigmoid junction |
| C20 | Malignant neoplasm of rectum |
| C210 | Malignant neoplasm of anus, unspecified |
| C211 | Malignant neoplasm of anal canal |
| C212 | Malignant neoplasm of cloacogenic zone |
| C218 | Malignant neoplasm of overlapping sites of rectum, anus and anal canal |
| C220 | Liver cell carcinoma |
| C221 | Intrahepatic bile duct carcinoma |
| C222 | Hepatoblastoma |
| C223 | Angiosarcoma of liver |
| C224 | Other sarcomas of liver |
| C227 | Other specified carcinomas of liver |
| C228 | Malignant neoplasm of liver, primary, unspecified as to type |
| C229 | Malignant neoplasm of liver, not specified as primary or secondary |
| C23 | Malignant neoplasm of gallbladder |
| C240 | Malignant neoplasm of extrahepatic bile duct |
| C241 | Malignant neoplasm of ampulla of Vater |
| C248 | Malignant neoplasm of overlapping sites of biliary tract |
| C249 | Malignant neoplasm of biliary tract, unspecified |
| C250 | Malignant neoplasm of head of pancreas |
| C251 | Malignant neoplasm of body of pancreas |
| C252 | Malignant neoplasm of tail of pancreas |
| C253 | Malignant neoplasm of pancreatic duct |
| C254 | Malignant neoplasm of endocrine pancreas |
| C257 | Malignant neoplasm of other parts of pancreas |
| C258 | Malignant neoplasm of overlapping sites of pancreas |
| C259 | Malignant neoplasm of pancreas, unspecified |
| C260 | Malignant neoplasm of intestinal tract, part unspecified |
| C261 | Malignant neoplasm of spleen |
| C269 | Malignant neoplasm of ill-defined sites within the digestive system |
| C300 | Malignant neoplasm of nasal cavity |
| C301 | Malignant neoplasm of middle ear |
| C310 | Malignant neoplasm of maxillary sinus |
| C311 | Malignant neoplasm of ethmoidal sinus |
| C312 | Malignant neoplasm of frontal sinus |
| C313 | Malignant neoplasm of sphenoid sinus |
| C318 | Malignant neoplasm of overlapping sites of accessory sinuses |
| C319 | Malignant neoplasm of accessory sinus, unspecified |
| C320 | Malignant neoplasm of glottis |
| C321 | Malignant neoplasm of supraglottis |
| C322 | Malignant neoplasm of subglottis |
| C323 | Malignant neoplasm of laryngeal cartilage |
| C328 | Malignant neoplasm of overlapping sites of larynx |
| C329 | Malignant neoplasm of larynx, unspecified |
| C33 | Malignant neoplasm of trachea |
| C3400 | Malignant neoplasm of unspecified main bronchus |
| C3401 | Malignant neoplasm of right main bronchus |
| C3402 | Malignant neoplasm of left main bronchus |
| C3410 | Malignant neoplasm of upper lobe, unspecified bronchus or lung |
| C3411 | Malignant neoplasm of upper lobe, right bronchus or lung |
| C3412 | Malignant neoplasm of upper lobe, left bronchus or lung |
| C342 | Malignant neoplasm of middle lobe, bronchus or lung |
| C3430 | Malignant neoplasm of lower lobe, unspecified bronchus or lung |
| C3431 | Malignant neoplasm of lower lobe, right bronchus or lung |
| C3432 | Malignant neoplasm of lower lobe, left bronchus or lung |
| C3480 | Malignant neoplasm of overlapping sites of unspecified bronchus and lung |
| C3481 | Malignant neoplasm of overlapping sites of right bronchus and lung |
| C3482 | Malignant neoplasm of overlapping sites of left bronchus and lung |
| C3490 | Malignant neoplasm of unspecified part of unspecified bronchus or lung |
| C3491 | Malignant neoplasm of unspecified part of right bronchus or lung |
| C3492 | Malignant neoplasm of unspecified part of left bronchus or lung |
| C37 | Malignant neoplasm of thymus |
| C380 | Malignant neoplasm of heart |
| C381 | Malignant neoplasm of anterior mediastinum |
| C382 | Malignant neoplasm of posterior mediastinum |
| C383 | Malignant neoplasm of mediastinum, part unspecified |
| C384 | Malignant neoplasm of pleura |
| C388 | Malignant neoplasm of overlapping sites of heart, mediastinum and pleura |
| C390 | Malignant neoplasm of upper respiratory tract, part unspecified |
| C399 | Malignant neoplasm of lower respiratory tract, part unspecified |
| C4000 | Malignant neoplasm of scapula and long bones of unspecified upper limb |
| C4001 | Malignant neoplasm of scapula and long bones of right upper limb |
| C4002 | Malignant neoplasm of scapula and long bones of left upper limb |
| C4010 | Malignant neoplasm of short bones of unspecified upper limb |
| C4011 | Malignant neoplasm of short bones of right upper limb |
| C4012 | Malignant neoplasm of short bones of left upper limb |
| C4020 | Malignant neoplasm of long bones of unspecified lower limb |
| C4021 | Malignant neoplasm of long bones of right lower limb |
| C4022 | Malignant neoplasm of long bones of left lower limb |
| C4030 | Malignant neoplasm of short bones of unspecified lower limb |
| C4031 | Malignant neoplasm of short bones of right lower limb |
| C4032 | Malignant neoplasm of short bones of left lower limb |
| C4080 | Malignant neoplasm of overlapping sites of bone and articular cartilage of unspecified limb |
| C4081 | Malignant neoplasm of overlapping sites of bone and articular cartilage of right limb |
| C4082 | Malignant neoplasm of overlapping sites of bone and articular cartilage of left limb |
| C4090 | Malignant neoplasm of unspecified bones and articular cartilage of unspecified limb |
| C4091 | Malignant neoplasm of unspecified bones and articular cartilage of right limb |
| C4092 | Malignant neoplasm of unspecified bones and articular cartilage of left limb |
| C410 | Malignant neoplasm of bones of skull and face |
| C411 | Malignant neoplasm of mandible |
| C412 | Malignant neoplasm of vertebral column |
| C413 | Malignant neoplasm of ribs, sternum and clavicle |
| C414 | Malignant neoplasm of pelvic bones, sacrum and coccyx |
| C419 | Malignant neoplasm of bone and articular cartilage, unspecified |
| C430 | Malignant melanoma of lip |
| C4310 | Malignant melanoma of unspecified eyelid, including canthus |
| C43111 | Malignant melanoma of right upper eyelid, including canthus |
| C43112 | Malignant melanoma of right lower eyelid, including canthus |
| C43121 | Malignant melanoma of left upper eyelid, including canthus |
| C43122 | Malignant melanoma of left lower eyelid, including canthus |
| C4320 | Malignant melanoma of unspecified ear and external auricular canal |
| C4321 | Malignant melanoma of right ear and external auricular canal |
| C4322 | Malignant melanoma of left ear and external auricular canal |
| C4330 | Malignant melanoma of unspecified part of face |
| C4331 | Malignant melanoma of nose |
| C4339 | Malignant melanoma of other parts of face |
| C434 | Malignant melanoma of scalp and neck |
| C4351 | Malignant melanoma of anal skin |
| C4352 | Malignant melanoma of skin of breast |
| C4359 | Malignant melanoma of other part of trunk |
| C4360 | Malignant melanoma of unspecified upper limb, including shoulder |
| C4361 | Malignant melanoma of right upper limb, including shoulder |
| C4362 | Malignant melanoma of left upper limb, including shoulder |
| C4370 | Malignant melanoma of unspecified lower limb, including hip |
| C4371 | Malignant melanoma of right lower limb, including hip |
| C4372 | Malignant melanoma of left lower limb, including hip |
| C438 | Malignant melanoma of overlapping sites of skin |
| C439 | Malignant melanoma of skin, unspecified |
| C4400 | Unspecified malignant neoplasm of skin of lip |
| C4401 | Basal cell carcinoma of skin of lip |
| C4402 | Squamous cell carcinoma of skin of lip |
| C4409 | Other specified malignant neoplasm of skin of lip |
| C44101 | Unspecified malignant neoplasm of skin of unspecified eyelid, including canthus |
| C44102 | 1 Unspecified malignant neoplasm of skin of right upper eyelid, including canthus |
| C44102 | 2 Unspecified malignant neoplasm of skin of right lower eyelid, including canthus |
| C44109 | 1 Unspecified malignant neoplasm of skin of left upper eyelid, including canthus |
| C44109 | 2 Unspecified malignant neoplasm of skin of left lower eyelid, including canthus |
| C44111 | Basal cell carcinoma of skin of unspecified eyelid, including canthus |
| C44112 | 1 Basal cell carcinoma of skin of right upper eyelid, including canthus |
| C44112 | 2 Basal cell carcinoma of skin of right lower eyelid, including canthus |
| C44119 | 1 Basal cell carcinoma of skin of left upper eyelid, including canthus |
| C44119 | 2 Basal cell carcinoma of skin of left lower eyelid, including canthus |
| C44121 | Squamous cell carcinoma of skin of unspecified eyelid, including canthus |
| C44122 | 1 Squamous cell carcinoma of skin of right upper eyelid, including canthus |
| C44122 | 2 Squamous cell carcinoma of skin of right lower eyelid, including canthus |
| C44129 | 1 Squamous cell carcinoma of skin of left upper eyelid, including canthus |
| C44129 | 2 Squamous cell carcinoma of skin of left lower eyelid, including canthus |
| C44131 | Sebaceous cell carcinoma of skin of unspecified eyelid, including canthus |
| C44132 | 1 Sebaceous cell carcinoma of skin of right upper eyelid, including canthus |
| C44132 | 2 Sebaceous cell carcinoma of skin of right lower eyelid, including canthus |
| C44139 | 1 Sebaceous cell carcinoma of skin of left upper eyelid, including canthus |
| C44139 | 2 Sebaceous cell carcinoma of skin of left lower eyelid, including canthus |
| C44191 | Other specified malignant neoplasm of skin of unspecified eyelid, including canthus |
| C44192 | 1 Other specified malignant neoplasm of skin of right upper eyelid, including canthus |
| C44192 | 2 Other specified malignant neoplasm of skin of right lower eyelid, including canthus |
| C44199 | 1 Other specified malignant neoplasm of skin of left upper eyelid, including canthus |
| C44199 | 2 Other specified malignant neoplasm of skin of left lower eyelid, including canthus |
| C44201 | Unspecified malignant neoplasm of skin of unspecified ear and external auricular canal |
| C44202 | Unspecified malignant neoplasm of skin of right ear and external auricular canal |
| C44209 | Unspecified malignant neoplasm of skin of left ear and external auricular canal |
| C44211 | Basal cell carcinoma of skin of unspecified ear and external auricular canal |
| C44212 | Basal cell carcinoma of skin of right ear and external auricular canal |
| C44219 | Basal cell carcinoma of skin of left ear and external auricular canal |
| C44221 | Squamous cell carcinoma of skin of unspecified ear and external auricular canal |
| C44222 | Squamous cell carcinoma of skin of right ear and external auricular canal |
| C44229 | Squamous cell carcinoma of skin of left ear and external auricular canal |
| C44291 | Other specified malignant neoplasm of skin of unspecified ear and external auricular canal |
| C44292 | Other specified malignant neoplasm of skin of right ear and external auricular canal |
| C44299 | Other specified malignant neoplasm of skin of left ear and external auricular canal |
| C44300 | Unspecified malignant neoplasm of skin of unspecified part of face |
| C44301 | Unspecified malignant neoplasm of skin of nose |
| C44309 | Unspecified malignant neoplasm of skin of other parts of face |
| C44310 | Basal cell carcinoma of skin of unspecified parts of face |
| C44311 | Basal cell carcinoma of skin of nose |
| C44319 | Basal cell carcinoma of skin of other parts of face |
| C44320 | Squamous cell carcinoma of skin of unspecified parts of face |
| C44321 | Squamous cell carcinoma of skin of nose |
| C44329 | Squamous cell carcinoma of skin of other parts of face |
| C44390 | Other specified malignant neoplasm of skin of unspecified parts of face |
| C44391 | Other specified malignant neoplasm of skin of nose |
| C44399 | Other specified malignant neoplasm of skin of other parts of face |
| C4440 | Unspecified malignant neoplasm of skin of scalp and neck |
| C4441 | Basal cell carcinoma of skin of scalp and neck |
| C4442 | Squamous cell carcinoma of skin of scalp and neck |
| C4449 | Other specified malignant neoplasm of skin of scalp and neck |
| C44500 | Unspecified malignant neoplasm of anal skin |
| C44501 | Unspecified malignant neoplasm of skin of breast |
| C44509 | Unspecified malignant neoplasm of skin of other part of trunk |
| C44510 | Basal cell carcinoma of anal skin |
| C44511 | Basal cell carcinoma of skin of breast |
| C44519 | Basal cell carcinoma of skin of other part of trunk |
| C44520 | Squamous cell carcinoma of anal skin |
| C44521 | Squamous cell carcinoma of skin of breast |
| C44529 | Squamous cell carcinoma of skin of other part of trunk |
| C44590 | Other specified malignant neoplasm of anal skin |
| C44591 | Other specified malignant neoplasm of skin of breast |
| C44599 | Other specified malignant neoplasm of skin of other part of trunk |
| C44601 | Unspecified malignant neoplasm of skin of unspecified upper limb, including shoulder |
| C44602 | Unspecified malignant neoplasm of skin of right upper limb, including shoulder |
| C44609 | Unspecified malignant neoplasm of skin of left upper limb, including shoulder |
| C44611 | Basal cell carcinoma of skin of unspecified upper limb, including shoulder |
| C44612 | Basal cell carcinoma of skin of right upper limb, including shoulder |
| C44619 | Basal cell carcinoma of skin of left upper limb, including shoulder |
| C44621 | Squamous cell carcinoma of skin of unspecified upper limb, including shoulder |
| C44622 | Squamous cell carcinoma of skin of right upper limb, including shoulder |
| C44629 | Squamous cell carcinoma of skin of left upper limb, including shoulder |
| C44691 | Other specified malignant neoplasm of skin of unspecified upper limb, including shoulder |
| C44692 | Other specified malignant neoplasm of skin of right upper limb, including shoulder |
| C44699 | Other specified malignant neoplasm of skin of left upper limb, including shoulder |
| C44701 | Unspecified malignant neoplasm of skin of unspecified lower limb, including hip |
| C44702 | Unspecified malignant neoplasm of skin of right lower limb, including hip |
| C44709 | Unspecified malignant neoplasm of skin of left lower limb, including hip |
| C44711 | Basal cell carcinoma of skin of unspecified lower limb, including hip |
| C44712 | Basal cell carcinoma of skin of right lower limb, including hip |
| C44719 | Basal cell carcinoma of skin of left lower limb, including hip |
| C44721 | Squamous cell carcinoma of skin of unspecified lower limb, including hip |
| C44722 | Squamous cell carcinoma of skin of right lower limb, including hip |
| C44729 | Squamous cell carcinoma of skin of left lower limb, including hip |
| C44791 | Other specified malignant neoplasm of skin of unspecified lower limb, including hip |
| C44792 | Other specified malignant neoplasm of skin of right lower limb, including hip |
| C44799 | Other specified malignant neoplasm of skin of left lower limb, including hip |
| C4480 | Unspecified malignant neoplasm of overlapping sites of skin |
| C4481 | Basal cell carcinoma of overlapping sites of skin |
| C4482 | Squamous cell carcinoma of overlapping sites of skin |
| C4489 | Other specified malignant neoplasm of overlapping sites of skin |
| C4490 | Unspecified malignant neoplasm of skin, unspecified |
| C4491 | Basal cell carcinoma of skin, unspecified |
| C4492 | Squamous cell carcinoma of skin, unspecified |
| C4499 | Other specified malignant neoplasm of skin, unspecified |
| C450 | Mesothelioma of pleura |
| C451 | Mesothelioma of peritoneum |
| C452 | Mesothelioma of pericardium |
| C457 | Mesothelioma of other sites |
| C459 | Mesothelioma, unspecified |
| C460 | Kaposi's sarcoma of skin |
| C461 | Kaposi's sarcoma of soft tissue |
| C462 | Kaposi's sarcoma of palate |
| C463 | Kaposi's sarcoma of lymph nodes |
| C464 | Kaposi's sarcoma of gastrointestinal sites |
| C4650 | Kaposi's sarcoma of unspecified lung |
| C4651 | Kaposi's sarcoma of right lung |
| C4652 | Kaposi's sarcoma of left lung |
| C467 | Kaposi's sarcoma of other sites |
| C469 | Kaposi's sarcoma, unspecified |
| C470 | Malignant neoplasm of peripheral nerves of head, face and neck |
| C4710 | Malignant neoplasm of peripheral nerves of unspecified upper limb, including shoulder |
| C4711 | Malignant neoplasm of peripheral nerves of right upper limb, including shoulder |
| C4712 | Malignant neoplasm of peripheral nerves of left upper limb, including shoulder |
| C4720 | Malignant neoplasm of peripheral nerves of unspecified lower limb, including hip |
| C4721 | Malignant neoplasm of peripheral nerves of right lower limb, including hip |
| C4722 | Malignant neoplasm of peripheral nerves of left lower limb, including hip |
| C473 | Malignant neoplasm of peripheral nerves of thorax |
| C474 | Malignant neoplasm of peripheral nerves of abdomen |
| C475 | Malignant neoplasm of peripheral nerves of pelvis |
| C476 | Malignant neoplasm of peripheral nerves of trunk, unspecified |
| C478 | Malignant neoplasm of overlapping sites of peripheral nerves and autonomic nervous system |
| C479 | Malignant neoplasm of peripheral nerves and autonomic nervous system, unspecified |
| C480 | Malignant neoplasm of retroperitoneum |
| C481 | Malignant neoplasm of specified parts of peritoneum |
| C482 | Malignant neoplasm of peritoneum, unspecified |
| C488 | Malignant neoplasm of overlapping sites of retroperitoneum and peritoneum |
| C490 | Malignant neoplasm of connective and soft tissue of head, face and neck |
| C4910 | Malignant neoplasm of connective and soft tissue of unspecified upper limb, including shoulder |
| C4911 | Malignant neoplasm of connective and soft tissue of right upper limb, including shoulder |
| C4912 | Malignant neoplasm of connective and soft tissue of left upper limb, including shoulder |
| C4920 | Malignant neoplasm of connective and soft tissue of unspecified lower limb, including hip |
| C4921 | Malignant neoplasm of connective and soft tissue of right lower limb, including hip |
| C4922 | Malignant neoplasm of connective and soft tissue of left lower limb, including hip |
| C493 | Malignant neoplasm of connective and soft tissue of thorax |
| C494 | Malignant neoplasm of connective and soft tissue of abdomen |
| C495 | Malignant neoplasm of connective and soft tissue of pelvis |
| C496 | Malignant neoplasm of connective and soft tissue of trunk, unspecified |
| C498 | Malignant neoplasm of overlapping sites of connective and soft tissue |
| C499 | Malignant neoplasm of connective and soft tissue, unspecified |
| C49A0 | Gastrointestinal stromal tumor, unspecified site |
| C49A1 | Gastrointestinal stromal tumor of esophagus |
| C49A2 | Gastrointestinal stromal tumor of stomach |
| C49A3 | Gastrointestinal stromal tumor of small intestine |
| C49A4 | Gastrointestinal stromal tumor of large intestine |
| C49A5 | Gastrointestinal stromal tumor of rectum |
| C49A9 | Gastrointestinal stromal tumor of other sites |
| C4A0 | Merkel cell carcinoma of lip |
| C4A10 | Merkel cell carcinoma of unspecified eyelid, including canthus |
| C4A111 | Merkel cell carcinoma of right upper eyelid, including canthus |
| C4A112 | Merkel cell carcinoma of right lower eyelid, including canthus |
| C4A121 | Merkel cell carcinoma of left upper eyelid, including canthus |
| C4A122 | Merkel cell carcinoma of left lower eyelid, including canthus |
| C4A20 | Merkel cell carcinoma of unspecified ear and external auricular canal |
| C4A21 | Merkel cell carcinoma of right ear and external auricular canal |
| C4A22 | Merkel cell carcinoma of left ear and external auricular canal |
| C4A30 | Merkel cell carcinoma of unspecified part of face |
| C4A31 | Merkel cell carcinoma of nose |
| C4A39 | Merkel cell carcinoma of other parts of face |
| C4A4 | Merkel cell carcinoma of scalp and neck |
| C4A51 | Merkel cell carcinoma of anal skin |
| C4A52 | Merkel cell carcinoma of skin of breast |
| C4A59 | Merkel cell carcinoma of other part of trunk |
| C4A60 | Merkel cell carcinoma of unspecified upper limb, including shoulder |
| C4A61 | Merkel cell carcinoma of right upper limb, including shoulder |
| C4A62 | Merkel cell carcinoma of left upper limb, including shoulder |
| C4A70 | Merkel cell carcinoma of unspecified lower limb, including hip |
| C4A71 | Merkel cell carcinoma of right lower limb, including hip |
| C4A72 | Merkel cell carcinoma of left lower limb, including hip |
| C4A8 | Merkel cell carcinoma of overlapping sites |
| C4A9 | Merkel cell carcinoma, unspecified |
| C50011 | Malignant neoplasm of nipple and areola, right female breast |
| C50012 | Malignant neoplasm of nipple and areola, left female breast |
| C50019 | Malignant neoplasm of nipple and areola, unspecified female breast |
| C50021 | Malignant neoplasm of nipple and areola, right male breast |
| C50022 | Malignant neoplasm of nipple and areola, left male breast |
| C50029 | Malignant neoplasm of nipple and areola, unspecified male breast |
| C50111 | Malignant neoplasm of central portion of right female breast |
| C50112 | Malignant neoplasm of central portion of left female breast |
| C50119 | Malignant neoplasm of central portion of unspecified female breast |
| C50121 | Malignant neoplasm of central portion of right male breast |
| C50122 | Malignant neoplasm of central portion of left male breast |
| C50129 | Malignant neoplasm of central portion of unspecified male breast |
| C50211 | Malignant neoplasm of upper-inner quadrant of right female breast |
| C50212 | Malignant neoplasm of upper-inner quadrant of left female breast |
| C50219 | Malignant neoplasm of upper-inner quadrant of unspecified female breast |
| C50221 | Malignant neoplasm of upper-inner quadrant of right male breast |
| C50222 | Malignant neoplasm of upper-inner quadrant of left male breast |
| C50229 | Malignant neoplasm of upper-inner quadrant of unspecified male breast |
| C50311 | Malignant neoplasm of lower-inner quadrant of right female breast |
| C50312 | Malignant neoplasm of lower-inner quadrant of left female breast |
| C50319 | Malignant neoplasm of lower-inner quadrant of unspecified female breast |
| C50321 | Malignant neoplasm of lower-inner quadrant of right male breast |
| C50322 | Malignant neoplasm of lower-inner quadrant of left male breast |
| C50329 | Malignant neoplasm of lower-inner quadrant of unspecified male breast |
| C50411 | Malignant neoplasm of upper-outer quadrant of right female breast |
| C50412 | Malignant neoplasm of upper-outer quadrant of left female breast |
| C50419 | Malignant neoplasm of upper-outer quadrant of unspecified female breast |
| C50421 | Malignant neoplasm of upper-outer quadrant of right male breast |
| C50422 | Malignant neoplasm of upper-outer quadrant of left male breast |
| C50429 | Malignant neoplasm of upper-outer quadrant of unspecified male breast |
| C50511 | Malignant neoplasm of lower-outer quadrant of right female breast |
| C50512 | Malignant neoplasm of lower-outer quadrant of left female breast |
| C50519 | Malignant neoplasm of lower-outer quadrant of unspecified female breast |
| C50521 | Malignant neoplasm of lower-outer quadrant of right male breast |
| C50522 | Malignant neoplasm of lower-outer quadrant of left male breast |
| C50529 | Malignant neoplasm of lower-outer quadrant of unspecified male breast |
| C50611 | Malignant neoplasm of axillary tail of right female breast |
| C50612 | Malignant neoplasm of axillary tail of left female breast |
| C50619 | Malignant neoplasm of axillary tail of unspecified female breast |
| C50621 | Malignant neoplasm of axillary tail of right male breast |
| C50622 | Malignant neoplasm of axillary tail of left male breast |
| C50629 | Malignant neoplasm of axillary tail of unspecified male breast |
| C50811 | Malignant neoplasm of overlapping sites of right female breast |
| C50812 | Malignant neoplasm of overlapping sites of left female breast |
| C50819 | Malignant neoplasm of overlapping sites of unspecified female breast |
| C50821 | Malignant neoplasm of overlapping sites of right male breast |
| C50822 | Malignant neoplasm of overlapping sites of left male breast |
| C50829 | Malignant neoplasm of overlapping sites of unspecified male breast |
| C50911 | Malignant neoplasm of unspecified site of right female breast |
| C50912 | Malignant neoplasm of unspecified site of left female breast |
| C50919 | Malignant neoplasm of unspecified site of unspecified female breast |
| C50921 | Malignant neoplasm of unspecified site of right male breast |
| C50922 | Malignant neoplasm of unspecified site of left male breast |
| C50929 | Malignant neoplasm of unspecified site of unspecified male breast |
| C510 | Malignant neoplasm of labium majus |
| C511 | Malignant neoplasm of labium minus |
| C512 | Malignant neoplasm of clitoris |
| C518 | Malignant neoplasm of overlapping sites of vulva |
| C519 | Malignant neoplasm of vulva, unspecified |
| C52 | Malignant neoplasm of vagina |
| C530 | Malignant neoplasm of endocervix |
| C531 | Malignant neoplasm of exocervix |
| C538 | Malignant neoplasm of overlapping sites of cervix uteri |
| C539 | Malignant neoplasm of cervix uteri, unspecified |
| C540 | Malignant neoplasm of isthmus uteri |
| C541 | Malignant neoplasm of endometrium |
| C542 | Malignant neoplasm of myometrium |
| C543 | Malignant neoplasm of fundus uteri |
| C548 | Malignant neoplasm of overlapping sites of corpus uteri |
| C549 | Malignant neoplasm of corpus uteri, unspecified |
| C55 | Malignant neoplasm of uterus, part unspecified |
| C561 | Malignant neoplasm of right ovary |
| C562 | Malignant neoplasm of left ovary |
| C569 | Malignant neoplasm of unspecified ovary |
| C5700 | Malignant neoplasm of unspecified fallopian tube |
| C5701 | Malignant neoplasm of right fallopian tube |
| C5702 | Malignant neoplasm of left fallopian tube |
| C5710 | Malignant neoplasm of unspecified broad ligament |
| C5711 | Malignant neoplasm of right broad ligament |
| C5712 | Malignant neoplasm of left broad ligament |
| C5720 | Malignant neoplasm of unspecified round ligament |
| C5721 | Malignant neoplasm of right round ligament |
| C5722 | Malignant neoplasm of left round ligament |
| C573 | Malignant neoplasm of parametrium |
| C574 | Malignant neoplasm of uterine adnexa, unspecified |
| C577 | Malignant neoplasm of other specified female genital organs |
| C578 | Malignant neoplasm of overlapping sites of female genital organs |
| C579 | Malignant neoplasm of female genital organ, unspecified |
| C58 | Malignant neoplasm of placenta |
| C600 | Malignant neoplasm of prepuce |
| C601 | Malignant neoplasm of glans penis |
| C602 | Malignant neoplasm of body of penis |
| C608 | Malignant neoplasm of overlapping sites of penis |
| C609 | Malignant neoplasm of penis, unspecified |
| C61 | Malignant neoplasm of prostate |
| C6200 | Malignant neoplasm of unspecified undescended testis |
| C6201 | Malignant neoplasm of undescended right testis |
| C6202 | Malignant neoplasm of undescended left testis |
| C6210 | Malignant neoplasm of unspecified descended testis |
| C6211 | Malignant neoplasm of descended right testis |
| C6212 | Malignant neoplasm of descended left testis |
| C6290 | Malignant neoplasm of unspecified testis, unspecified whether descended or undescended |
| C6291 | Malignant neoplasm of right testis, unspecified whether descended or undescended |
| C6292 | Malignant neoplasm of left testis, unspecified whether descended or undescended |
| C6300 | Malignant neoplasm of unspecified epididymis |
| C6301 | Malignant neoplasm of right epididymis |
| C6302 | Malignant neoplasm of left epididymis |
| C6310 | Malignant neoplasm of unspecified spermatic cord |
| C6311 | Malignant neoplasm of right spermatic cord |
| C6312 | Malignant neoplasm of left spermatic cord |
| C632 | Malignant neoplasm of scrotum |
| C637 | Malignant neoplasm of other specified male genital organs |
| C638 | Malignant neoplasm of overlapping sites of male genital organs |
| C639 | Malignant neoplasm of male genital organ, unspecified |
| C641 | Malignant neoplasm of right kidney, except renal pelvis |
| C642 | Malignant neoplasm of left kidney, except renal pelvis |
| C649 | Malignant neoplasm of unspecified kidney, except renal pelvis |
| C651 | Malignant neoplasm of right renal pelvis |
| C652 | Malignant neoplasm of left renal pelvis |
| C659 | Malignant neoplasm of unspecified renal pelvis |
| C661 | Malignant neoplasm of right ureter |
| C662 | Malignant neoplasm of left ureter |
| C669 | Malignant neoplasm of unspecified ureter |
| C670 | Malignant neoplasm of trigone of bladder |
| C671 | Malignant neoplasm of dome of bladder |
| C672 | Malignant neoplasm of lateral wall of bladder |
| C673 | Malignant neoplasm of anterior wall of bladder |
| C674 | Malignant neoplasm of posterior wall of bladder |
| C675 | Malignant neoplasm of bladder neck |
| C676 | Malignant neoplasm of ureteric orifice |
| C677 | Malignant neoplasm of urachus |
| C678 | Malignant neoplasm of overlapping sites of bladder |
| C679 | Malignant neoplasm of bladder, unspecified |
| C680 | Malignant neoplasm of urethra |
| C681 | Malignant neoplasm of paraurethral glands |
| C688 | Malignant neoplasm of overlapping sites of urinary organs |
| C689 | Malignant neoplasm of urinary organ, unspecified |
| C6900 | Malignant neoplasm of unspecified conjunctiva |
| C6901 | Malignant neoplasm of right conjunctiva |
| C6902 | Malignant neoplasm of left conjunctiva |
| C6910 | Malignant neoplasm of unspecified cornea |
| C6911 | Malignant neoplasm of right cornea |
| C6912 | Malignant neoplasm of left cornea |
| C6920 | Malignant neoplasm of unspecified retina |
| C6921 | Malignant neoplasm of right retina |
| C6922 | Malignant neoplasm of left retina |
| C6930 | Malignant neoplasm of unspecified choroid |
| C6931 | Malignant neoplasm of right choroid |
| C6932 | Malignant neoplasm of left choroid |
| C6940 | Malignant neoplasm of unspecified ciliary body |
| C6941 | Malignant neoplasm of right ciliary body |
| C6942 | Malignant neoplasm of left ciliary body |
| C6950 | Malignant neoplasm of unspecified lacrimal gland and duct |
| C6951 | Malignant neoplasm of right lacrimal gland and duct |
| C6952 | Malignant neoplasm of left lacrimal gland and duct |
| C6960 | Malignant neoplasm of unspecified orbit |
| C6961 | Malignant neoplasm of right orbit |
| C6962 | Malignant neoplasm of left orbit |
| C6980 | Malignant neoplasm of overlapping sites of unspecified eye and adnexa |
| C6981 | Malignant neoplasm of overlapping sites of right eye and adnexa |
| C6982 | Malignant neoplasm of overlapping sites of left eye and adnexa |
| C6990 | Malignant neoplasm of unspecified site of unspecified eye |
| C6991 | Malignant neoplasm of unspecified site of right eye |
| C6992 | Malignant neoplasm of unspecified site of left eye |
| C700 | Malignant neoplasm of cerebral meninges |
| C701 | Malignant neoplasm of spinal meninges |
| C709 | Malignant neoplasm of meninges, unspecified |
| C710 | Malignant neoplasm of cerebrum, except lobes and ventricles |
| C711 | Malignant neoplasm of frontal lobe |
| C712 | Malignant neoplasm of temporal lobe |
| C713 | Malignant neoplasm of parietal lobe |
| C714 | Malignant neoplasm of occipital lobe |
| C715 | Malignant neoplasm of cerebral ventricle |
| C716 | Malignant neoplasm of cerebellum |
| C717 | Malignant neoplasm of brain stem |
| C718 | Malignant neoplasm of overlapping sites of brain |
| C719 | Malignant neoplasm of brain, unspecified |
| C720 | Malignant neoplasm of spinal cord |
| C721 | Malignant neoplasm of cauda equina |
| C7220 | Malignant neoplasm of unspecified olfactory nerve |
| C7221 | Malignant neoplasm of right olfactory nerve |
| C7222 | Malignant neoplasm of left olfactory nerve |
| C7230 | Malignant neoplasm of unspecified optic nerve |
| C7231 | Malignant neoplasm of right optic nerve |
| C7232 | Malignant neoplasm of left optic nerve |
| C7240 | Malignant neoplasm of unspecified acoustic nerve |
| C7241 | Malignant neoplasm of right acoustic nerve |
| C7242 | Malignant neoplasm of left acoustic nerve |
| C7250 | Malignant neoplasm of unspecified cranial nerve |
| C7259 | Malignant neoplasm of other cranial nerves |
| C729 | Malignant neoplasm of central nervous system, unspecified |
| C73 | Malignant neoplasm of thyroid gland |
| C7400 | Malignant neoplasm of cortex of unspecified adrenal gland |
| C7401 | Malignant neoplasm of cortex of right adrenal gland |
| C7402 | Malignant neoplasm of cortex of left adrenal gland |
| C7410 | Malignant neoplasm of medulla of unspecified adrenal gland |
| C7411 | Malignant neoplasm of medulla of right adrenal gland |
| C7412 | Malignant neoplasm of medulla of left adrenal gland |
| C7490 | Malignant neoplasm of unspecified part of unspecified adrenal gland |
| C7491 | Malignant neoplasm of unspecified part of right adrenal gland |
| C7492 | Malignant neoplasm of unspecified part of left adrenal gland |
| C750 | Malignant neoplasm of parathyroid gland |
| C751 | Malignant neoplasm of pituitary gland |
| C752 | Malignant neoplasm of craniopharyngeal duct |
| C753 | Malignant neoplasm of pineal gland |
| C754 | Malignant neoplasm of carotid body |
| C755 | Malignant neoplasm of aortic body and other paraganglia |
| C758 | Malignant neoplasm with pluriglandular involvement, unspecified |
| C759 | Malignant neoplasm of endocrine gland, unspecified |
| C760 | Malignant neoplasm of head, face and neck |
| C761 | Malignant neoplasm of thorax |
| C762 | Malignant neoplasm of abdomen |
| C763 | Malignant neoplasm of pelvis |
| C7640 | Malignant neoplasm of unspecified upper limb |
| C7641 | Malignant neoplasm of right upper limb |
| C7642 | Malignant neoplasm of left upper limb |
| C7650 | Malignant neoplasm of unspecified lower limb |
| C7651 | Malignant neoplasm of right lower limb |
| C7652 | Malignant neoplasm of left lower limb |
| C768 | Malignant neoplasm of other specified ill-defined sites |
| C770 | Secondary and unspecified malignant neoplasm of lymph nodes of head, face and neck |
| C771 | Secondary and unspecified malignant neoplasm of intrathoracic lymph nodes |
| C772 | Secondary and unspecified malignant neoplasm of intra-abdominal lymph nodes |
| C773 | Secondary and unspecified malignant neoplasm of axilla and upper limb lymph nodes |
| C774 | Secondary and unspecified malignant neoplasm of inguinal and lower limb lymph nodes |
| C775 | Secondary and unspecified malignant neoplasm of intrapelvic lymph nodes |
| C778 | Secondary and unspecified malignant neoplasm of lymph nodes of multiple regions |
| C779 | Secondary and unspecified malignant neoplasm of lymph node, unspecified |
| C7800 | Secondary malignant neoplasm of unspecified lung |
| C7801 | Secondary malignant neoplasm of right lung |
| C7802 | Secondary malignant neoplasm of left lung |
| C781 | Secondary malignant neoplasm of mediastinum |
| C782 | Secondary malignant neoplasm of pleura |
| C7830 | Secondary malignant neoplasm of unspecified respiratory organ |
| C7839 | Secondary malignant neoplasm of other respiratory organs |
| C784 | Secondary malignant neoplasm of small intestine |
| C785 | Secondary malignant neoplasm of large intestine and rectum |
| C786 | Secondary malignant neoplasm of retroperitoneum and peritoneum |
| C787 | Secondary malignant neoplasm of liver and intrahepatic bile duct |
| C7880 | Secondary malignant neoplasm of unspecified digestive organ |
| C7889 | Secondary malignant neoplasm of other digestive organs |
| C7900 | Secondary malignant neoplasm of unspecified kidney and renal pelvis |
| C7901 | Secondary malignant neoplasm of right kidney and renal pelvis |
| C7902 | Secondary malignant neoplasm of left kidney and renal pelvis |
| C7910 | Secondary malignant neoplasm of unspecified urinary organs |
| C7911 | Secondary malignant neoplasm of bladder |
| C7919 | Secondary malignant neoplasm of other urinary organs |
| C792 | Secondary malignant neoplasm of skin |
| C7931 | Secondary malignant neoplasm of brain |
| C7932 | Secondary malignant neoplasm of cerebral meninges |
| C7940 | Secondary malignant neoplasm of unspecified part of nervous system |
| C7949 | Secondary malignant neoplasm of other parts of nervous system |
| C7951 | Secondary malignant neoplasm of bone |
| C7952 | Secondary malignant neoplasm of bone marrow |
| C7960 | Secondary malignant neoplasm of unspecified ovary |
| C7961 | Secondary malignant neoplasm of right ovary |
| C7962 | Secondary malignant neoplasm of left ovary |
| C7970 | Secondary malignant neoplasm of unspecified adrenal gland |
| C7971 | Secondary malignant neoplasm of right adrenal gland |
| C7972 | Secondary malignant neoplasm of left adrenal gland |
| C7981 | Secondary malignant neoplasm of breast |
| C7982 | Secondary malignant neoplasm of genital organs |
| C7989 | Secondary malignant neoplasm of other specified sites |
| C799 | Secondary malignant neoplasm of unspecified site |
| C7A00 | Malignant carcinoid tumor of unspecified site |
| C7A010 | Malignant carcinoid tumor of the duodenum |
| C7A011 | Malignant carcinoid tumor of the jejunum |
| C7A012 | Malignant carcinoid tumor of the ileum |
| C7A019 | Malignant carcinoid tumor of the small intestine, unspecified portion |
| C7A020 | Malignant carcinoid tumor of the appendix |
| C7A021 | Malignant carcinoid tumor of the cecum |
| C7A022 | Malignant carcinoid tumor of the ascending colon |
| C7A023 | Malignant carcinoid tumor of the transverse colon |
| C7A024 | Malignant carcinoid tumor of the descending colon |
| C7A025 | Malignant carcinoid tumor of the sigmoid colon |
| C7A026 | Malignant carcinoid tumor of the rectum |
| C7A029 | Malignant carcinoid tumor of the large intestine, unspecified portion |
| C7A090 | Malignant carcinoid tumor of the bronchus and lung |
| C7A091 | Malignant carcinoid tumor of the thymus |
| C7A092 | Malignant carcinoid tumor of the stomach |
| C7A093 | Malignant carcinoid tumor of the kidney |
| C7A094 | Malignant carcinoid tumor of the foregut, unspecified |
| C7A095 | Malignant carcinoid tumor of the midgut, unspecified |
| C7A096 | Malignant carcinoid tumor of the hindgut, unspecified |
| C7A098 | Malignant carcinoid tumors of other sites |
| C7A1 | Malignant poorly differentiated neuroendocrine tumors |
| C7A8 | Other malignant neuroendocrine tumors |
| C7B00 | Secondary carcinoid tumors, unspecified site |
| C7B01 | Secondary carcinoid tumors of distant lymph nodes |
| C7B02 | Secondary carcinoid tumors of liver |
| C7B03 | Secondary carcinoid tumors of bone |
| C7B04 | Secondary carcinoid tumors of peritoneum |
| C7B09 | Secondary carcinoid tumors of other sites |
| C7B1 | Secondary Merkel cell carcinoma |
| C7B8 | Other secondary neuroendocrine tumors |
| C800 | Disseminated malignant neoplasm, unspecified |
| C801 | Malignant (primary) neoplasm, unspecified |
| C802 | Malignant neoplasm associated with transplanted organ |
| C8100 | Nodular lymphocyte predominant Hodgkin lymphoma, unspecified site |
| C8101 | Nodular lymphocyte predominant Hodgkin lymphoma, lymph nodes of head, face, and neck |
| C8102 | Nodular lymphocyte predominant Hodgkin lymphoma, intrathoracic lymph nodes |
| C8103 | Nodular lymphocyte predominant Hodgkin lymphoma, intra-abdominal lymph nodes |
| C8104 | Nodular lymphocyte predominant Hodgkin lymphoma, lymph nodes of axilla and upper limb |
| C8105 | Nodular lymphocyte predominant Hodgkin lymphoma, lymph nodes of inguinal region and lower limb |
| C8106 | Nodular lymphocyte predominant Hodgkin lymphoma, intrapelvic lymph nodes |
| C8107 | Nodular lymphocyte predominant Hodgkin lymphoma, spleen |
| C8108 | Nodular lymphocyte predominant Hodgkin lymphoma, lymph nodes of multiple sites |
| C8109 | Nodular lymphocyte predominant Hodgkin lymphoma, extranodal and solid organ sites |
| C8110 | Nodular sclerosis Hodgkin lymphoma, unspecified site |
| C8111 | Nodular sclerosis Hodgkin lymphoma, lymph nodes of head, face, and neck |
| C8112 | Nodular sclerosis Hodgkin lymphoma, intrathoracic lymph nodes |
| C8113 | Nodular sclerosis Hodgkin lymphoma, intra-abdominal lymph nodes |
| C8114 | Nodular sclerosis Hodgkin lymphoma, lymph nodes of axilla and upper limb |
| C8115 | Nodular sclerosis Hodgkin lymphoma, lymph nodes of inguinal region and lower limb |
| C8116 | Nodular sclerosis Hodgkin lymphoma, intrapelvic lymph nodes |
| C8117 | Nodular sclerosis Hodgkin lymphoma, spleen |
| C8118 | Nodular sclerosis Hodgkin lymphoma, lymph nodes of multiple sites |
| C8119 | Nodular sclerosis Hodgkin lymphoma, extranodal and solid organ sites |
| C8120 | Mixed cellularity Hodgkin lymphoma, unspecified site |
| C8121 | Mixed cellularity Hodgkin lymphoma, lymph nodes of head, face, and neck |
| C8122 | Mixed cellularity Hodgkin lymphoma, intrathoracic lymph nodes |
| C8123 | Mixed cellularity Hodgkin lymphoma, intra-abdominal lymph nodes |
| C8124 | Mixed cellularity Hodgkin lymphoma, lymph nodes of axilla and upper limb |
| C8125 | Mixed cellularity Hodgkin lymphoma, lymph nodes of inguinal region and lower limb |
| C8126 | Mixed cellularity Hodgkin lymphoma, intrapelvic lymph nodes |
| C8127 | Mixed cellularity Hodgkin lymphoma, spleen |
| C8128 | Mixed cellularity Hodgkin lymphoma, lymph nodes of multiple sites |
| C8129 | Mixed cellularity Hodgkin lymphoma, extranodal and solid organ sites |
| C8130 | Lymphocyte depleted Hodgkin lymphoma, unspecified site |
| C8131 | Lymphocyte depleted Hodgkin lymphoma, lymph nodes of head, face, and neck |
| C8132 | Lymphocyte depleted Hodgkin lymphoma, intrathoracic lymph nodes |
| C8133 | Lymphocyte depleted Hodgkin lymphoma, intra-abdominal lymph nodes |
| C8134 | Lymphocyte depleted Hodgkin lymphoma, lymph nodes of axilla and upper limb |
| C8135 | Lymphocyte depleted Hodgkin lymphoma, lymph nodes of inguinal region and lower limb |
| C8136 | Lymphocyte depleted Hodgkin lymphoma, intrapelvic lymph nodes |
| C8137 | Lymphocyte depleted Hodgkin lymphoma, spleen |
| C8138 | Lymphocyte depleted Hodgkin lymphoma, lymph nodes of multiple sites |
| C8139 | Lymphocyte depleted Hodgkin lymphoma, extranodal and solid organ sites |
| C8140 | Lymphocyte-rich Hodgkin lymphoma, unspecified site |
| C8141 | Lymphocyte-rich Hodgkin lymphoma, lymph nodes of head, face, and neck |
| C8142 | Lymphocyte-rich Hodgkin lymphoma, intrathoracic lymph nodes |
| C8143 | Lymphocyte-rich Hodgkin lymphoma, intra-abdominal lymph nodes |
| C8144 | Lymphocyte-rich Hodgkin lymphoma, lymph nodes of axilla and upper limb |
| C8145 | Lymphocyte-rich Hodgkin lymphoma, lymph nodes of inguinal region and lower limb |
| C8146 | Lymphocyte-rich Hodgkin lymphoma, intrapelvic lymph nodes |
| C8147 | Lymphocyte-rich Hodgkin lymphoma, spleen |
| C8148 | Lymphocyte-rich Hodgkin lymphoma, lymph nodes of multiple sites |
| C8149 | Lymphocyte-rich Hodgkin lymphoma, extranodal and solid organ sites |
| C8170 | Other Hodgkin lymphoma, unspecified site |
| C8171 | Other Hodgkin lymphoma, lymph nodes of head, face, and neck |
| C8172 | Other Hodgkin lymphoma, intrathoracic lymph nodes |
| C8173 | Other Hodgkin lymphoma, intra-abdominal lymph nodes |
| C8174 | Other Hodgkin lymphoma, lymph nodes of axilla and upper limb |
| C8175 | Other Hodgkin lymphoma, lymph nodes of inguinal region and lower limb |
| C8176 | Other Hodgkin lymphoma, intrapelvic lymph nodes |
| C8177 | Other Hodgkin lymphoma, spleen |
| C8178 | Other Hodgkin lymphoma, lymph nodes of multiple sites |
| C8179 | Other Hodgkin lymphoma, extranodal and solid organ sites |
| C8190 | Hodgkin lymphoma, unspecified, unspecified site |
| C8191 | Hodgkin lymphoma, unspecified, lymph nodes of head, face, and neck |
| C8192 | Hodgkin lymphoma, unspecified, intrathoracic lymph nodes |
| C8193 | Hodgkin lymphoma, unspecified, intra-abdominal lymph nodes |
| C8194 | Hodgkin lymphoma, unspecified, lymph nodes of axilla and upper limb |
| C8195 | Hodgkin lymphoma, unspecified, lymph nodes of inguinal region and lower limb |
| C8196 | Hodgkin lymphoma, unspecified, intrapelvic lymph nodes |
| C8197 | Hodgkin lymphoma, unspecified, spleen |
| C8198 | Hodgkin lymphoma, unspecified, lymph nodes of multiple sites |
| C8199 | Hodgkin lymphoma, unspecified, extranodal and solid organ sites |
| C8200 | Follicular lymphoma grade I, unspecified site |
| C8201 | Follicular lymphoma grade I, lymph nodes of head, face, and neck |
| C8202 | Follicular lymphoma grade I, intrathoracic lymph nodes |
| C8203 | Follicular lymphoma grade I, intra-abdominal lymph nodes |
| C8204 | Follicular lymphoma grade I, lymph nodes of axilla and upper limb |
| C8205 | Follicular lymphoma grade I, lymph nodes of inguinal region and lower limb |
| C8206 | Follicular lymphoma grade I, intrapelvic lymph nodes |
| C8207 | Follicular lymphoma grade I, spleen |
| C8208 | Follicular lymphoma grade I, lymph nodes of multiple sites |
| C8209 | Follicular lymphoma grade I, extranodal and solid organ sites |
| C8210 | Follicular lymphoma grade II, unspecified site |
| C8211 | Follicular lymphoma grade II, lymph nodes of head, face, and neck |
| C8212 | Follicular lymphoma grade II, intrathoracic lymph nodes |
| C8213 | Follicular lymphoma grade II, intra-abdominal lymph nodes |
| C8214 | Follicular lymphoma grade II, lymph nodes of axilla and upper limb |
| C8215 | Follicular lymphoma grade II, lymph nodes of inguinal region and lower limb |
| C8216 | Follicular lymphoma grade II, intrapelvic lymph nodes |
| C8217 | Follicular lymphoma grade II, spleen |
| C8218 | Follicular lymphoma grade II, lymph nodes of multiple sites |
| C8219 | Follicular lymphoma grade II, extranodal and solid organ sites |
| C8220 | Follicular lymphoma grade III, unspecified, unspecified site |
| C8221 | Follicular lymphoma grade III, unspecified, lymph nodes of head, face, and neck |
| C8222 | Follicular lymphoma grade III, unspecified, intrathoracic lymph nodes |
| C8223 | Follicular lymphoma grade III, unspecified, intra-abdominal lymph nodes |
| C8224 | Follicular lymphoma grade III, unspecified, lymph nodes of axilla and upper limb |
| C8225 | Follicular lymphoma grade III, unspecified, lymph nodes of inguinal region and lower limb |
| C8226 | Follicular lymphoma grade III, unspecified, intrapelvic lymph nodes |
| C8227 | Follicular lymphoma grade III, unspecified, spleen |
| C8228 | Follicular lymphoma grade III, unspecified, lymph nodes of multiple sites |
| C8229 | Follicular lymphoma grade III, unspecified, extranodal and solid organ sites |
| C8230 | Follicular lymphoma grade IIIa, unspecified site |
| C8231 | Follicular lymphoma grade IIIa, lymph nodes of head, face, and neck |
| C8232 | Follicular lymphoma grade IIIa, intrathoracic lymph nodes |
| C8233 | Follicular lymphoma grade IIIa, intra-abdominal lymph nodes |
| C8234 | Follicular lymphoma grade IIIa, lymph nodes of axilla and upper limb |
| C8235 | Follicular lymphoma grade IIIa, lymph nodes of inguinal region and lower limb |
| C8236 | Follicular lymphoma grade IIIa, intrapelvic lymph nodes |
| C8237 | Follicular lymphoma grade IIIa, spleen |
| C8238 | Follicular lymphoma grade IIIa, lymph nodes of multiple sites |
| C8239 | Follicular lymphoma grade IIIa, extranodal and solid organ sites |
| C8240 | Follicular lymphoma grade IIIb, unspecified site |
| C8241 | Follicular lymphoma grade IIIb, lymph nodes of head, face, and neck |
| C8242 | Follicular lymphoma grade IIIb, intrathoracic lymph nodes |
| C8243 | Follicular lymphoma grade IIIb, intra-abdominal lymph nodes |
| C8244 | Follicular lymphoma grade IIIb, lymph nodes of axilla and upper limb |
| C8245 | Follicular lymphoma grade IIIb, lymph nodes of inguinal region and lower limb |
| C8246 | Follicular lymphoma grade IIIb, intrapelvic lymph nodes |
| C8247 | Follicular lymphoma grade IIIb, spleen |
| C8248 | Follicular lymphoma grade IIIb, lymph nodes of multiple sites |
| C8249 | Follicular lymphoma grade IIIb, extranodal and solid organ sites |
| C8250 | Diffuse follicle center lymphoma, unspecified site |
| C8251 | Diffuse follicle center lymphoma, lymph nodes of head, face, and neck |
| C8252 | Diffuse follicle center lymphoma, intrathoracic lymph nodes |
| C8253 | Diffuse follicle center lymphoma, intra-abdominal lymph nodes |
| C8254 | Diffuse follicle center lymphoma, lymph nodes of axilla and upper limb |
| C8255 | Diffuse follicle center lymphoma, lymph nodes of inguinal region and lower limb |
| C8256 | Diffuse follicle center lymphoma, intrapelvic lymph nodes |
| C8257 | Diffuse follicle center lymphoma, spleen |
| C8258 | Diffuse follicle center lymphoma, lymph nodes of multiple sites |
| C8259 | Diffuse follicle center lymphoma, extranodal and solid organ sites |
| C8260 | Cutaneous follicle center lymphoma, unspecified site |
| C8261 | Cutaneous follicle center lymphoma, lymph nodes of head, face, and neck |
| C8262 | Cutaneous follicle center lymphoma, intrathoracic lymph nodes |
| C8263 | Cutaneous follicle center lymphoma, intra-abdominal lymph nodes |
| C8264 | Cutaneous follicle center lymphoma, lymph nodes of axilla and upper limb |
| C8265 | Cutaneous follicle center lymphoma, lymph nodes of inguinal region and lower limb |
| C8266 | Cutaneous follicle center lymphoma, intrapelvic lymph nodes |
| C8267 | Cutaneous follicle center lymphoma, spleen |
| C8268 | Cutaneous follicle center lymphoma, lymph nodes of multiple sites |
| C8269 | Cutaneous follicle center lymphoma, extranodal and solid organ sites |
| C8280 | Other types of follicular lymphoma, unspecified site |
| C8281 | Other types of follicular lymphoma, lymph nodes of head, face, and neck |
| C8282 | Other types of follicular lymphoma, intrathoracic lymph nodes |
| C8283 | Other types of follicular lymphoma, intra-abdominal lymph nodes |
| C8284 | Other types of follicular lymphoma, lymph nodes of axilla and upper limb |
| C8285 | Other types of follicular lymphoma, lymph nodes of inguinal region and lower limb |
| C8286 | Other types of follicular lymphoma, intrapelvic lymph nodes |
| C8287 | Other types of follicular lymphoma, spleen |
| C8288 | Other types of follicular lymphoma, lymph nodes of multiple sites |
| C8289 | Other types of follicular lymphoma, extranodal and solid organ sites |
| C8290 | Follicular lymphoma, unspecified, unspecified site |
| C8291 | Follicular lymphoma, unspecified, lymph nodes of head, face, and neck |
| C8292 | Follicular lymphoma, unspecified, intrathoracic lymph nodes |
| C8293 | Follicular lymphoma, unspecified, intra-abdominal lymph nodes |
| C8294 | Follicular lymphoma, unspecified, lymph nodes of axilla and upper limb |
| C8295 | Follicular lymphoma, unspecified, lymph nodes of inguinal region and lower limb |
| C8296 | Follicular lymphoma, unspecified, intrapelvic lymph nodes |
| C8297 | Follicular lymphoma, unspecified, spleen |
| C8298 | Follicular lymphoma, unspecified, lymph nodes of multiple sites |
| C8299 | Follicular lymphoma, unspecified, extranodal and solid organ sites |
| C8300 | Small cell B-cell lymphoma, unspecified site |
| C8301 | Small cell B-cell lymphoma, lymph nodes of head, face, and neck |
| C8302 | Small cell B-cell lymphoma, intrathoracic lymph nodes |
| C8303 | Small cell B-cell lymphoma, intra-abdominal lymph nodes |
| C8304 | Small cell B-cell lymphoma, lymph nodes of axilla and upper limb |
| C8305 | Small cell B-cell lymphoma, lymph nodes of inguinal region and lower limb |
| C8306 | Small cell B-cell lymphoma, intrapelvic lymph nodes |
| C8307 | Small cell B-cell lymphoma, spleen |
| C8308 | Small cell B-cell lymphoma, lymph nodes of multiple sites |
| C8309 | Small cell B-cell lymphoma, extranodal and solid organ sites |
| C8310 | Mantle cell lymphoma, unspecified site |
| C8311 | Mantle cell lymphoma, lymph nodes of head, face, and neck |
| C8312 | Mantle cell lymphoma, intrathoracic lymph nodes |
| C8313 | Mantle cell lymphoma, intra-abdominal lymph nodes |
| C8314 | Mantle cell lymphoma, lymph nodes of axilla and upper limb |
| C8315 | Mantle cell lymphoma, lymph nodes of inguinal region and lower limb |
| C8316 | Mantle cell lymphoma, intrapelvic lymph nodes |
| C8317 | Mantle cell lymphoma, spleen |
| C8318 | Mantle cell lymphoma, lymph nodes of multiple sites |
| C8319 | Mantle cell lymphoma, extranodal and solid organ sites |
| C8330 | Diffuse large B-cell lymphoma, unspecified site |
| C8331 | Diffuse large B-cell lymphoma, lymph nodes of head, face, and neck |
| C8332 | Diffuse large B-cell lymphoma, intrathoracic lymph nodes |
| C8333 | Diffuse large B-cell lymphoma, intra-abdominal lymph nodes |
| C8334 | Diffuse large B-cell lymphoma, lymph nodes of axilla and upper limb |
| C8335 | Diffuse large B-cell lymphoma, lymph nodes of inguinal region and lower limb |
| C8336 | Diffuse large B-cell lymphoma, intrapelvic lymph nodes |
| C8337 | Diffuse large B-cell lymphoma, spleen |
| C8338 | Diffuse large B-cell lymphoma, lymph nodes of multiple sites |
| C8339 | Diffuse large B-cell lymphoma, extranodal and solid organ sites |
| C8350 | Lymphoblastic (diffuse) lymphoma, unspecified site |
| C8351 | Lymphoblastic (diffuse) lymphoma, lymph nodes of head, face, and neck |
| C8352 | Lymphoblastic (diffuse) lymphoma, intrathoracic lymph nodes |
| C8353 | Lymphoblastic (diffuse) lymphoma, intra-abdominal lymph nodes |
| C8354 | Lymphoblastic (diffuse) lymphoma, lymph nodes of axilla and upper limb |
| C8355 | Lymphoblastic (diffuse) lymphoma, lymph nodes of inguinal region and lower limb |
| C8356 | Lymphoblastic (diffuse) lymphoma, intrapelvic lymph nodes |
| C8357 | Lymphoblastic (diffuse) lymphoma, spleen |
| C8358 | Lymphoblastic (diffuse) lymphoma, lymph nodes of multiple sites |
| C8359 | Lymphoblastic (diffuse) lymphoma, extranodal and solid organ sites |
| C8370 | Burkitt lymphoma, unspecified site |
| C8371 | Burkitt lymphoma, lymph nodes of head, face, and neck |
| C8372 | Burkitt lymphoma, intrathoracic lymph nodes |
| C8373 | Burkitt lymphoma, intra-abdominal lymph nodes |
| C8374 | Burkitt lymphoma, lymph nodes of axilla and upper limb |
| C8375 | Burkitt lymphoma, lymph nodes of inguinal region and lower limb |
| C8376 | Burkitt lymphoma, intrapelvic lymph nodes |
| C8377 | Burkitt lymphoma, spleen |
| C8378 | Burkitt lymphoma, lymph nodes of multiple sites |
| C8379 | Burkitt lymphoma, extranodal and solid organ sites |
| C8380 | Other non-follicular lymphoma, unspecified site |
| C8381 | Other non-follicular lymphoma, lymph nodes of head, face, and neck |
| C8382 | Other non-follicular lymphoma, intrathoracic lymph nodes |
| C8383 | Other non-follicular lymphoma, intra-abdominal lymph nodes |
| C8384 | Other non-follicular lymphoma, lymph nodes of axilla and upper limb |
| C8385 | Other non-follicular lymphoma, lymph nodes of inguinal region and lower limb |
| C8386 | Other non-follicular lymphoma, intrapelvic lymph nodes |
| C8387 | Other non-follicular lymphoma, spleen |
| C8388 | Other non-follicular lymphoma, lymph nodes of multiple sites |
| C8389 | Other non-follicular lymphoma, extranodal and solid organ sites |
| C8390 | Non-follicular (diffuse) lymphoma, unspecified, unspecified site |
| C8391 | Non-follicular (diffuse) lymphoma, unspecified, lymph nodes of head, face, and neck |
| C8392 | Non-follicular (diffuse) lymphoma, unspecified, intrathoracic lymph nodes |
| C8393 | Non-follicular (diffuse) lymphoma, unspecified, intra-abdominal lymph nodes |
| C8394 | Non-follicular (diffuse) lymphoma, unspecified, lymph nodes of axilla and upper limb |
| C8395 | Non-follicular (diffuse) lymphoma, unspecified, lymph nodes of inguinal region and lower limb |
| C8396 | Non-follicular (diffuse) lymphoma, unspecified, intrapelvic lymph nodes |
| C8397 | Non-follicular (diffuse) lymphoma, unspecified, spleen |
| C8398 | Non-follicular (diffuse) lymphoma, unspecified, lymph nodes of multiple sites |
| C8399 | Non-follicular (diffuse) lymphoma, unspecified, extranodal and solid organ sites |
| C8400 | Mycosis fungoides, unspecified site |
| C8401 | Mycosis fungoides, lymph nodes of head, face, and neck |
| C8402 | Mycosis fungoides, intrathoracic lymph nodes |
| C8403 | Mycosis fungoides, intra-abdominal lymph nodes |
| C8404 | Mycosis fungoides, lymph nodes of axilla and upper limb |
| C8405 | Mycosis fungoides, lymph nodes of inguinal region and lower limb |
| C8406 | Mycosis fungoides, intrapelvic lymph nodes |
| C8407 | Mycosis fungoides, spleen |
| C8408 | Mycosis fungoides, lymph nodes of multiple sites |
| C8409 | Mycosis fungoides, extranodal and solid organ sites |
| C8410 | Sezary disease, unspecified site |
| C8411 | Sezary disease, lymph nodes of head, face, and neck |
| C8412 | Sezary disease, intrathoracic lymph nodes |
| C8413 | Sezary disease, intra-abdominal lymph nodes |
| C8414 | Sezary disease, lymph nodes of axilla and upper limb |
| C8415 | Sezary disease, lymph nodes of inguinal region and lower limb |
| C8416 | Sezary disease, intrapelvic lymph nodes |
| C8417 | Sezary disease, spleen |
| C8418 | Sezary disease, lymph nodes of multiple sites |
| C8419 | Sezary disease, extranodal and solid organ sites |
| C8440 | Peripheral T-cell lymphoma, not classified, unspecified site |
| C8441 | Peripheral T-cell lymphoma, not classified, lymph nodes of head, face, and neck |
| C8442 | Peripheral T-cell lymphoma, not classified, intrathoracic lymph nodes |
| C8443 | Peripheral T-cell lymphoma, not classified, intra-abdominal lymph nodes |
| C8444 | Peripheral T-cell lymphoma, not classified, lymph nodes of axilla and upper limb |
| C8445 | Peripheral T-cell lymphoma, not classified, lymph nodes of inguinal region and lower limb |
| C8446 | Peripheral T-cell lymphoma, not classified, intrapelvic lymph nodes |
| C8447 | Peripheral T-cell lymphoma, not classified, spleen |
| C8448 | Peripheral T-cell lymphoma, not classified, lymph nodes of multiple sites |
| C8449 | Peripheral T-cell lymphoma, not classified, extranodal and solid organ sites |
| C8460 | Anaplastic large cell lymphoma, ALK-positive, unspecified site |
| C8461 | Anaplastic large cell lymphoma, ALK-positive, lymph nodes of head, face, and neck |
| C8462 | Anaplastic large cell lymphoma, ALK-positive, intrathoracic lymph nodes |
| C8463 | Anaplastic large cell lymphoma, ALK-positive, intra-abdominal lymph nodes |
| C8464 | Anaplastic large cell lymphoma, ALK-positive, lymph nodes of axilla and upper limb |
| C8465 | Anaplastic large cell lymphoma, ALK-positive, lymph nodes of inguinal region and lower limb |
| C8466 | Anaplastic large cell lymphoma, ALK-positive, intrapelvic lymph nodes |
| C8467 | Anaplastic large cell lymphoma, ALK-positive, spleen |
| C8468 | Anaplastic large cell lymphoma, ALK-positive, lymph nodes of multiple sites |
| C8469 | Anaplastic large cell lymphoma, ALK-positive, extranodal and solid organ sites |
| C8470 | Anaplastic large cell lymphoma, ALK-negative, unspecified site |
| C8471 | Anaplastic large cell lymphoma, ALK-negative, lymph nodes of head, face, and neck |
| C8472 | Anaplastic large cell lymphoma, ALK-negative, intrathoracic lymph nodes |
| C8473 | Anaplastic large cell lymphoma, ALK-negative, intra-abdominal lymph nodes |
| C8474 | Anaplastic large cell lymphoma, ALK-negative, lymph nodes of axilla and upper limb |
| C8475 | Anaplastic large cell lymphoma, ALK-negative, lymph nodes of inguinal region and lower limb |
| C8476 | Anaplastic large cell lymphoma, ALK-negative, intrapelvic lymph nodes |
| C8477 | Anaplastic large cell lymphoma, ALK-negative, spleen |
| C8478 | Anaplastic large cell lymphoma, ALK-negative, lymph nodes of multiple sites |
| C8479 | Anaplastic large cell lymphoma, ALK-negative, extranodal and solid organ sites |
| C8490 | Mature T/NK-cell lymphomas, unspecified, unspecified site |
| C8491 | Mature T/NK-cell lymphomas, unspecified, lymph nodes of head, face, and neck |
| C8492 | Mature T/NK-cell lymphomas, unspecified, intrathoracic lymph nodes |
| C8493 | Mature T/NK-cell lymphomas, unspecified, intra-abdominal lymph nodes |
| C8494 | Mature T/NK-cell lymphomas, unspecified, lymph nodes of axilla and upper limb |
| C8495 | Mature T/NK-cell lymphomas, unspecified, lymph nodes of inguinal region and lower limb |
| C8496 | Mature T/NK-cell lymphomas, unspecified, intrapelvic lymph nodes |
| C8497 | Mature T/NK-cell lymphomas, unspecified, spleen |
| C8498 | Mature T/NK-cell lymphomas, unspecified, lymph nodes of multiple sites |
| C8499 | Mature T/NK-cell lymphomas, unspecified, extranodal and solid organ sites |
| C84A0 | Cutaneous T-cell lymphoma, unspecified, unspecified site |
| C84A1 | Cutaneous T-cell lymphoma, unspecified lymph nodes of head, face, and neck |
| C84A2 | Cutaneous T-cell lymphoma, unspecified, intrathoracic lymph nodes |
| C84A3 | Cutaneous T-cell lymphoma, unspecified, intra-abdominal lymph nodes |
| C84A4 | Cutaneous T-cell lymphoma, unspecified, lymph nodes of axilla and upper limb |
| C84A5 | Cutaneous T-cell lymphoma, unspecified, lymph nodes of inguinal region and lower limb |
| C84A6 | Cutaneous T-cell lymphoma, unspecified, intrapelvic lymph nodes |
| C84A7 | Cutaneous T-cell lymphoma, unspecified, spleen |
| C84A8 | Cutaneous T-cell lymphoma, unspecified, lymph nodes of multiple sites |
| C84A9 | Cutaneous T-cell lymphoma, unspecified, extranodal and solid organ sites |
| C84Z0 | Other mature T/NK-cell lymphomas, unspecified site |
| C84Z1 | Other mature T/NK-cell lymphomas, lymph nodes of head, face, and neck |
| C84Z2 | Other mature T/NK-cell lymphomas, intrathoracic lymph nodes |
| C84Z3 | Other mature T/NK-cell lymphomas, intra-abdominal lymph nodes |
| C84Z4 | Other mature T/NK-cell lymphomas, lymph nodes of axilla and upper limb |
| C84Z5 | Other mature T/NK-cell lymphomas, lymph nodes of inguinal region and lower limb |
| C84Z6 | Other mature T/NK-cell lymphomas, intrapelvic lymph nodes |
| C84Z7 | Other mature T/NK-cell lymphomas, spleen |
| C84Z8 | Other mature T/NK-cell lymphomas, lymph nodes of multiple sites |
| C84Z9 | Other mature T/NK-cell lymphomas, extranodal and solid organ sites |
| C8510 | Unspecified B-cell lymphoma, unspecified site |
| C8511 | Unspecified B-cell lymphoma, lymph nodes of head, face, and neck |
| C8512 | Unspecified B-cell lymphoma, intrathoracic lymph nodes |
| C8513 | Unspecified B-cell lymphoma, intra-abdominal lymph nodes |
| C8514 | Unspecified B-cell lymphoma, lymph nodes of axilla and upper limb |
| C8515 | Unspecified B-cell lymphoma, lymph nodes of inguinal region and lower limb |
| C8516 | Unspecified B-cell lymphoma, intrapelvic lymph nodes |
| C8517 | Unspecified B-cell lymphoma, spleen |
| C8518 | Unspecified B-cell lymphoma, lymph nodes of multiple sites |
| C8519 | Unspecified B-cell lymphoma, extranodal and solid organ sites |
| C8520 | Mediastinal (thymic) large B-cell lymphoma, unspecified site |
| C8521 | Mediastinal (thymic) large B-cell lymphoma, lymph nodes of head, face, and neck |
| C8522 | Mediastinal (thymic) large B-cell lymphoma, intrathoracic lymph nodes |
| C8523 | Mediastinal (thymic) large B-cell lymphoma, intra-abdominal lymph nodes |
| C8524 | Mediastinal (thymic) large B-cell lymphoma, lymph nodes of axilla and upper limb |
| C8525 | Mediastinal (thymic) large B-cell lymphoma, lymph nodes of inguinal region and lower limb |
| C8526 | Mediastinal (thymic) large B-cell lymphoma, intrapelvic lymph nodes |
| C8527 | Mediastinal (thymic) large B-cell lymphoma, spleen |
| C8528 | Mediastinal (thymic) large B-cell lymphoma, lymph nodes of multiple sites |
| C8529 | Mediastinal (thymic) large B-cell lymphoma, extranodal and solid organ sites |
| C8580 | Other specified types of non-Hodgkin lymphoma, unspecified site |
| C8581 | Other specified types of non-Hodgkin lymphoma, lymph nodes of head, face, and neck |
| C8582 | Other specified types of non-Hodgkin lymphoma, intrathoracic lymph nodes |
| C8583 | Other specified types of non-Hodgkin lymphoma, intra-abdominal lymph nodes |
| C8584 | Other specified types of non-Hodgkin lymphoma, lymph nodes of axilla and upper limb |
| C8585 | Other specified types of non-Hodgkin lymphoma, lymph nodes of inguinal region and lower limb |
| C8586 | Other specified types of non-Hodgkin lymphoma, intrapelvic lymph nodes |
| C8587 | Other specified types of non-Hodgkin lymphoma, spleen |
| C8588 | Other specified types of non-Hodgkin lymphoma, lymph nodes of multiple sites |
| C8589 | Other specified types of non-Hodgkin lymphoma, extranodal and solid organ sites |
| C8590 | Non-Hodgkin lymphoma, unspecified, unspecified site |
| C8591 | Non-Hodgkin lymphoma, unspecified, lymph nodes of head, face, and neck |
| C8592 | Non-Hodgkin lymphoma, unspecified, intrathoracic lymph nodes |
| C8593 | Non-Hodgkin lymphoma, unspecified, intra-abdominal lymph nodes |
| C8594 | Non-Hodgkin lymphoma, unspecified, lymph nodes of axilla and upper limb |
| C8595 | Non-Hodgkin lymphoma, unspecified, lymph nodes of inguinal region and lower limb |
| C8596 | Non-Hodgkin lymphoma, unspecified, intrapelvic lymph nodes |
| C8597 | Non-Hodgkin lymphoma, unspecified, spleen |
| C8598 | Non-Hodgkin lymphoma, unspecified, lymph nodes of multiple sites |
| C8599 | Non-Hodgkin lymphoma, unspecified, extranodal and solid organ sites |
| C860 | Extranodal NK/T-cell lymphoma, nasal type |
| C861 | Hepatosplenic T-cell lymphoma |
| C862 | Enteropathy-type (intestinal) T-cell lymphoma |
| C863 | Subcutaneous panniculitis-like T-cell lymphoma |
| C864 | Blastic NK-cell lymphoma |
| C865 | Angioimmunoblastic T-cell lymphoma |
| C866 | Primary cutaneous CD30-positive T-cell proliferations |
| C880 | Waldenstrom macroglobulinemia |
| C882 | Heavy chain disease |
| C883 | Immunoproliferative small intestinal disease |
| C884 | Extranodal marginal zone B-cell lymphoma of mucosa-associated lymphoid tissue [MALT-lymphoma] |
| C888 | Other malignant immunoproliferative diseases |
| C889 | Malignant immunoproliferative disease, unspecified |
| C9000 | Multiple myeloma not having achieved remission |
| C9001 | Multiple myeloma in remission |
| C9002 | Multiple myeloma in relapse |
| C9010 | Plasma cell leukemia not having achieved remission |
| C9011 | Plasma cell leukemia in remission |
| C9012 | Plasma cell leukemia in relapse |
| C9020 | Extramedullary plasmacytoma not having achieved remission |
| C9021 | Extramedullary plasmacytoma in remission |
| C9022 | Extramedullary plasmacytoma in relapse |
| C9030 | Solitary plasmacytoma not having achieved remission |
| C9031 | Solitary plasmacytoma in remission |
| C9032 | Solitary plasmacytoma in relapse |
| C9100 | Acute lymphoblastic leukemia not having achieved remission |
| C9101 | Acute lymphoblastic leukemia, in remission |
| C9102 | Acute lymphoblastic leukemia, in relapse |
| C9110 | Chronic lymphocytic leukemia of B-cell type not having achieved remission |
| C9111 | Chronic lymphocytic leukemia of B-cell type in remission |
| C9112 | Chronic lymphocytic leukemia of B-cell type in relapse |
| C9130 | Prolymphocytic leukemia of B-cell type not having achieved remission |
| C9131 | Prolymphocytic leukemia of B-cell type, in remission |
| C9132 | Prolymphocytic leukemia of B-cell type, in relapse |
| C9140 | Hairy cell leukemia not having achieved remission |
| C9141 | Hairy cell leukemia, in remission |
| C9142 | Hairy cell leukemia, in relapse |
| C9150 | Adult T-cell lymphoma/leukemia (HTLV-1-associated) not having achieved remission |
| C9151 | Adult T-cell lymphoma/leukemia (HTLV-1-associated), in remission |
| C9152 | Adult T-cell lymphoma/leukemia (HTLV-1-associated), in relapse |
| C9160 | Prolymphocytic leukemia of T-cell type not having achieved remission |
| C9161 | Prolymphocytic leukemia of T-cell type, in remission |
| C9162 | Prolymphocytic leukemia of T-cell type, in relapse |
| C9190 | Lymphoid leukemia, unspecified not having achieved remission |
| C9191 | Lymphoid leukemia, unspecified, in remission |
| C9192 | Lymphoid leukemia, unspecified, in relapse |
| C91A0 | Mature B-cell leukemia Burkitt-type not having achieved remission |
| C91A1 | Mature B-cell leukemia Burkitt-type, in remission |
| C91A2 | Mature B-cell leukemia Burkitt-type, in relapse |
| C91Z0 | Other lymphoid leukemia not having achieved remission |
| C91Z1 | Other lymphoid leukemia, in remission |
| C91Z2 | Other lymphoid leukemia, in relapse |
| C9200 | Acute myeloblastic leukemia, not having achieved remission |
| C9201 | Acute myeloblastic leukemia, in remission |
| C9202 | Acute myeloblastic leukemia, in relapse |
| C9210 | Chronic myeloid leukemia, BCR/ABL-positive, not having achieved remission |
| C9211 | Chronic myeloid leukemia, BCR/ABL-positive, in remission |
| C9212 | Chronic myeloid leukemia, BCR/ABL-positive, in relapse |
| C9220 | Atypical chronic myeloid leukemia, BCR/ABL-negative, not having achieved remission |
| C9221 | Atypical chronic myeloid leukemia, BCR/ABL-negative, in remission |
| C9222 | Atypical chronic myeloid leukemia, BCR/ABL-negative, in relapse |
| C9230 | Myeloid sarcoma, not having achieved remission |
| C9231 | Myeloid sarcoma, in remission |
| C9232 | Myeloid sarcoma, in relapse |
| C9240 | Acute promyelocytic leukemia, not having achieved remission |
| C9241 | Acute promyelocytic leukemia, in remission |
| C9242 | Acute promyelocytic leukemia, in relapse |
| C9250 | Acute myelomonocytic leukemia, not having achieved remission |
| C9251 | Acute myelomonocytic leukemia, in remission |
| C9252 | Acute myelomonocytic leukemia, in relapse |
| C9260 | Acute myeloid leukemia with 11q23-abnormality not having achieved remission |
| C9261 | Acute myeloid leukemia with 11q23-abnormality in remission |
| C9262 | Acute myeloid leukemia with 11q23-abnormality in relapse |
| C9290 | Myeloid leukemia, unspecified, not having achieved remission |
| C9291 | Myeloid leukemia, unspecified in remission |
| C9292 | Myeloid leukemia, unspecified in relapse |
| C92A0 | Acute myeloid leukemia with multilineage dysplasia, not having achieved remission |
| C92A1 | Acute myeloid leukemia with multilineage dysplasia, in remission |
| C92A2 | Acute myeloid leukemia with multilineage dysplasia, in relapse |
| C92Z0 | Other myeloid leukemia not having achieved remission |
| C92Z1 | Other myeloid leukemia, in remission |
| C92Z2 | Other myeloid leukemia, in relapse |
| C9300 | Acute monoblastic/monocytic leukemia, not having achieved remission |
| C9301 | Acute monoblastic/monocytic leukemia, in remission |
| C9302 | Acute monoblastic/monocytic leukemia, in relapse |
| C9310 | Chronic myelomonocytic leukemia not having achieved remission |
| C9311 | Chronic myelomonocytic leukemia, in remission |
| C9312 | Chronic myelomonocytic leukemia, in relapse |
| C9330 | Juvenile myelomonocytic leukemia, not having achieved remission |
| C9331 | Juvenile myelomonocytic leukemia, in remission |
| C9332 | Juvenile myelomonocytic leukemia, in relapse |
| C9390 | Monocytic leukemia, unspecified, not having achieved remission |
| C9391 | Monocytic leukemia, unspecified in remission |
| C9392 | Monocytic leukemia, unspecified in relapse |
| C93Z0 | Other monocytic leukemia, not having achieved remission |
| C93Z1 | Other monocytic leukemia, in remission |
| C93Z2 | Other monocytic leukemia, in relapse |
| C9400 | Acute erythroid leukemia, not having achieved remission |
| C9401 | Acute erythroid leukemia, in remission |
| C9402 | Acute erythroid leukemia, in relapse |
| C9420 | Acute megakaryoblastic leukemia not having achieved remission |
| C9421 | Acute megakaryoblastic leukemia, in remission |
| C9422 | Acute megakaryoblastic leukemia, in relapse |
| C9430 | Mast cell leukemia not having achieved remission |
| C9431 | Mast cell leukemia, in remission |
| C9432 | Mast cell leukemia, in relapse |
| C9440 | Acute panmyelosis with myelofibrosis not having achieved remission |
| C9441 | Acute panmyelosis with myelofibrosis, in remission |
| C9442 | Acute panmyelosis with myelofibrosis, in relapse |
| C946 | Myelodysplastic disease, not classified |
| C9480 | Other specified leukemias not having achieved remission |
| C9481 | Other specified leukemias, in remission |
| C9482 | Other specified leukemias, in relapse |
| C9500 | Acute leukemia of unspecified cell type not having achieved remission |
| C9501 | Acute leukemia of unspecified cell type, in remission |
| C9502 | Acute leukemia of unspecified cell type, in relapse |
| C9510 | Chronic leukemia of unspecified cell type not having achieved remission |
| C9511 | Chronic leukemia of unspecified cell type, in remission |
| C9512 | Chronic leukemia of unspecified cell type, in relapse |
| C9590 | Leukemia, unspecified not having achieved remission |
| C9591 | Leukemia, unspecified, in remission |
| C9592 | Leukemia, unspecified, in relapse |
| C960 | Multifocal and multisystemic (disseminated) Langerhans-cell histiocytosis |
| C9620 | Malignant mast cell neoplasm, unspecified |
| C9621 | Aggressive systemic mastocytosis |
| C9622 | Mast cell sarcoma |
| C9629 | Other malignant mast cell neoplasm |
| C964 | Sarcoma of dendritic cells (accessory cells) |
| C965 | Multifocal and unisystemic Langerhans-cell histiocytosis |
| C966 | Unifocal Langerhans-cell histiocytosis |
| C969 | Malignant neoplasm of lymphoid, hematopoietic and related tissue, unspecified |
| C96A | Histiocytic sarcoma |
| C96Z | Other specified malignant neoplasms of lymphoid, hematopoietic and related tissue |
| D0000 | Carcinoma in situ of oral cavity, unspecified site |
| D0001 | Carcinoma in situ of labial mucosa and vermilion border |
| D0002 | Carcinoma in situ of buccal mucosa |
| D0003 | Carcinoma in situ of gingiva and edentulous alveolar ridge |
| D0004 | Carcinoma in situ of soft palate |
| D0005 | Carcinoma in situ of hard palate |
| D0006 | Carcinoma in situ of floor of mouth |
| D0007 | Carcinoma in situ of tongue |
| D0008 | Carcinoma in situ of pharynx |
| D001 | Carcinoma in situ of esophagus |
| D002 | Carcinoma in situ of stomach |
| D010 | Carcinoma in situ of colon |
| D011 | Carcinoma in situ of rectosigmoid junction |
| D012 | Carcinoma in situ of rectum |
| D013 | Carcinoma in situ of anus and anal canal |
| D0140 | Carcinoma in situ of unspecified part of intestine |
| D0149 | Carcinoma in situ of other parts of intestine |
| D015 | Carcinoma in situ of liver, gallbladder and bile ducts |
| D017 | Carcinoma in situ of other specified digestive organs |
| D019 | Carcinoma in situ of digestive organ, unspecified |
| D020 | Carcinoma in situ of larynx |
| D021 | Carcinoma in situ of trachea |
| D0220 | Carcinoma in situ of unspecified bronchus and lung |
| D0221 | Carcinoma in situ of right bronchus and lung |
| D0222 | Carcinoma in situ of left bronchus and lung |
| D023 | Carcinoma in situ of other parts of respiratory system |
| D024 | Carcinoma in situ of respiratory system, unspecified |
| D030 | Melanoma in situ of lip |
| D0310 | Melanoma in situ of unspecified eyelid, including canthus |
| D03111 | Melanoma in situ of right upper eyelid, including canthus |
| D03112 | Melanoma in situ of right lower eyelid, including canthus |
| D03121 | Melanoma in situ of left upper eyelid, including canthus |
| D03122 | Melanoma in situ of left lower eyelid, including canthus |
| D0320 | Melanoma in situ of unspecified ear and external auricular canal |
| D0321 | Melanoma in situ of right ear and external auricular canal |
| D0322 | Melanoma in situ of left ear and external auricular canal |
| D0330 | Melanoma in situ of unspecified part of face |
| D0339 | Melanoma in situ of other parts of face |
| D034 | Melanoma in situ of scalp and neck |
| D0351 | Melanoma in situ of anal skin |
| D0352 | Melanoma in situ of breast (skin) (soft tissue) |
| D0359 | Melanoma in situ of other part of trunk |
| D0360 | Melanoma in situ of unspecified upper limb, including shoulder |
| D0361 | Melanoma in situ of right upper limb, including shoulder |
| D0362 | Melanoma in situ of left upper limb, including shoulder |
| D0370 | Melanoma in situ of unspecified lower limb, including hip |
| D0371 | Melanoma in situ of right lower limb, including hip |
| D0372 | Melanoma in situ of left lower limb, including hip |
| D038 | Melanoma in situ of other sites |
| D039 | Melanoma in situ, unspecified |
| D040 | Carcinoma in situ of skin of lip |
| D0410 | Carcinoma in situ of skin of unspecified eyelid, including canthus |
| D04111 | Carcinoma in situ of skin of right upper eyelid, including canthus |
| D04112 | Carcinoma in situ of skin of right lower eyelid, including canthus |
| D04121 | Carcinoma in situ of skin of left upper eyelid, including canthus |
| D04122 | Carcinoma in situ of skin of left lower eyelid, including canthus |
| D0420 | Carcinoma in situ of skin of unspecified ear and external auricular canal |
| D0421 | Carcinoma in situ of skin of right ear and external auricular canal |
| D0422 | Carcinoma in situ of skin of left ear and external auricular canal |
| D0430 | Carcinoma in situ of skin of unspecified part of face |
| D0439 | Carcinoma in situ of skin of other parts of face |
| D044 | Carcinoma in situ of skin of scalp and neck |
| D045 | Carcinoma in situ of skin of trunk |
| D0460 | Carcinoma in situ of skin of unspecified upper limb, including shoulder |
| D0461 | Carcinoma in situ of skin of right upper limb, including shoulder |
| D0462 | Carcinoma in situ of skin of left upper limb, including shoulder |
| D0470 | Carcinoma in situ of skin of unspecified lower limb, including hip |
| D0471 | Carcinoma in situ of skin of right lower limb, including hip |
| D0472 | Carcinoma in situ of skin of left lower limb, including hip |
| D048 | Carcinoma in situ of skin of other sites |
| D049 | Carcinoma in situ of skin, unspecified |
| D0500 | Lobular carcinoma in situ of unspecified breast |
| D0501 | Lobular carcinoma in situ of right breast |
| D0502 | Lobular carcinoma in situ of left breast |
| D0510 | Intraductal carcinoma in situ of unspecified breast |
| D0511 | Intraductal carcinoma in situ of right breast |
| D0512 | Intraductal carcinoma in situ of left breast |
| D0580 | Other specified type of carcinoma in situ of unspecified breast |
| D0581 | Other specified type of carcinoma in situ of right breast |
| D0582 | Other specified type of carcinoma in situ of left breast |
| D0590 | Unspecified type of carcinoma in situ of unspecified breast |
| D0591 | Unspecified type of carcinoma in situ of right breast |
| D0592 | Unspecified type of carcinoma in situ of left breast |
| D060 | Carcinoma in situ of endocervix |
| D061 | Carcinoma in situ of exocervix |
| D067 | Carcinoma in situ of other parts of cervix |
| D069 | Carcinoma in situ of cervix, unspecified |
| D070 | Carcinoma in situ of endometrium |
| D071 | Carcinoma in situ of vulva |
| D072 | Carcinoma in situ of vagina |
| D0730 | Carcinoma in situ of unspecified female genital organs |
| D0739 | Carcinoma in situ of other female genital organs |
| D074 | Carcinoma in situ of penis |
| D075 | Carcinoma in situ of prostate |
| D0760 | Carcinoma in situ of unspecified male genital organs |
| D0761 | Carcinoma in situ of scrotum |
| D0769 | Carcinoma in situ of other male genital organs |
| D090 | Carcinoma in situ of bladder |
| D0910 | Carcinoma in situ of unspecified urinary organ |
| D0919 | Carcinoma in situ of other urinary organs |
| D0920 | Carcinoma in situ of unspecified eye |
| D0921 | Carcinoma in situ of right eye |
| D0922 | Carcinoma in situ of left eye |
| D093 | Carcinoma in situ of thyroid and other endocrine glands |
| D098 | Carcinoma in situ of other specified sites |
| D099 | Carcinoma in situ, unspecified |
| D100 | Benign neoplasm of lip |
| D101 | Benign neoplasm of tongue |
| D102 | Benign neoplasm of floor of mouth |
| D1030 | Benign neoplasm of unspecified part of mouth |
| D1039 | Benign neoplasm of other parts of mouth |
| D104 | Benign neoplasm of tonsil |
| D105 | Benign neoplasm of other parts of oropharynx |
| D106 | Benign neoplasm of nasopharynx |
| D107 | Benign neoplasm of hypopharynx |
| D109 | Benign neoplasm of pharynx, unspecified |
| D110 | Benign neoplasm of parotid gland |
| D117 | Benign neoplasm of other major salivary glands |
| D119 | Benign neoplasm of major salivary gland, unspecified |
| D120 | Benign neoplasm of cecum |
| D121 | Benign neoplasm of appendix |
| D122 | Benign neoplasm of ascending colon |
| D123 | Benign neoplasm of transverse colon |
| D124 | Benign neoplasm of descending colon |
| D125 | Benign neoplasm of sigmoid colon |
| D126 | Benign neoplasm of colon, unspecified |
| D127 | Benign neoplasm of rectosigmoid junction |
| D128 | Benign neoplasm of rectum |
| D129 | Benign neoplasm of anus and anal canal |
| D130 | Benign neoplasm of esophagus |
| D131 | Benign neoplasm of stomach |
| D132 | Benign neoplasm of duodenum |
| D1330 | Benign neoplasm of unspecified part of small intestine |
| D1339 | Benign neoplasm of other parts of small intestine |
| D134 | Benign neoplasm of liver |
| D135 | Benign neoplasm of extrahepatic bile ducts |
| D136 | Benign neoplasm of pancreas |
| D137 | Benign neoplasm of endocrine pancreas |
| D139 | Benign neoplasm of ill-defined sites within the digestive system |
| D140 | Benign neoplasm of middle ear, nasal cavity and accessory sinuses |
| D141 | Benign neoplasm of larynx |
| D142 | Benign neoplasm of trachea |
| D1430 | Benign neoplasm of unspecified bronchus and lung |
| D1431 | Benign neoplasm of right bronchus and lung |
| D1432 | Benign neoplasm of left bronchus and lung |
| D144 | Benign neoplasm of respiratory system, unspecified |
| D150 | Benign neoplasm of thymus |
| D151 | Benign neoplasm of heart |
| D152 | Benign neoplasm of mediastinum |
| D157 | Benign neoplasm of other specified intrathoracic organs |
| D159 | Benign neoplasm of intrathoracic organ, unspecified |
| D1600 | Benign neoplasm of scapula and long bones of unspecified upper limb |
| D1601 | Benign neoplasm of scapula and long bones of right upper limb |
| D1602 | Benign neoplasm of scapula and long bones of left upper limb |
| D1610 | Benign neoplasm of short bones of unspecified upper limb |
| D1611 | Benign neoplasm of short bones of right upper limb |
| D1612 | Benign neoplasm of short bones of left upper limb |
| D1620 | Benign neoplasm of long bones of unspecified lower limb |
| D1621 | Benign neoplasm of long bones of right lower limb |
| D1622 | Benign neoplasm of long bones of left lower limb |
| D1630 | Benign neoplasm of short bones of unspecified lower limb |
| D1631 | Benign neoplasm of short bones of right lower limb |
| D1632 | Benign neoplasm of short bones of left lower limb |
| D164 | Benign neoplasm of bones of skull and face |
| D165 | Benign neoplasm of lower jaw bone |
| D166 | Benign neoplasm of vertebral column |
| D167 | Benign neoplasm of ribs, sternum and clavicle |
| D168 | Benign neoplasm of pelvic bones, sacrum and coccyx |
| D169 | Benign neoplasm of bone and articular cartilage, unspecified |
| D170 | Benign lipomatous neoplasm of skin and subcutaneous tissue of head, face and neck |
| D171 | Benign lipomatous neoplasm of skin and subcutaneous tissue of trunk |
| D1720 | Benign lipomatous neoplasm of skin and subcutaneous tissue of unspecified limb |
| D1721 | Benign lipomatous neoplasm of skin and subcutaneous tissue of right arm |
| D1722 | Benign lipomatous neoplasm of skin and subcutaneous tissue of left arm |
| D1723 | Benign lipomatous neoplasm of skin and subcutaneous tissue of right leg |
| D1724 | Benign lipomatous neoplasm of skin and subcutaneous tissue of left leg |
| D1730 | Benign lipomatous neoplasm of skin and subcutaneous tissue of unspecified sites |
| D1739 | Benign lipomatous neoplasm of skin and subcutaneous tissue of other sites |
| D174 | Benign lipomatous neoplasm of intrathoracic organs |
| D175 | Benign lipomatous neoplasm of intra-abdominal organs |
| D176 | Benign lipomatous neoplasm of spermatic cord |
| D1771 | Benign lipomatous neoplasm of kidney |
| D1772 | Benign lipomatous neoplasm of other genitourinary organ |
| D1779 | Benign lipomatous neoplasm of other sites |
| D179 | Benign lipomatous neoplasm, unspecified |
| D1800 | Hemangioma unspecified site |
| D1801 | Hemangioma of skin and subcutaneous tissue |
| D1802 | Hemangioma of intracranial structures |
| D1803 | Hemangioma of intra-abdominal structures |
| D1809 | Hemangioma of other sites |
| D181 | Lymphangioma, any site |
| D190 | Benign neoplasm of mesothelial tissue of pleura |
| D191 | Benign neoplasm of mesothelial tissue of peritoneum |
| D197 | Benign neoplasm of mesothelial tissue of other sites |
| D199 | Benign neoplasm of mesothelial tissue, unspecified |
| D200 | Benign neoplasm of soft tissue of retroperitoneum |
| D201 | Benign neoplasm of soft tissue of peritoneum |
| D210 | Benign neoplasm of connective and other soft tissue of head, face and neck |
| D2110 | Benign neoplasm of connective and other soft tissue of unspecified upper limb, including shoulder |
| D2111 | Benign neoplasm of connective and other soft tissue of right upper limb, including shoulder |
| D2112 | Benign neoplasm of connective and other soft tissue of left upper limb, including shoulder |
| D2120 | Benign neoplasm of connective and other soft tissue of unspecified lower limb, including hip |
| D2121 | Benign neoplasm of connective and other soft tissue of right lower limb, including hip |
| D2122 | Benign neoplasm of connective and other soft tissue of left lower limb, including hip |
| D213 | Benign neoplasm of connective and other soft tissue of thorax |
| D214 | Benign neoplasm of connective and other soft tissue of abdomen |
| D215 | Benign neoplasm of connective and other soft tissue of pelvis |
| D216 | Benign neoplasm of connective and other soft tissue of trunk, unspecified |
| D219 | Benign neoplasm of connective and other soft tissue, unspecified |
| D220 | Melanocytic nevi of lip |
| D2210 | Melanocytic nevi of unspecified eyelid, including canthus |
| D22111 | Melanocytic nevi of right upper eyelid, including canthus |
| D22112 | Melanocytic nevi of right lower eyelid, including canthus |
| D22121 | Melanocytic nevi of left upper eyelid, including canthus |
| D22122 | Melanocytic nevi of left lower eyelid, including canthus |
| D2220 | Melanocytic nevi of unspecified ear and external auricular canal |
| D2221 | Melanocytic nevi of right ear and external auricular canal |
| D2222 | Melanocytic nevi of left ear and external auricular canal |
| D2230 | Melanocytic nevi of unspecified part of face |
| D2239 | Melanocytic nevi of other parts of face |
| D224 | Melanocytic nevi of scalp and neck |
| D225 | Melanocytic nevi of trunk |
| D2260 | Melanocytic nevi of unspecified upper limb, including shoulder |
| D2261 | Melanocytic nevi of right upper limb, including shoulder |
| D2262 | Melanocytic nevi of left upper limb, including shoulder |
| D2270 | Melanocytic nevi of unspecified lower limb, including hip |
| D2271 | Melanocytic nevi of right lower limb, including hip |
| D2272 | Melanocytic nevi of left lower limb, including hip |
| D229 | Melanocytic nevi, unspecified |
| D230 | Other benign neoplasm of skin of lip |
| D2310 | Other benign neoplasm of skin of unspecified eyelid, including canthus |
| D23111 | Other benign neoplasm of skin of right upper eyelid, including canthus |
| D23112 | Other benign neoplasm of skin of right lower eyelid, including canthus |
| D23121 | Other benign neoplasm of skin of left upper eyelid, including canthus |
| D23122 | Other benign neoplasm of skin of left lower eyelid, including canthus |
| D2320 | Other benign neoplasm of skin of unspecified ear and external auricular canal |
| D2321 | Other benign neoplasm of skin of right ear and external auricular canal |
| D2322 | Other benign neoplasm of skin of left ear and external auricular canal |
| D2330 | Other benign neoplasm of skin of unspecified part of face |
| D2339 | Other benign neoplasm of skin of other parts of face |
| D234 | Other benign neoplasm of skin of scalp and neck |
| D235 | Other benign neoplasm of skin of trunk |
| D2360 | Other benign neoplasm of skin of unspecified upper limb, including shoulder |
| D2361 | Other benign neoplasm of skin of right upper limb, including shoulder |
| D2362 | Other benign neoplasm of skin of left upper limb, including shoulder |
| D2370 | Other benign neoplasm of skin of unspecified lower limb, including hip |
| D2371 | Other benign neoplasm of skin of right lower limb, including hip |
| D2372 | Other benign neoplasm of skin of left lower limb, including hip |
| D239 | Other benign neoplasm of skin, unspecified |
| D241 | Benign neoplasm of right breast |
| D242 | Benign neoplasm of left breast |
| D249 | Benign neoplasm of unspecified breast |
| D250 | Submucous leiomyoma of uterus |
| D251 | Intramural leiomyoma of uterus |
| D252 | Subserosal leiomyoma of uterus |
| D259 | Leiomyoma of uterus, unspecified |
| D260 | Other benign neoplasm of cervix uteri |
| D261 | Other benign neoplasm of corpus uteri |
| D267 | Other benign neoplasm of other parts of uterus |
| D269 | Other benign neoplasm of uterus, unspecified |
| D270 | Benign neoplasm of right ovary |
| D271 | Benign neoplasm of left ovary |
| D279 | Benign neoplasm of unspecified ovary |
| D280 | Benign neoplasm of vulva |
| D281 | Benign neoplasm of vagina |
| D282 | Benign neoplasm of uterine tubes and ligaments |
| D287 | Benign neoplasm of other specified female genital organs |
| D289 | Benign neoplasm of female genital organ, unspecified |
| D290 | Benign neoplasm of penis |
| D291 | Benign neoplasm of prostate |
| D2920 | Benign neoplasm of unspecified testis |
| D2921 | Benign neoplasm of right testis |
| D2922 | Benign neoplasm of left testis |
| D2930 | Benign neoplasm of unspecified epididymis |
| D2931 | Benign neoplasm of right epididymis |
| D2932 | Benign neoplasm of left epididymis |
| D294 | Benign neoplasm of scrotum |
| D298 | Benign neoplasm of other specified male genital organs |
| D299 | Benign neoplasm of male genital organ, unspecified |
| D3000 | Benign neoplasm of unspecified kidney |
| D3001 | Benign neoplasm of right kidney |
| D3002 | Benign neoplasm of left kidney |
| D3010 | Benign neoplasm of unspecified renal pelvis |
| D3011 | Benign neoplasm of right renal pelvis |
| D3012 | Benign neoplasm of left renal pelvis |
| D3020 | Benign neoplasm of unspecified ureter |
| D3021 | Benign neoplasm of right ureter |
| D3022 | Benign neoplasm of left ureter |
| D303 | Benign neoplasm of bladder |
| D304 | Benign neoplasm of urethra |
| D308 | Benign neoplasm of other specified urinary organs |
| D309 | Benign neoplasm of urinary organ, unspecified |
| D3100 | Benign neoplasm of unspecified conjunctiva |
| D3101 | Benign neoplasm of right conjunctiva |
| D3102 | Benign neoplasm of left conjunctiva |
| D3110 | Benign neoplasm of unspecified cornea |
| D3111 | Benign neoplasm of right cornea |
| D3112 | Benign neoplasm of left cornea |
| D3120 | Benign neoplasm of unspecified retina |
| D3121 | Benign neoplasm of right retina |
| D3122 | Benign neoplasm of left retina |
| D3130 | Benign neoplasm of unspecified choroid |
| D3131 | Benign neoplasm of right choroid |
| D3132 | Benign neoplasm of left choroid |
| D3140 | Benign neoplasm of unspecified ciliary body |
| D3141 | Benign neoplasm of right ciliary body |
| D3142 | Benign neoplasm of left ciliary body |
| D3150 | Benign neoplasm of unspecified lacrimal gland and duct |
| D3151 | Benign neoplasm of right lacrimal gland and duct |
| D3152 | Benign neoplasm of left lacrimal gland and duct |
| D3160 | Benign neoplasm of unspecified site of unspecified orbit |
| D3161 | Benign neoplasm of unspecified site of right orbit |
| D3162 | Benign neoplasm of unspecified site of left orbit |
| D3190 | Benign neoplasm of unspecified part of unspecified eye |
| D3191 | Benign neoplasm of unspecified part of right eye |
| D3192 | Benign neoplasm of unspecified part of left eye |
| D320 | Benign neoplasm of cerebral meninges |
| D321 | Benign neoplasm of spinal meninges |
| D329 | Benign neoplasm of meninges, unspecified |
| D330 | Benign neoplasm of brain, supratentorial |
| D331 | Benign neoplasm of brain, infratentorial |
| D332 | Benign neoplasm of brain, unspecified |
| D333 | Benign neoplasm of cranial nerves |
| D334 | Benign neoplasm of spinal cord |
| D337 | Benign neoplasm of other specified parts of central nervous system |
| D339 | Benign neoplasm of central nervous system, unspecified |
| D34 | Benign neoplasm of thyroid gland |
| D3500 | Benign neoplasm of unspecified adrenal gland |
| D3501 | Benign neoplasm of right adrenal gland |
| D3502 | Benign neoplasm of left adrenal gland |
| D351 | Benign neoplasm of parathyroid gland |
| D352 | Benign neoplasm of pituitary gland |
| D353 | Benign neoplasm of craniopharyngeal duct |
| D354 | Benign neoplasm of pineal gland |
| D355 | Benign neoplasm of carotid body |
| D356 | Benign neoplasm of aortic body and other paraganglia |
| D357 | Benign neoplasm of other specified endocrine glands |
| D359 | Benign neoplasm of endocrine gland, unspecified |
| D360 | Benign neoplasm of lymph nodes |
| D3610 | Benign neoplasm of peripheral nerves and autonomic nervous system, unspecified |
| D3611 | Benign neoplasm of peripheral nerves and autonomic nervous system of face, head, and neck |
| D3612 | Benign neoplasm of peripheral nerves and autonomic nervous system, upper limb, including shoulder |
| D3613 | Benign neoplasm of peripheral nerves and autonomic nervous system of lower limb, including hip |
| D3614 | Benign neoplasm of peripheral nerves and autonomic nervous system of thorax |
| D3615 | Benign neoplasm of peripheral nerves and autonomic nervous system of abdomen |
| D3616 | Benign neoplasm of peripheral nerves and autonomic nervous system of pelvis |
| D3617 | Benign neoplasm of peripheral nerves and autonomic nervous system of trunk, unspecified |
| D367 | Benign neoplasm of other specified sites |
| D369 | Benign neoplasm, unspecified site |
| D3701 | Neoplasm of uncertain behavior of lip |
| D3702 | Neoplasm of uncertain behavior of tongue |
| D37030 | Neoplasm of uncertain behavior of the parotid salivary glands |
| D37031 | Neoplasm of uncertain behavior of the sublingual salivary glands |
| D37032 | Neoplasm of uncertain behavior of the submandibular salivary glands |
| D37039 | Neoplasm of uncertain behavior of the major salivary glands, unspecified |
| D3704 | Neoplasm of uncertain behavior of the minor salivary glands |
| D3705 | Neoplasm of uncertain behavior of pharynx |
| D3709 | Neoplasm of uncertain behavior of other specified sites of the oral cavity |
| D371 | Neoplasm of uncertain behavior of stomach |
| D372 | Neoplasm of uncertain behavior of small intestine |
| D373 | Neoplasm of uncertain behavior of appendix |
| D374 | Neoplasm of uncertain behavior of colon |
| D375 | Neoplasm of uncertain behavior of rectum |
| D376 | Neoplasm of uncertain behavior of liver, gallbladder and bile ducts |
| D378 | Neoplasm of uncertain behavior of other specified digestive organs |
| D379 | Neoplasm of uncertain behavior of digestive organ, unspecified |
| D380 | Neoplasm of uncertain behavior of larynx |
| D381 | Neoplasm of uncertain behavior of trachea, bronchus and lung |
| D382 | Neoplasm of uncertain behavior of pleura |
| D383 | Neoplasm of uncertain behavior of mediastinum |
| D384 | Neoplasm of uncertain behavior of thymus |
| D385 | Neoplasm of uncertain behavior of other respiratory organs |
| D386 | Neoplasm of uncertain behavior of respiratory organ, unspecified |
| D390 | Neoplasm of uncertain behavior of uterus |
| D3910 | Neoplasm of uncertain behavior of unspecified ovary |
| D3911 | Neoplasm of uncertain behavior of right ovary |
| D3912 | Neoplasm of uncertain behavior of left ovary |
| D392 | Neoplasm of uncertain behavior of placenta |
| D398 | Neoplasm of uncertain behavior of other specified female genital organs |
| D399 | Neoplasm of uncertain behavior of female genital organ, unspecified |
| D3A00 | Benign carcinoid tumor of unspecified site |
| D3A010 | Benign carcinoid tumor of the duodenum |
| D3A011 | Benign carcinoid tumor of the jejunum |
| D3A012 | Benign carcinoid tumor of the ileum |
| D3A019 | Benign carcinoid tumor of the small intestine, unspecified portion |
| D3A020 | Benign carcinoid tumor of the appendix |
| D3A021 | Benign carcinoid tumor of the cecum |
| D3A022 | Benign carcinoid tumor of the ascending colon |
| D3A023 | Benign carcinoid tumor of the transverse colon |
| D3A024 | Benign carcinoid tumor of the descending colon |
| D3A025 | Benign carcinoid tumor of the sigmoid colon |
| D3A026 | Benign carcinoid tumor of the rectum |
| D3A029 | Benign carcinoid tumor of the large intestine, unspecified portion |
| D3A090 | Benign carcinoid tumor of the bronchus and lung |
| D3A091 | Benign carcinoid tumor of the thymus |
| D3A092 | Benign carcinoid tumor of the stomach |
| D3A093 | Benign carcinoid tumor of the kidney |
| D3A094 | Benign carcinoid tumor of the foregut, unspecified |
| D3A095 | Benign carcinoid tumor of the midgut, unspecified |
| D3A096 | Benign carcinoid tumor of the hindgut, unspecified |
| D3A098 | Benign carcinoid tumors of other sites |
| D3A8 | Other benign neuroendocrine tumors |
| D400 | Neoplasm of uncertain behavior of prostate |
| D4010 | Neoplasm of uncertain behavior of unspecified testis |
| D4011 | Neoplasm of uncertain behavior of right testis |
| D4012 | Neoplasm of uncertain behavior of left testis |
| D408 | Neoplasm of uncertain behavior of other specified male genital organs |
| D409 | Neoplasm of uncertain behavior of male genital organ, unspecified |
| D4100 | Neoplasm of uncertain behavior of unspecified kidney |
| D4101 | Neoplasm of uncertain behavior of right kidney |
| D4102 | Neoplasm of uncertain behavior of left kidney |
| D4110 | Neoplasm of uncertain behavior of unspecified renal pelvis |
| D4111 | Neoplasm of uncertain behavior of right renal pelvis |
| D4112 | Neoplasm of uncertain behavior of left renal pelvis |
| D4120 | Neoplasm of uncertain behavior of unspecified ureter |
| D4121 | Neoplasm of uncertain behavior of right ureter |
| D4122 | Neoplasm of uncertain behavior of left ureter |
| D413 | Neoplasm of uncertain behavior of urethra |
| D414 | Neoplasm of uncertain behavior of bladder |
| D418 | Neoplasm of uncertain behavior of other specified urinary organs |
| D419 | Neoplasm of uncertain behavior of unspecified urinary organ |
| D420 | Neoplasm of uncertain behavior of cerebral meninges |
| D421 | Neoplasm of uncertain behavior of spinal meninges |
| D429 | Neoplasm of uncertain behavior of meninges, unspecified |
| D430 | Neoplasm of uncertain behavior of brain, supratentorial |
| D431 | Neoplasm of uncertain behavior of brain, infratentorial |
| D432 | Neoplasm of uncertain behavior of brain, unspecified |
| D433 | Neoplasm of uncertain behavior of cranial nerves |
| D434 | Neoplasm of uncertain behavior of spinal cord |
| D438 | Neoplasm of uncertain behavior of other specified parts of central nervous system |
| D439 | Neoplasm of uncertain behavior of central nervous system, unspecified |
| D440 | Neoplasm of uncertain behavior of thyroid gland |
| D4410 | Neoplasm of uncertain behavior of unspecified adrenal gland |
| D4411 | Neoplasm of uncertain behavior of right adrenal gland |
| D4412 | Neoplasm of uncertain behavior of left adrenal gland |
| D442 | Neoplasm of uncertain behavior of parathyroid gland |
| D443 | Neoplasm of uncertain behavior of pituitary gland |
| D444 | Neoplasm of uncertain behavior of craniopharyngeal duct |
| D445 | Neoplasm of uncertain behavior of pineal gland |
| D446 | Neoplasm of uncertain behavior of carotid body |
| D447 | Neoplasm of uncertain behavior of aortic body and other paraganglia |
| D449 | Neoplasm of uncertain behavior of unspecified endocrine gland |
| D45 | Polycythemia vera |
| D460 | Refractory anemia without ring sideroblasts, so stated |
| D461 | Refractory anemia with ring sideroblasts |
| D4620 | Refractory anemia with excess of blasts, unspecified |
| D4621 | Refractory anemia with excess of blasts 1 |
| D4622 | Refractory anemia with excess of blasts 2 |
| D464 | Refractory anemia, unspecified |
| D469 | Myelodysplastic syndrome, unspecified |
| D46A | Refractory cytopenia with multilineage dysplasia |
| D46B | Refractory cytopenia with multilineage dysplasia and ring sideroblasts |
| D46C | Myelodysplastic syndrome with isolated del(5q) chromosomal abnormality |
| D46Z | Other myelodysplastic syndromes |
| D4701 | Cutaneous mastocytosis |
| D4702 | Systemic mastocytosis |
| D4709 | Other mast cell neoplasms of uncertain behavior |
| D471 | Chronic myeloproliferative disease |
| D472 | Monoclonal gammopathy |
| D473 | Essential (hemorrhagic) thrombocythemia |
| D474 | Osteomyelofibrosis |
| D479 | Neoplasm of uncertain behavior of lymphoid, hematopoietic and related tissue, unspecified |
| D47Z1 | Post-transplant lymphoproliferative disorder (PTLD) |
| D47Z2 | Castleman disease |
| D47Z9 | Other specified neoplasms of uncertain behavior of lymphoid, hematopoietic and related tissue |
| D480 | Neoplasm of uncertain behavior of bone and articular cartilage |
| D481 | Neoplasm of uncertain behavior of connective and other soft tissue |
| D482 | Neoplasm of uncertain behavior of peripheral nerves and autonomic nervous system |
| D483 | Neoplasm of uncertain behavior of retroperitoneum |
| D484 | Neoplasm of uncertain behavior of peritoneum |
| D485 | Neoplasm of uncertain behavior of skin |
| D4860 | Neoplasm of uncertain behavior of unspecified breast |
| D4861 | Neoplasm of uncertain behavior of right breast |
| D4862 | Neoplasm of uncertain behavior of left breast |
| D487 | Neoplasm of uncertain behavior of other specified sites |
| D489 | Neoplasm of uncertain behavior, unspecified |
| D490 | Neoplasm of unspecified behavior of digestive system |
| D491 | Neoplasm of unspecified behavior of respiratory system |
| D492 | Neoplasm of unspecified behavior of bone, soft tissue, and skin |
| D493 | Neoplasm of unspecified behavior of breast |
| D494 | Neoplasm of unspecified behavior of bladder |
| D49511 | Neoplasm of unspecified behavior of right kidney |
| D49512 | Neoplasm of unspecified behavior of left kidney |
| D49519 | Neoplasm of unspecified behavior of unspecified kidney |
| D4959 | Neoplasm of unspecified behavior of other genitourinary organ |
| D496 | Neoplasm of unspecified behavior of brain |
| D497 | Neoplasm of unspecified behavior of endocrine glands and other parts of nervous system |
| D4981 | Neoplasm of unspecified behavior of retina and choroid |
| D4989 | Neoplasm of unspecified behavior of other specified sites |
| D499 | Neoplasm of unspecified behavior of unspecified site |

**Supplemental Table 4 – Immunocompromised-Related Diagnostic Codes**

| **Immunocompromised-Related ICD-9 Codes** | |
| --- | --- |
| 42 | Human immunodeficiency virus [HIV] disease |
| 74 | Cryptosporidiosis |
| 135 | Sarcoidosis |
| 785 | Cytomegaloviral disease |
| 1000 | Primary tuberculous infection - unspecified |
| 1001 | Primary tuberculous infection - bacteriological or histological examination not done |
| 1002 | Primary tuberculous infection - bacteriological or histological examination unknown (at present) |
| 1003 | Primary tuberculous infection - tubercle bacilli found (in sputum) by microscopy |
| 1004 | Primary tuberculous infection - tubercle bacilli not found (in sputum) by microscopy - but found by bacterial culture |
| 1005 | Primary tuberculous infection - tubercle bacilli not found by bacteriological examination - but tuberculosis confirmed histologically |
| 1006 | Primary tuberculous infection - tubercle bacilli not found by bacteriological or histological examination - but tuberculosis confirmed by other methods [inoculation of animals] |
| 1010 | Tuberculous pleurisy in primary progressive tuberculosis - unspecified |
| 1011 | Tuberculous pleurisy in primary progressive tuberculosis - bacteriological or histological examination not done |
| 1012 | Tuberculous pleurisy in primary progressive tuberculosis - bacteriological or histological examination unknown (at present) |
| 1013 | Tuberculous pleurisy in primary progressive tuberculosis - tubercle bacilli found (in sputum) by microscopy |
| 1014 | Tuberculous pleurisy in primary progressive tuberculosis - tubercle bacilli not found (in sputum) by microscopy - but found by bacterial culture |
| 1015 | Tuberculous pleurisy in primary progressive tuberculosis - tubercle bacilli not found by bacteriological examination - but tuberculosis confirmed histologically |
| 1016 | Tuberculous pleurisy in primary progressive tuberculosis - tubercle bacilli not found by bacteriological or histological examination - but tuberculosis confirmed by other methods [inoculation of animals] |
| 1080 | Other primary progressive tuberculosis - unspecified |
| 1081 | Other primary progressive tuberculosis - bacteriological or histological examination not done |
| 1082 | Other primary progressive tuberculosis - bacteriological or histological examination unknown (at present) |
| 1083 | Other primary progressive tuberculosis - tubercle bacilli found (in sputum) by microscopy |
| 1084 | Other primary progressive tuberculosis - tubercle bacilli not found (in sputum) by microscopy - but found by bacterial culture |
| 1085 | Other primary progressive tuberculosis - tubercle bacilli not found by bacteriological examination - but tuberculosis confirmed histologically |
| 1086 | Other primary progressive tuberculosis - tubercle bacilli not found by bacteriological or histological examination - but tuberculosis confirmed by other methods [inoculation of animals] |
| 1090 | Primary tuberculous infection - unspecified - unspecified |
| 1091 | Primary tuberculous infection - unspecified - bacteriological or histological examination not done |
| 1092 | Primary tuberculous infection - unspecified - bacteriological or histological examination unknown (at present) |
| 1093 | Primary tuberculous infection - unspecified - tubercle bacilli found (in sputum) by microscopy |
| 1094 | Primary tuberculous infection - unspecified - tubercle bacilli not found (in sputum) by microscopy - but found by bacterial culture |
| 1095 | Primary tuberculous infection - unspecified - tubercle bacilli not found by bacteriological examination - but tuberculosis confirmed histologically |
| 1096 | Primary tuberculous infection - unspecified - tubercle bacilli not found by bacteriological or histological examination - but tuberculosis confirmed by other methods [inoculation of animals] |
| 1100 | Tuberculosis of lung - infiltrative - unspecified |
| 1101 | Tuberculosis of lung - infiltrative - bacteriological or histological examination not done |
| 1102 | Tuberculosis of lung - infiltrative - bacteriological or histological examination unknown (at present) |
| 1103 | Tuberculosis of lung - infiltrative - tubercle bacilli found (in sputum) by microscopy |
| 1104 | Tuberculosis of lung - infiltrative - tubercle bacilli not found (in sputum) by microscopy - but found by bacterial culture |
| 1105 | Tuberculosis of lung - infiltrative - tubercle bacilli not found by bacteriological examination - but tuberculosis confirmed histologically |
| 1106 | Tuberculosis of lung - infiltrative - tubercle bacilli not found bacteriological or histological examination - but tuberculosis confirmed by other methods [inoculation of animals] |
| 1110 | Tuberculosis of lung - nodular - unspecified |
| 1111 | Tuberculosis of lung - nodular - bacteriological or histological examination not done |
| 1112 | Tuberculosis of lung - nodular - bacteriological or histological examination unknown (at present) |
| 1113 | Tuberculosis of lung - nodular - tubercle bacilli found (in sputum) by microscopy |
| 1114 | Tuberculosis of lung - nodular - tubercle bacilli not found (in sputum) by microscopy - but found by bacterial culture |
| 1115 | Tuberculosis of lung - nodular - tubercle bacilli not found by bacteriological examination - but tuberculosis confirmed histologically |
| 1116 | Tuberculosis of lung - nodular - tubercle bacilli not found by bacteriological or histological examination - but tuberculosis confirmed by other methods [inoculation of animals] |
| 1120 | Tuberculosis of lung with cavitation - unspecified |
| 1121 | Tuberculosis of lung with cavitation - bacteriological or histological examination not done |
| 1122 | Tuberculosis of lung with cavitation - bacteriological or histological examination unknown (at present) |
| 1123 | Tuberculosis of lung with cavitation - tubercle bacilli found (in sputum) by microscopy |
| 1124 | Tuberculosis of lung with cavitation - tubercle bacilli not found (in sputum) by microscopy - but found by bacterial culture |
| 1125 | Tuberculosis of lung with cavitation - tubercle bacilli not found by bacteriological examination - but tuberculosis confirmed histologically |
| 1126 | Tuberculosis of lung with cavitation - tubercle bacilli not found by bacteriological or histological examination - but tuberculosis confirmed by other methods [inoculation of animals] |
| 1129 | Candidiasis of unspecified site |
| 1130 | Tuberculosis of bronchus - unspecified |
| 1131 | Tuberculosis of bronchus - bacteriological or histological examination not done |
| 1132 | Tuberculosis of bronchus - bacteriological or histological examination unknown (at present) |
| 1133 | Tuberculosis of bronchus - tubercle bacilli found (in sputum) by microscopy |
| 1134 | Tuberculosis of bronchus - tubercle bacilli not found (in sputum) by microscopy - but found in bacterial culture |
| 1135 | Tuberculosis of bronchus - tubercle bacilli not found by bacteriological examination - but tuberculosis confirmed histologically |
| 1136 | Tuberculosis of bronchus - tubercle bacilli not found by bacteriological or histological examination - but tuberculosis confirmed by other methods [inoculation of animals] |
| 1140 | Tuberculous fibrosis of lung - unspecified |
| 1141 | Tuberculous fibrosis of lung - bacteriological or histological examination not done |
| 1142 | Tuberculous fibrosis of lung - bacteriological or histological examination unknown (at present) |
| 1143 | Tuberculous fibrosis of lung - tubercle bacilli found (in sputum) by microscopy |
| 1144 | Tuberculous fibrosis of lung - tubercle bacilli not found (in sputum) by microscopy - but found by bacterial culture |
| 1145 | Tuberculous fibrosis of lung - tubercle bacilli not found by bacteriological examination - but tuberculosis confirmed histologically |
| 1146 | Tuberculous fibrosis of lung - tubercle bacilli not found by bacteriological or histological examination - but tuberculosis confirmed by other methods [inoculation of animals] |
| 1150 | Tuberculous bronchiectasis - unspecified |
| 1151 | Tuberculous bronchiectasis - bacteriological or histological examination not done |
| 1152 | Tuberculous bronchiectasis - bacteriological or histological examination unknown (at present) |
| 1153 | Tuberculous bronchiectasis - tubercle bacilli found (in sputum) by microscopy |
| 1154 | Tuberculous bronchiectasis - tubercle bacilli not found (in sputum) by microscopy - but found by bacterial culture |
| 1155 | Tuberculous bronchiectasis - tubercle bacilli not found by bacteriological examination - but tuberculosis confirmed histologically |
| 1156 | Tuberculous bronchiectasis - tubercle bacilli not found by bacteriological or histological examination - but tuberculosis confirmed by other methods [inoculation of animals] |
| 1160 | Tuberculous pneumonia [any form] - unspecified |
| 1161 | Tuberculous pneumonia [any form] - bacteriological or histological examination not done |
| 1162 | Tuberculous pneumonia [any form] - bacteriological or histological examination unknown (at present) |
| 1163 | Tuberculous pneumonia [any form] - tubercle bacilli found (in sputum) by microscopy |
| 1164 | Tuberculous pneumonia [any form] - tubercle bacilli not found (in sputum) by microscopy - but found by bacterial culture |
| 1165 | Tuberculous pneumonia [any form] - tubercle bacilli not found by bacteriological examination - but tuberculosis confirmed histologically |
| 1166 | Tuberculous pneumonia [any form] - tubercle bacilli not found by bacteriological or histological examination - but tuberculosis confirmed by other methods [inoculation of animals] |
| 1170 | Tuberculous pneumothorax - unspecified |
| 1171 | Tuberculous pneumothorax - bacteriological or histological examination not done |
| 1172 | Tuberculous pneumothorax - bacteriological or histological examination unknown (at present) |
| 1173 | Tuberculous pneumothorax - tubercle bacilli found (in sputum) by microscopy |
| 1173 | Aspergillosis |
| 1174 | Tuberculous pneumothorax - tubercle bacilli not found (in sputum) by microscopy - but found by bacterial culture |
| 1175 | Tuberculous pneumothorax - tubercle bacilli not found by bacteriological examination - but tuberculosis confirmed histologically |
| 1175 | Cryptococcosis |
| 1176 | Tuberculous pneumothorax - tubercle bacilli not found by bacteriological or histological examination - but tuberculosis confirmed by other methods [inoculation of animals] |
| 1180 | Other specified pulmonary tuberculosis - unspecified |
| 1181 | Other specified pulmonary tuberculosis - bacteriological or histological examination not done |
| 1182 | Other specified pulmonary tuberculosis - bacteriological or histological examination unknown (at present) |
| 1183 | Other specified pulmonary tuberculosis - tubercle bacilli found (in sputum) by microscopy |
| 1184 | Other specified pulmonary tuberculosis - tubercle bacilli not found (in sputum) by microscopy - but found by bacterial culture |
| 1185 | Other specified pulmonary tuberculosis - tubercle bacilli not found by bacteriological examination - but tuberculosis confirmed histologically |
| 1186 | Other specified pulmonary tuberculosis - tubercle bacilli not found by bacteriological or histological examination - but tuberculosis confirmed by other methods [inoculation of animals] |
| 1190 | Pulmonary tuberculosis - unspecified - unspecified |
| 1191 | Pulmonary tuberculosis - unspecified - bacteriological or histological examination not done |
| 1192 | Pulmonary tuberculosis - unspecified - bacteriological or histological examination unknown (at present) |
| 1193 | Pulmonary tuberculosis - unspecified - tubercle bacilli found (in sputum) by microscopy |
| 1194 | Pulmonary tuberculosis - unspecified - tubercle bacilli not found (in sputum) by microscopy - but found by bacterial culture |
| 1195 | Pulmonary tuberculosis - unspecified - tubercle bacilli not found by bacteriological examination - but tuberculosis confirmed histologically |
| 1196 | Pulmonary tuberculosis - unspecified - tubercle bacilli not found by bacteriological or histological examination - but tuberculosis confirmed by other methods [inoculation of animals] |
| 1200 | Tuberculous pleurisy - unspecified |
| 1201 | Tuberculous pleurisy - bacteriological or histological examination not done |
| 1202 | Tuberculous pleurisy - bacteriological or histological examination unknown (at present) |
| 1203 | Tuberculous pleurisy - tubercle bacilli found (in sputum) by microscopy |
| 1204 | Tuberculous pleurisy - tubercle bacilli not found (in sputum) by microscopy - but found by bacterial culture |
| 1205 | Tuberculous pleurisy - tubercle bacilli not found by bacteriological examination - but tuberculosis confirmed histologically |
| 1206 | Tuberculous pleurisy - tubercle bacilli not found by bacteriological or histological examination - but tuberculosis confirmed by other methods [inoculation of animals] |
| 1210 | Tuberculosis of intrathoracic lymph nodes - unspecified |
| 1211 | Tuberculosis of intrathoracic lymph nodes - bacteriological or histological examination not done |
| 1212 | Tuberculosis of intrathoracic lymph nodes - bacteriological or histological examination unknown (at present) |
| 1213 | Tuberculosis of intrathoracic lymph nodes - tubercle bacilli found (in sputum) by microscopy |
| 1214 | Tuberculosis of intrathoracic lymph nodes - tubercle bacilli not found (in sputum) by microscopy - but found by bacterial culture |
| 1215 | Tuberculosis of intrathoracic lymph nodes - tubercle bacilli not found by bacteriological examination - but tuberculosis confirmed histologically |
| 1216 | Tuberculosis of intrathoracic lymph nodes - tubercle bacilli not found by bacteriological or histological examination - but tuberculosis confirmed by other methods [inoculation of animals] |
| 1220 | Isolated tracheal or bronchial tuberculosis - unspecified |
| 1221 | Isolated tracheal or bronchial tuberculosis - bacteriological or histological examination not done |
| 1222 | Isolated tracheal or bronchial tuberculosis - bacteriological or histological examination unknown (at present) |
| 1223 | Isolated tracheal or bronchial tuberculosis - tubercle bacilli found (in sputum) by microscopy |
| 1224 | Isolated tracheal or bronchial tuberculosis - tubercle bacilli not found (in sputum) by microscopy - but found by bacterial culture |
| 1225 | Isolated tracheal or bronchial tuberculosis - tubercle bacilli not found by bacteriological examination - but tuberculosis confirmed histologically |
| 1226 | Isolated tracheal or bronchial tuberculosis - tubercle bacilli not found by bacteriological or histological examination - but tuberculosis confirmed by other methods [inoculation of animals] |
| 1230 | Tuberculous laryngitis - unspecified |
| 1231 | Tuberculous laryngitis - bacteriological or histological examination not done |
| 1232 | Tuberculous laryngitis - bacteriological or histological examination unknown (at present) |
| 1233 | Tuberculous laryngitis - tubercle bacilli found (in sputum) by microscopy |
| 1234 | Tuberculous laryngitis - tubercle bacilli not found (in sputum) by microscopy - but found by bacterial culture |
| 1235 | Tuberculous laryngitis - tubercle bacilli not found by bacteriological examination - but tuberculosis confirmed histologically |
| 1236 | Tuberculous laryngitis - tubercle bacilli not found by bacteriological or histological examination - but tuberculosis confirmed by other methods [inoculation of animals] |
| 1280 | Other specified respiratory tuberculosis - unspecified |
| 1281 | Other specified respiratory tuberculosis - bacteriological or histological examination not done |
| 1282 | Other specified respiratory tuberculosis - bacteriological or histological examination unknown (at present) |
| 1283 | Other specified respiratory tuberculosis - tubercle bacilli found (in sputum) by microscopy |
| 1284 | Other specified respiratory tuberculosis - tubercle bacilli not found (in sputum) by microscopy - but found by bacterial culture |
| 1285 | Other specified respiratory tuberculosis - tubercle bacilli not found by bacteriological examination - but tuberculosis confirmed histologically |
| 1286 | Other specified respiratory tuberculosis - tubercle bacilli not found by bacteriological or histological examination - but tuberculosis confirmed by other methods [inoculation of animals] |
| 1300 | Tuberculous meningitis - unspecified |
| 1300 | Meningoencephalitis due to toxoplasmosis |
| 1301 | Tuberculous meningitis - bacteriological or histological examination not done |
| 1301 | Conjunctivitis due to toxoplasmosis |
| 1302 | Tuberculous meningitis - bacteriological or histological examination unknown (at present) |
| 1302 | Chorioretinitis due to toxoplasmosis |
| 1303 | Tuberculous meningitis - tubercle bacilli found (in sputum) by microscopy |
| 1303 | Myocarditis due to toxoplasmosis |
| 1304 | Tuberculous meningitis - tubercle bacilli not found (in sputum) by microscopy - but found by bacterial culture |
| 1304 | Pneumonitis due to toxoplasmosis |
| 1305 | Tuberculous meningitis - tubercle bacilli not found by bacteriological examination - but tuberculosis confirmed histologically |
| 1305 | Hepatitis due to toxoplasmosis |
| 1306 | Tuberculous meningitis - tubercle bacilli not found by bacteriological or histological examination - but tuberculosis confirmed by other methods [inoculation of animals] |
| 1307 | Toxoplasmosis of other specified sites |
| 1308 | Multisystemic disseminated toxoplasmosis |
| 1309 | Toxoplasmosis - unspecified |
| 1310 | Tuberculoma of meninges - unspecified |
| 1311 | Tuberculoma of meninges - bacteriological or histological examination not done |
| 1312 | Tuberculoma of meninges - bacteriological or histological examination unknown (at present) |
| 1313 | Tuberculoma of meninges - tubercle bacilli found (in sputum) by microscopy |
| 1314 | Tuberculoma of meninges - tubercle bacilli not found (in sputum) by microscopy - but found by bacterial culture |
| 1315 | Tuberculoma of meninges - tubercle bacilli not found by bacteriological examination - but tuberculosis confirmed histologically |
| 1316 | Tuberculoma of meninges - tubercle bacilli not found by bacteriological or histological examination - but tuberculosis confirmed by other methods [inoculation of animals] |
| 1320 | Tuberculoma of brain - unspecified |
| 1321 | Tuberculoma of brain - bacteriological or histological examination not done |
| 1322 | Tuberculoma of brain - bacteriological or histological examination unknown (at present) |
| 1323 | Tuberculoma of brain - tubercle bacilli found (in sputum) by microscopy |
| 1324 | Tuberculoma of brain - tubercle bacilli not found (in sputum) by microscopy - but found by bacterial culture |
| 1325 | Tuberculoma of brain - tubercle bacilli not found by bacteriological examination - but tuberculosis confirmed histologically |
| 1326 | Tuberculoma of brain - tubercle bacilli not found by bacteriological or histological examination - but tuberculosis confirmed by other methods [inoculation of animals] |
| 1330 | Tuberculous abscess of brain - unspecified |
| 1331 | Tuberculous abscess of brain - bacteriological or histological examination not done |
| 1332 | Tuberculous abscess of brain - bacteriological or histological examination unknown (at present) |
| 1333 | Tuberculous abscess of brain - tubercle bacilli found (in sputum) by microscopy |
| 1334 | Tuberculous abscess of brain - tubercle bacilli not found (in sputum) by microscopy - but found by bacterial culture |
| 1335 | Tuberculous abscess of brain - tubercle bacilli not found by bacteriological examination - but tuberculosis confirmed histologically |
| 1336 | Tuberculous abscess of brain - tubercle bacilli not found by bacteriological or histological examination - but tuberculosis confirmed by other methods [inoculation of animals] |
| 1340 | Tuberculoma of spinal cord - unspecified |
| 1341 | Tuberculoma of spinal cord - bacteriological or histological examination not done |
| 1342 | Tuberculoma of spinal cord - bacteriological or histological examination unknown (at present) |
| 1343 | Tuberculoma of spinal cord - tubercle bacilli found (in sputum) by microscopy |
| 1344 | Tuberculoma of spinal cord - tubercle bacilli not found (in sputum) by microscopy - but found by bacterial culture |
| 1345 | Tuberculoma of spinal cord - tubercle bacilli not found by bacteriological examination - but tuberculosis confirmed histologically |
| 1346 | Tuberculoma of spinal cord - tubercle bacilli not found by bacteriological or histological examination - but tuberculosis confirmed by other methods [inoculation of animals] |
| 1350 | Tuberculous abscess of spinal cord - unspecified |
| 1351 | Tuberculous abscess of spinal cord - bacteriological or histological examination not done |
| 1352 | Tuberculous abscess of spinal cord - bacteriological or histological examination unknown (at present) |
| 1353 | Tuberculous abscess of spinal cord - tubercle bacilli found (in sputum) by microscopy |
| 1354 | Tuberculous abscess of spinal cord - tubercle bacilli not found (in sputum) by microscopy - but found by bacterial culture |
| 1355 | Tuberculous abscess of spinal cord - tubercle bacilli not found by bacteriological examination - but tuberculosis confirmed histologically |
| 1356 | Tuberculous abscess of spinal cord - tubercle bacilli not found by bacteriological or histological examination - but tuberculosis confirmed by other methods [inoculation of animals] |
| 1360 | Tuberculous encephalitis or myelitis - unspecified |
| 1361 | Tuberculous encephalitis or myelitis - bacteriological or histological examination not done |
| 1362 | Tuberculous encephalitis or myelitis - bacteriological or histological examination unknown (at present) |
| 1363 | Tuberculous encephalitis or myelitis - tubercle bacilli found (in sputum) by microscopy |
| 1363 | Pneumocystosis |
| 1364 | Tuberculous encephalitis or myelitis - tubercle bacilli not found (in sputum) by microscopy - but found by bacterial culture |
| 1365 | Tuberculous encephalitis or myelitis - tubercle bacilli not found by bacteriological examination - but tuberculosis confirmed histologically |
| 1366 | Tuberculous encephalitis or myelitis - tubercle bacilli not found by bacteriological or histological examination - but tuberculosis confirmed by other methods [inoculation of animals] |
| 1380 | Other specified tuberculosis of central nervous system - unspecified |
| 1381 | Other specified tuberculosis of central nervous system - bacteriological or histological examination not done |
| 1382 | Other specified tuberculosis of central nervous system - bacteriological or histological examination unknown (at present) |
| 1383 | Other specified tuberculosis of central nervous system - tubercle bacilli found (in sputum) by microscopy |
| 1384 | Other specified tuberculosis of central nervous system - tubercle bacilli not found (in sputum) by microscopy - but found by bacterial culture |
| 1385 | Other specified tuberculosis of central nervous system - tubercle bacilli not found by bacteriological examination - but tuberculosis confirmed histologically |
| 1386 | Other specified tuberculosis of central nervous system - tubercle bacilli not found by bacteriological or histological examination - but tuberculosis confirmed by other methods [inoculation of animals] |
| 1390 | Unspecified tuberculosis of central nervous system - unspecified |
| 1391 | Unspecified tuberculosis of central nervous system - bacteriological or histological examination not done |
| 1392 | Unspecified tuberculosis of central nervous system - bacteriological or histological examination unknown (at present) |
| 1393 | Unspecified tuberculosis of central nervous system - tubercle bacilli found (in sputum) by microscopy |
| 1394 | Unspecified tuberculosis of central nervous system - tubercle bacilli not found (in sputum) by microscopy - but found by bacterial culture |
| 1395 | Unspecified tuberculosis of central nervous system - tubercle bacilli not found by bacteriological examination - but tuberculosis confirmed histologically |
| 1396 | Unspecified tuberculosis of central nervous system - tubercle bacilli not found by bacteriological or histological examination - but tuberculosis confirmed by other methods [inoculation of animals] |
| 1400 | Tuberculous peritonitis - unspecified |
| 1401 | Tuberculous peritonitis - bacteriological or histological examination not done |
| 1402 | Tuberculous peritonitis - bacteriological or histological examination unknown (at present) |
| 1403 | Tuberculous peritonitis - tubercle bacilli found (in sputum) by microscopy |
| 1404 | Tuberculous peritonitis - tubercle bacilli not found (in sputum) by microscopy - but found by bacterial culture |
| 1405 | Tuberculous peritonitis - tubercle bacilli not found by bacteriological examination - but tuberculosis confirmed histologically |
| 1406 | Tuberculous peritonitis - tubercle bacilli not found by bacteriological or histological examination - but tuberculosis confirmed by other methods [inoculation of animals] |
| 1480 | Other tuberculosis of intestines - peritoneum - and mesenteric glands - unspecified |
| 1481 | Other tuberculosis of intestines - peritoneum - and mesenteric glands - bacteriological or histological examination not done |
| 1482 | Other tuberculosis of intestines - peritoneum - and mesenteric glands - bacteriological or histological examination unknown (at present) |
| 1483 | Other tuberculosis of intestines - peritoneum - and mesenteric glands - tubercle bacilli found (in sputum) by microscopy |
| 1484 | Other tuberculosis of intestines - peritoneum - and mesenteric glands - tubercle bacilli not found (in sputum) by microscopy - but found by bacterial culture |
| 1485 | Other tuberculosis of intestines - peritoneum - and mesenteric glands - tubercle bacilli not found by bacteriological examination - but tuberculosis confirmed histologically |
| 1486 | Other tuberculosis of intestines - peritoneum - and mesenteric glands - tubercle bacilli not found by bacteriological or histological examination - but tuberculosis confirmed by other methods [inoculation of animals] |
| 1500 | Tuberculosis of vertebral column - unspecified |
| 1501 | Tuberculosis of vertebral column - bacteriological or histological examination not done |
| 1502 | Tuberculosis of vertebral column - bacteriological or histological examination unknown (at present) |
| 1503 | Tuberculosis of vertebral column - tubercle bacilli found (in sputum) by microscopy |
| 1504 | Tuberculosis of vertebral column - tubercle bacilli not found (in sputum) by microscopy - but found by bacterial culture |
| 1505 | Tuberculosis of vertebral column - tubercle bacilli not found by bacteriological examination - but tuberculosis confirmed histologically |
| 1506 | Tuberculosis of vertebral column - tubercle bacilli not found by bacteriological or histological examination - but tuberculosis confirmed by other methods [inoculation of animals] |
| 1510 | Tuberculosis of hip - unspecified |
| 1511 | Tuberculosis of hip - bacteriological or histological examination not done |
| 1512 | Tuberculosis of hip - bacteriological or histological examination unknown (at present) |
| 1513 | Tuberculosis of hip - tubercle bacilli found (in sputum) by microscopy |
| 1514 | Tuberculosis of hip - tubercle bacilli not found (in sputum) by microscopy - but found by bacterial culture |
| 1515 | Tuberculosis of hip - tubercle bacilli not found by bacteriological examination - but tuberculosis confirmed histologically |
| 1516 | Tuberculosis of hip - tubercle bacilli not found by bacteriological or histological examination - but tuberculosis confirmed by other methods [inoculation of animals] |
| 1520 | Tuberculosis of knee - unspecified |
| 1521 | Tuberculosis of knee - bacteriological or histological examination not done |
| 1522 | Tuberculosis of knee - bacteriological or histological examination unknown (at present) |
| 1523 | Tuberculosis of knee - tubercle bacilli found (in sputum) by microscopy |
| 1524 | Tuberculosis of knee - tubercle bacilli not found (in sputum) by microscopy - but found by bacterial culture |
| 1525 | Tuberculosis of knee - tubercle bacilli not found by bacteriological examination - but tuberculosis confirmed histologically |
| 1526 | Tuberculosis of knee - tubercle bacilli not found by bacteriological or histological examination - but tuberculosis confirmed by other methods [inoculation of animals] |
| 1550 | Tuberculosis of limb bones - unspecified |
| 1551 | Tuberculosis of limb bones - bacteriological or histological examination not done |
| 1552 | Tuberculosis of limb bones - bacteriological or histological examination unknown (at present) |
| 1553 | Tuberculosis of limb bones - tubercle bacilli found (in sputum) by microscopy |
| 1554 | Tuberculosis of limb bones - tubercle bacilli not found (in sputum) by microscopy - but found by bacterial culture |
| 1555 | Tuberculosis of limb bones - tubercle bacilli not found by bacteriological examination - but tuberculosis confirmed histologically |
| 1556 | Tuberculosis of limb bones - tubercle bacilli not found by bacteriological or histological examination - but tuberculosis confirmed by other methods [inoculation of animals] |
| 1560 | Tuberculosis of mastoid - unspecified |
| 1561 | Tuberculosis of mastoid - bacteriological or histological examination not done |
| 1562 | Tuberculosis of mastoid - bacteriological or histological examination unknown (at present) |
| 1563 | Tuberculosis of mastoid - tubercle bacilli found (in sputum) by microscopy |
| 1564 | Tuberculosis of mastoid - tubercle bacilli not found (in sputum) by microscopy - but found by bacterial culture |
| 1565 | Tuberculosis of mastoid - tubercle bacilli not found by bacteriological examination - but tuberculosis confirmed histologically |
| 1566 | Tuberculosis of mastoid - tubercle bacilli not found by bacteriological or histological examination - but tuberculosis confirmed by other methods [inoculation of animals] |
| 1570 | Tuberculosis of other specified bone - unspecified |
| 1571 | Tuberculosis of other specified bone - bacteriological or histological examination not done |
| 1572 | Tuberculosis of other specified bone - bacteriological or histological examination unknown (at present) |
| 1573 | Tuberculosis of other specified bone - tubercle bacilli found (in sputum) by microscopy |
| 1574 | Tuberculosis of other specified bone - tubercle bacilli not found (in sputum) by microscopy - but found by bacterial culture |
| 1575 | Tuberculosis of other specified bone - tubercle bacilli not found by bacteriological examination - but tuberculosis confirmed histologically |
| 1576 | Tuberculosis of other specified bone - tubercle bacilli not found by bacteriological or histological examination - but tuberculosis confirmed by other methods [inoculation of animals] |
| 1580 | Tuberculosis of other specified joint - unspecified |
| 1581 | Tuberculosis of other specified joint - bacteriological or histological examination not done |
| 1582 | Tuberculosis of other specified joint - bacteriological or histological examination unknown (at present) |
| 1583 | Tuberculosis of other specified joint - tubercle bacilli found (in sputum) by microscopy |
| 1584 | Tuberculosis of other specified joint - tubercle bacilli not found (in sputum) by microscopy - but found by bacterial culture |
| 1585 | Tuberculosis of other specified joint - tubercle bacilli not found by bacteriological examination - but tuberculosis confirmed histologically |
| 1586 | Tuberculosis of other specified joint - tubercle bacilli not found by bacteriological or histological examination - but tuberculosis confirmed by other methods [inoculation of animals] |
| 1590 | Tuberculosis of unspecified bones and joints - unspecified |
| 1591 | Tuberculosis of unspecified bones and joints - bacteriological or histological examination not done |
| 1592 | Tuberculosis of unspecified bones and joints - bacteriological or histological examination unknown (at present) |
| 1593 | Tuberculosis of unspecified bones and joints - tubercle bacilli found (in sputum) by microscopy |
| 1594 | Tuberculosis of unspecified bones and joints - tubercle bacilli not found (in sputum) by microscopy - but found by bacterial culture |
| 1595 | Tuberculosis of unspecified bones and joints - tubercle bacilli not found by bacteriological examination - but tuberculosis confirmed histologically |
| 1596 | Tuberculosis of unspecified bones and joints - tubercle bacilli not found by bacteriological or histological examination - but tuberculosis confirmed by other methods [inoculation of animals] |
| 1600 | Tuberculosis of kidney - unspecified |
| 1601 | Tuberculosis of kidney - bacteriological or histological examination not done |
| 1602 | Tuberculosis of kidney - bacteriological or histological examination unknown (at present) |
| 1603 | Tuberculosis of kidney - tubercle bacilli found (in sputum) by microscopy |
| 1604 | Tuberculosis of kidney - tubercle bacilli not found (in sputum) by microscopy - but found by bacterial culture |
| 1605 | Tuberculosis of kidney - tubercle bacilli not found by bacteriological examination - but tuberculosis confirmed histologically |
| 1606 | Tuberculosis of kidney - tubercle bacilli not found by bacteriological or histological examination - but tuberculosis confirmed by other methods [inoculation of animals] |
| 1610 | Tuberculosis of bladder - unspecified |
| 1611 | Tuberculosis of bladder - bacteriological or histological examination not done |
| 1612 | Tuberculosis of bladder - bacteriological or histological examination unknown (at present) |
| 1613 | Tuberculosis of bladder - tubercle bacilli found (in sputum) by microscopy |
| 1614 | Tuberculosis of bladder - tubercle bacilli not found (in sputum) by microscopy - but found by bacterial culture |
| 1615 | Tuberculosis of bladder - tubercle bacilli not found by bacteriological examination - but tuberculosis confirmed histologically |
| 1616 | Tuberculosis of bladder - tubercle bacilli not found by bacteriological or histological examination - but tuberculosis confirmed by other methods [inoculation of animals] |
| 1620 | Tuberculosis of ureter - unspecified |
| 1621 | Tuberculosis of ureter - bacteriological or histological examination not done |
| 1622 | Tuberculosis of ureter - bacteriological or histological examination unknown (at present) |
| 1623 | Tuberculosis of ureter - tubercle bacilli found (in sputum) by microscopy |
| 1624 | Tuberculosis of ureter - tubercle bacilli not found (in sputum) by microscopy - but found by bacterial culture |
| 1625 | Tuberculosis of ureter - tubercle bacilli not found by bacteriological examination - but tuberculosis confirmed histologically |
| 1626 | Tuberculosis of ureter - tubercle bacilli not found by bacteriological or histological examination - but tuberculosis confirmed by other methods [inoculation of animals] |
| 1630 | Tuberculosis of other urinary organs - unspecified |
| 1631 | Tuberculosis of other urinary organs - bacteriological or histological examination not done |
| 1632 | Tuberculosis of other urinary organs - bacteriological or histological examination unknown (at present) |
| 1633 | Tuberculosis of other urinary organs - tubercle bacilli found (in sputum) by microscopy |
| 1634 | Tuberculosis of other urinary organs - tubercle bacilli not found (in sputum) by microscopy - but found by bacterial culture |
| 1635 | Tuberculosis of other urinary organs - tubercle bacilli not found by bacteriological examination - but tuberculosis confirmed histologically |
| 1636 | Tuberculosis of other urinary organs - tubercle bacilli not found by bacteriological or histological examination - but tuberculosis confirmed by other methods [inoculation of animals] |
| 1640 | Tuberculosis of epididymis - unspecified |
| 1641 | Tuberculosis of epididymis - bacteriological or histological examination not done |
| 1642 | Tuberculosis of epididymis - bacteriological or histological examination unknown (at present) |
| 1643 | Tuberculosis of epididymis - tubercle bacilli found (in sputum) by microscopy |
| 1644 | Tuberculosis of epididymis - tubercle bacilli not found (in sputum) by microscopy - but found by bacterial culture |
| 1645 | Tuberculosis of epididymis - tubercle bacilli not found by bacteriological examination - but tuberculosis confirmed histologically |
| 1646 | Tuberculosis of epididymis - tubercle bacilli not found by bacteriological or histological examination - but tuberculosis confirmed by other methods [inoculation of animals] |
| 1650 | Tuberculosis of other male genital organs - unspecified |
| 1651 | Tuberculosis of other male genital organs - bacteriological or histological examination not done |
| 1652 | Tuberculosis of other male genital organs - bacteriological or histological examination unknown (at present) |
| 1653 | Tuberculosis of other male genital organs - tubercle bacilli found (in sputum) by microscopy |
| 1654 | Tuberculosis of other male genital organs - tubercle bacilli not found (in sputum) by microscopy - but found by bacterial culture |
| 1655 | Tuberculosis of other male genital organs - tubercle bacilli not found by bacteriological examination - but tuberculosis confirmed histologically |
| 1656 | Tuberculosis of other male genital organs - tubercle bacilli not found by bacteriological or histological examination - but tuberculosis confirmed by other methods [inoculation of animals] |
| 1660 | Tuberculous oophoritis and salpingitis - unspecified |
| 1661 | Tuberculous oophoritis and salpingitis - bacteriological or histological examination not done |
| 1662 | Tuberculous oophoritis and salpingitis - bacteriological or histological examination unknown (at present) |
| 1663 | Tuberculous oophoritis and salpingitis - tubercle bacilli found (in sputum) by microscopy |
| 1664 | Tuberculous oophoritis and salpingitis - tubercle bacilli not found (in sputum) by microscopy - but found by bacterial culture |
| 1665 | Tuberculous oophoritis and salpingitis - tubercle bacilli not found by bacteriological examination - but tuberculosis confirmed histologically |
| 1666 | Tuberculous oophoritis and salpingitis - tubercle bacilli not found by bacteriological or histological examination - but tuberculosis confirmed by other methods [inoculation of animals] |
| 1670 | Tuberculosis of other female genital organs - unspecified |
| 1671 | Tuberculosis of other female genital organs - bacteriological or histological examination not done |
| 1672 | Tuberculosis of other female genital organs - bacteriological or histological examination unknown (at present) |
| 1673 | Tuberculosis of other female genital organs - tubercle bacilli found (in sputum) by microscopy |
| 1674 | Tuberculosis of other female genital organs - tubercle bacilli not found (in sputum) by microscopy - but found by bacterial culture |
| 1675 | Tuberculosis of other female genital organs - tubercle bacilli not found by bacteriological examination - but tuberculosis confirmed histologically |
| 1676 | Tuberculosis of other female genital organs - tubercle bacilli not found by bacteriological or histological examination - but tuberculosis confirmed by other methods [inoculation of animals] |
| 1690 | Genitourinary tuberculosis - unspecified - unspecified |
| 1691 | Genitourinary tuberculosis - unspecified - bacteriological or histological examination not done |
| 1692 | Genitourinary tuberculosis - unspecified - bacteriological or histological examination unknown (at present) |
| 1693 | Genitourinary tuberculosis - unspecified - tubercle bacilli found (in sputum) by microscopy |
| 1694 | Genitourinary tuberculosis - unspecified - tubercle bacilli not found (in sputum) by microscopy - but found by bacterial culture |
| 1695 | Genitourinary tuberculosis - unspecified - tubercle bacilli not found by bacteriological examination - but tuberculosis confirmed histologically |
| 1696 | Genitourinary tuberculosis - unspecified - tubercle bacilli not found by bacteriological or histological examination - but tuberculosis confirmed by other methods [inoculation of animals] |
| 1700 | Tuberculosis of skin and subcutaneous cellular tissue - unspecified |
| 1701 | Tuberculosis of skin and subcutaneous cellular tissue - bacteriological or histological examination not done |
| 1702 | Tuberculosis of skin and subcutaneous cellular tissue - bacteriological or histological examination unknown (at present) |
| 1703 | Tuberculosis of skin and subcutaneous cellular tissue - tubercle bacilli found (in sputum) by microscopy |
| 1704 | Tuberculosis of skin and subcutaneous cellular tissue - tubercle bacilli not found (in sputum) by microscopy - but found by bacterial culture |
| 1705 | Tuberculosis of skin and subcutaneous cellular tissue - tubercle bacilli not found by bacteriological examination - but tuberculosis confirmed histologically |
| 1706 | Tuberculosis of skin and subcutaneous cellular tissue - tubercle bacilli not found by bacteriological or histological examination - but tuberculosis confirmed by other methods [inoculation of animals] |
| 1710 | Erythema nodosum with hypersensitivity reaction in tuberculosis - unspecified |
| 1711 | Erythema nodosum with hypersensitivity reaction in tuberculosis - bacteriological or histological examination not done |
| 1712 | Erythema nodosum with hypersensitivity reaction in tuberculosis - bacteriological or histological examination unknown (at present) |
| 1713 | Erythema nodosum with hypersensitivity reaction in tuberculosis - tubercle bacilli found (in sputum) by microscopy |
| 1714 | Erythema nodosum with hypersensitivity reaction in tuberculosis - tubercle bacilli not found (in sputum) by microscopy - but found by bacterial culture |
| 1715 | Erythema nodosum with hypersensitivity reaction in tuberculosis - tubercle bacilli not found by bacteriological examination - but tuberculosis confirmed histologically |
| 1716 | Erythema nodosum with hypersensitivity reaction in tuberculosis - tubercle bacilli not found by bacteriological or histological examination - but tuberculosis confirmed by other methods [inoculation of animals] |
| 1720 | Tuberculosis of peripheral lymph nodes - unspecified |
| 1721 | Tuberculosis of peripheral lymph nodes - bacteriological or histological examination not done |
| 1722 | Tuberculosis of peripheral lymph nodes - bacteriological or histological examination unknown (at present) |
| 1723 | Tuberculosis of peripheral lymph nodes - tubercle bacilli found (in sputum) by microscopy |
| 1724 | Tuberculosis of peripheral lymph nodes - tubercle bacilli not found (in sputum) by microscopy - but found by bacterial culture |
| 1725 | Tuberculosis of peripheral lymph nodes - tubercle bacilli not found by bacteriological examination - but tuberculosis confirmed histologically |
| 1726 | Tuberculosis of peripheral lymph nodes - tubercle bacilli not found by bacteriological or histological examination - but tuberculosis confirmed by other methods [inoculation of animals] |
| 1730 | Tuberculosis of eye - unspecified |
| 1731 | Tuberculosis of eye - bacteriological or histological examination not done |
| 1732 | Tuberculosis of eye - bacteriological or histological examination unknown (at present) |
| 1733 | Tuberculosis of eye - tubercle bacilli found (in sputum) by microscopy |
| 1734 | Tuberculosis of eye - tubercle bacilli not found (in sputum) by microscopy - but found by bacterial culture |
| 1735 | Tuberculosis of eye - tubercle bacilli not found by bacteriological examination - but tuberculosis confirmed histologically |
| 1736 | Tuberculosis of eye - tubercle bacilli not found by bacteriological or histological examination - but tuberculosis confirmed by other methods [inoculation of animals] |
| 1740 | Tuberculosis of ear - unspecified |
| 1741 | Tuberculosis of ear - bacteriological or histological examination not done |
| 1742 | Tuberculosis of ear - bacteriological or histological examination unknown (at present) |
| 1743 | Tuberculosis of ear - tubercle bacilli found (in sputum) by microscopy |
| 1744 | Tuberculosis of ear - tubercle bacilli not found (in sputum) by microscopy - but found by bacterial culture |
| 1745 | Tuberculosis of ear - tubercle bacilli not found by bacteriological examination - but tuberculosis confirmed histologically |
| 1746 | Tuberculosis of ear - tubercle bacilli not found by bacteriological or histological examination - but tuberculosis confirmed by other methods [inoculation of animals] |
| 1750 | Tuberculosis of thyroid gland - unspecified |
| 1751 | Tuberculosis of thyroid gland - bacteriological or histological examination not done |
| 1752 | Tuberculosis of thyroid gland - bacteriological or histological examination unknown (at present) |
| 1753 | Tuberculosis of thyroid gland - tubercle bacilli found (in sputum) by microscopy |
| 1754 | Tuberculosis of thyroid gland - tubercle bacilli not found (in sputum) by microscopy - but found by bacterial culture |
| 1755 | Tuberculosis of thyroid gland - tubercle bacilli not found by bacteriological examination - but tuberculosis confirmed histologically |
| 1756 | Tuberculosis of thyroid gland - tubercle bacilli not found by bacteriological or histological examination - but tuberculosis confirmed by other methods [inoculation of animals] |
| 1760 | Tuberculosis of adrenal glands - unspecified |
| 1761 | Tuberculosis of adrenal glands - bacteriological or histological examination not done |
| 1762 | Tuberculosis of adrenal glands - bacteriological or histological examination unknown (at present) |
| 1763 | Tuberculosis of adrenal glands - tubercle bacilli found (in sputum) by microscopy |
| 1764 | Tuberculosis of adrenal glands - tubercle bacilli not found (in sputum) by microscopy - but found by bacterial culture |
| 1765 | Tuberculosis of adrenal glands - tubercle bacilli not found by bacteriological examination - but tuberculosis confirmed histologically |
| 1766 | Tuberculosis of adrenal glands - tubercle bacilli not found by bacteriological or histological examination - but tuberculosis confirmed by other methods [inoculation of animals] |
| 1770 | Tuberculosis of spleen - unspecified |
| 1771 | Tuberculosis of spleen - bacteriological or histological examination not done |
| 1772 | Tuberculosis of spleen - bacteriological or histological examination unknown (at present) |
| 1773 | Tuberculosis of spleen - tubercle bacilli found (in sputum) by microscopy |
| 1774 | Tuberculosis of spleen - tubercle bacilli not found (in sputum) by microscopy - but found by bacterial culture |
| 1775 | Tuberculosis of spleen - tubercle bacilli not found by bacteriological examination - but tuberculosis confirmed histologically |
| 1776 | Tuberculosis of spleen - tubercle bacilli not found by bacteriological or histological examination - but tuberculosis confirmed by other methods [inoculation of animals] |
| 1780 | Tuberculosis of esophagus - unspecified |
| 1781 | Tuberculosis of esophagus - bacteriological or histological examination not done |
| 1782 | Tuberculosis of esophagus - bacteriological or histological examination unknown (at present) |
| 1783 | Tuberculosis of esophagus - tubercle bacilli found (in sputum) by microscopy |
| 1784 | Tuberculosis of esophagus - tubercle bacilli not found (in sputum) by microscopy - but found by bacterial culture |
| 1785 | Tuberculosis of esophagus - tubercle bacilli not found by bacteriological examination - but tuberculosis confirmed histologically |
| 1786 | Tuberculosis of esophagus - tubercle bacilli not found by bacteriological or histological examination - but tuberculosis confirmed by other methods [inoculation of animals] |
| 1790 | Tuberculosis of other specified organs - unspecified |
| 1791 | Tuberculosis of other specified organs - bacteriological or histological examination not done |
| 1792 | Tuberculosis of other specified organs - bacteriological or histological examination unknown (at present) |
| 1793 | Tuberculosis of other specified organs - tubercle bacilli found (in sputum) by microscopy |
| 1794 | Tuberculosis of other specified organs - tubercle bacilli not found (in sputum) by microscopy - but found by bacterial culture |
| 1795 | Tuberculosis of other specified organs - tubercle bacilli not found by bacteriological examination - but tuberculosis confirmed histologically |
| 1796 | Tuberculosis of other specified organs - tubercle bacilli not found by bacteriological or histological examination - but tuberculosis confirmed by other methods [inoculation of animals] |
| 1800 | Acute miliary tuberculosis - unspecified |
| 1801 | Acute miliary tuberculosis - bacteriological or histological examination not done |
| 1802 | Acute miliary tuberculosis - bacteriological or histological examination unknown (at present) |
| 1803 | Acute miliary tuberculosis - tubercle bacilli found (in sputum) by microscopy |
| 1804 | Acute miliary tuberculosis - tubercle bacilli not found (in sputum) by microscopy - but found by bacterial culture |
| 1805 | Acute miliary tuberculosis - tubercle bacilli not found by bacteriological examination - but tuberculosis confirmed histologically |
| 1806 | Acute miliary tuberculosis - tubercle bacilli not found by bacteriological or histological examination - but tuberculosis confirmed by other methods [inoculation of animals] |
| 1880 | Other specified miliary tuberculosis - unspecified |
| 1881 | Other specified miliary tuberculosis - bacteriological or histological examination not done |
| 1882 | Other specified miliary tuberculosis - bacteriological or histological examination unknown (at present) |
| 1883 | Other specified miliary tuberculosis - tubercle bacilli found (in sputum) by microscopy |
| 1884 | Other specified miliary tuberculosis - tubercle bacilli not found (in sputum) by microscopy - but found by bacterial culture |
| 1885 | Other specified miliary tuberculosis - tubercle bacilli not found by bacteriological examination - but tuberculosis confirmed histologically |
| 1886 | Other specified miliary tuberculosis - tubercle bacilli not found by bacteriological or histological examination - but tuberculosis confirmed by other methods [inoculation of animals] |
| 1890 | Miliary tuberculosis - unspecified - unspecified |
| 1891 | Miliary tuberculosis - unspecified - bacteriological or histological examination not done |
| 1892 | Miliary tuberculosis - unspecified - bacteriological or histological examination unknown (at present) |
| 1893 | Miliary tuberculosis - unspecified - tubercle bacilli found (in sputum) by microscopy |
| 1894 | Miliary tuberculosis - unspecified - tubercle bacilli not found (in sputum) by microscopy - but found by bacterial culture |
| 1895 | Miliary tuberculosis - unspecified - tubercle bacilli not found by bacteriological examination - but tuberculosis confirmed histologically |
| 1896 | Miliary tuberculosis - unspecified - tubercle bacilli not found by bacteriological or histological examination - but tuberculosis confirmed by other methods [inoculation of animals] |
| 2792 | Combined immunity deficiency |
| 2793 | Unspecified immunity deficiency |
| 2798 | Other specified disorders involving the immune mechanism |
| 2799 | Unspecified disorder of immune mechanism |
| 2881 | Functional disorders of polymorphonuclear neutrophils |
| 2882 | Genetic anomalies of leukocytes |
| 3210 | Cryptococcal meningitis |
| 4841 | Pneumonia in cytomegalic inclusion disease |
| 4846 | Pneumonia in aspergillosis |
| 11281 | Candidal endocarditis |
| 11282 | Candidal otitis externa |
| 11283 | Candidal meningitis |
| 11284 | Candidal esophagitis |
| 11285 | Candidal enteritis |
| 11289 | Other candidiasis of other specified sites |
| 27900 | Hypogammaglobulinemia - unspecified |
| 27901 | Selective IgA immunodeficiency |
| 27902 | Selective IgM immunodeficiency |
| 27903 | Other selective immunoglobulin deficiencies |
| 27904 | Congenital hypogammaglobulinemia |
| 27905 | Immunodeficiency with increased IgM |
| 27906 | Common variable immunodeficiency |
| 27909 | Other deficiency of humoral immunity |
| 27910 | Immunodeficiency with predominant T-cell defect - unspecified |
| 27911 | Digeorge's syndrome |
| 27912 | Wiskott-aldrich syndrome |
| 27913 | Nezelof's syndrome |
| 27919 | Other deficiency of cell-mediated immunity |
| 27941 | Autoimmune lymphoproliferative syndrome |
| 27949 | Autoimmune disease - not elsewhere classified |
| 27950 | Graft-versus-host disease - unspecified |
| 27951 | Acute graft-versus-host disease |
| 27952 | Chronic graft-versus-host disease |
| 27953 | Acute on chronic graft-versus-host disease |
| **Immunocompromised-Related ICD-10 Codes** | |
| A072 | Cryptosporidiosis |
| A150 | Tuberculosis of lung |
| A154 | Tuberculosis of intrathoracic lymph nodes |
| A155 | Tuberculosis of larynx, trachea and bronchus |
| A156 | Tuberculous pleurisy |
| A157 | Primary respiratory tuberculosis |
| A158 | Other respiratory tuberculosis |
| A159 | Respiratory tuberculosis unspecified |
| A170 | Tuberculous meningitis |
| A171 | Meningeal tuberculoma |
| A1781 | Tuberculoma of brain and spinal cord |
| A1782 | Tuberculous meningoencephalitis |
| A1783 | Tuberculous neuritis |
| A1789 | Other tuberculosis of nervous system |
| A179 | Tuberculosis of nervous system, unspecified |
| A1801 | Tuberculosis of spine |
| A1802 | Tuberculous arthritis of other joints |
| A1803 | Tuberculosis of other bones |
| A1809 | Other musculoskeletal tuberculosis |
| A1810 | Tuberculosis of genitourinary system, unspecified |
| A1811 | Tuberculosis of kidney and ureter |
| A1812 | Tuberculosis of bladder |
| A1813 | Tuberculosis of other urinary organs |
| A1814 | Tuberculosis of prostate |
| A1815 | Tuberculosis of other male genital organs |
| A1816 | Tuberculosis of cervix |
| A1817 | Tuberculous female pelvic inflammatory disease |
| A1818 | Tuberculosis of other female genital organs |
| A182 | Tuberculous peripheral lymphadenopathy |
| A1831 | Tuberculous peritonitis |
| A1832 | Tuberculous enteritis |
| A1839 | Retroperitoneal tuberculosis |
| A184 | Tuberculosis of skin and subcutaneous tissue |
| A1850 | Tuberculosis of eye, unspecified |
| A1851 | Tuberculous episcleritis |
| A1852 | Tuberculous keratitis |
| A1853 | Tuberculous chorioretinitis |
| A1854 | Tuberculous iridocyclitis |
| A1859 | Other tuberculosis of eye |
| A186 | Tuberculosis of (inner) (middle) ear |
| A187 | Tuberculosis of adrenal glands |
| A1881 | Tuberculosis of thyroid gland |
| A1882 | Tuberculosis of other endocrine glands |
| A1883 | Tuberculosis of digestive tract organs, not elsewhere classified |
| A1884 | Tuberculosis of heart |
| A1885 | Tuberculosis of spleen |
| A1889 | Tuberculosis of other sites |
| A190 | Acute miliary tuberculosis of a single specified site |
| A191 | Acute miliary tuberculosis of multiple sites |
| A192 | Acute miliary tuberculosis, unspecified |
| A198 | Other miliary tuberculosis |
| A199 | Miliary tuberculosis, unspecified |
| B20 | Human immunodeficiency virus [HIV] disease |
| B250 | Cytomegaloviral pneumonitis |
| B251 | Cytomegaloviral hepatitis |
| B252 | Cytomegaloviral pancreatitis |
| B258 | Other cytomegaloviral diseases |
| B259 | Cytomegaloviral disease, unspecified |
| B370 | Candidal stomatitis |
| B371 | Pulmonary candidiasis |
| B372 | Candidiasis of skin and nail |
| B373 | Candidiasis of vulva and vagina |
| B3741 | Candidal cystitis and urethritis |
| B3742 | Candidal balanitis |
| B3749 | Other urogenital candidiasis |
| B375 | Candidal meningitis |
| B376 | Candidal endocarditis |
| B377 | Candidal sepsis |
| B3781 | Candidal esophagitis |
| B3782 | Candidal enteritis |
| B3783 | Candidal cheilitis |
| B3784 | Candidal otitis externa |
| B3789 | Other sites of candidiasis |
| B379 | Candidiasis, unspecified |
| B440 | Invasive pulmonary aspergillosis |
| B441 | Other pulmonary aspergillosis |
| B442 | Tonsillar aspergillosis |
| B447 | Disseminated aspergillosis |
| B4489 | Other forms of aspergillosis |
| B449 | Aspergillosis, unspecified |
| B450 | Pulmonary cryptococcosis |
| B451 | Cerebral cryptococcosis |
| B452 | Cutaneous cryptococcosis |
| B453 | Osseous cryptococcosis |
| B457 | Disseminated cryptococcosis |
| B458 | Other forms of cryptococcosis |
| B459 | Cryptococcosis, unspecified |
| B5800 | Toxoplasma oculopathy, unspecified |
| B5801 | Toxoplasma chorioretinitis |
| B5809 | Other toxoplasma oculopathy |
| B581 | Toxoplasma hepatitis |
| B582 | Toxoplasma meningoencephalitis |
| B583 | Pulmonary toxoplasmosis |
| B5881 | Toxoplasma myocarditis |
| B5882 | Toxoplasma myositis |
| B5883 | Toxoplasma tubulo-interstitial nephropathy |
| B5889 | Toxoplasmosis with other organ involvement |
| B589 | Toxoplasmosis, unspecified |
| B59 | Pneumocystosis |
| D71 | Functional disorders of polymorphonuclear neutrophils |
| D720 | Genetic anomalies of leukocytes |
| D800 | Hereditary hypogammaglobulinemia |
| D801 | Nonfamilial hypogammaglobulinemia |
| D802 | Selective deficiency of immunoglobulin A [IgA] |
| D803 | Selective deficiency of immunoglobulin G [IgG] subclasses |
| D804 | Selective deficiency of immunoglobulin M [IgM] |
| D805 | Immunodeficiency with increased immunoglobulin M [IgM] |
| D806 | Antibody deficiency with near-normal immunoglobulins or with hyperimmunoglobulinemia |
| D807 | Transient hypogammaglobulinemia of infancy |
| D808 | Other immunodeficiencies with predominantly antibody defects |
| D809 | Immunodeficiency with predominantly antibody defects, unspecified |
| D810 | Severe combined immunodeficiency [SCID] with reticular dysgenesis |
| D811 | Severe combined immunodeficiency [SCID] with low T- and B-cell numbers |
| D812 | Severe combined immunodeficiency [SCID] with low or normal B-cell numbers |
| D8130 | Adenosine deaminase deficiency, unspecified |
| D8131 | Severe combined immunodeficiency due to adenosine deaminase deficiency |
| D8132 | Adenosine deaminase 2 deficiency |
| D8139 | Other adenosine deaminase deficiency |
| D814 | Nezelof's syndrome |
| D815 | Purine nucleoside phosphorylase [PNP] deficiency |
| D816 | Major histocompatibility complex class I deficiency |
| D817 | Major histocompatibility complex class II deficiency |
| D81810 | Biotinidase deficiency |
| D81818 | Other biotin-dependent carboxylase deficiency |
| D81819 | Biotin-dependent carboxylase deficiency, unspecified |
| D8189 | Other combined immunodeficiencies |
| D819 | Combined immunodeficiency, unspecified |
| D820 | Wiskott-Aldrich syndrome |
| D821 | Di George's syndrome |
| D822 | Immunodeficiency with short-limbed stature |
| D823 | Immunodeficiency following hereditary defective response to Epstein-Barr virus |
| D824 | Hyperimmunoglobulin E [IgE] syndrome |
| D828 | Immunodeficiency associated with other specified major defects |
| D829 | Immunodeficiency associated with major defect, unspecified |
| D830 | Common variable immunodeficiency with predominant abnormalities of B-cell numbers and function |
| D831 | Common variable immunodeficiency with predominant immunoregulatory T-cell disorders |
| D832 | Common variable immunodeficiency with autoantibodies to B- or T-cells |
| D838 | Other common variable immunodeficiencies |
| D839 | Common variable immunodeficiency, unspecified |
| D840 | Lymphocyte function antigen-1 [LFA-1] defect |
| D841 | Defects in the complement system |
| D848 | Other specified immunodeficiencies |
| D849 | Immunodeficiency, unspecified |
| D860 | Sarcoidosis of lung |
| D861 | Sarcoidosis of lymph nodes |
| D862 | Sarcoidosis of lung with sarcoidosis of lymph nodes |
| D863 | Sarcoidosis of skin |
| D8681 | Sarcoid meningitis |
| D8682 | Multiple cranial nerve palsies in sarcoidosis |
| D8683 | Sarcoid iridocyclitis |
| D8684 | Sarcoid pyelonephritis |
| D8685 | Sarcoid myocarditis |
| D8686 | Sarcoid arthropathy |
| D8687 | Sarcoid myositis |
| D8689 | Sarcoidosis of other sites |
| D869 | Sarcoidosis, unspecified |
| E70330 | Chediak-Higashi syndrome |
